# Inhibition of SIRT3 by a specific inhibitor induces cellular senescence and growth arrest of ovarian granulosa cell tumor via p53 and NF-κB axis

**DOI:** 10.3389/fphar.2025.1608156

**Published:** 2025-07-24

**Authors:** Jingxin Ma, Sailing Lin, Chuimian Zeng, Wenhao Wu, Qi Zhang, Guli Zhu, Qi Zhang, Qiongfang Fang, Lijun Fan, Shunichi Takeda, Xiaoyu Li, Xiushen Li, Yu Zhou, Xueqing Wu

**Affiliations:** ^1^School of Biomedical Engineering, Shenzhen University Medical School, Shenzhen University, Shenzhen, China; ^2^Shenzhen University Medical School, Shenzhen University, Shenzhen, China; ^3^Zhongshan School of Medicine, Sun Yat-sen University, Guangzhou, China; ^4^Department of Chemistry and State Key Laboratory of Synthetic Chemistry, The University of Hong Kong, Pokfulam, Hong Kong SAR, China; ^5^Laboratory for Synthetic Chemistry and Chemical Biology Limited, Health@InnoHK, Innovation and Technology Commission, Pokfulam, Hong Kong SAR, China; ^6^Department of Traditional Chinese Medicine, Jiangxi Maternal and Child Health Hospital, Nanchang, China; ^7^School of Basic Medicine and Clinical Pharmacy, China Pharmaceutical University, Nanjing, China; ^8^Department of Obstetrics and Gynecology, Shenzhen University General Hospital, Shenzhen, China

**Keywords:** ovarian granulosa cell tumor, SIRT3 inhibitor, p53, NF-κB, senescence

## Abstract

**Objectives:**

Ovarian granulosa cell tumors (GCTs) are rare ovarian malignancies with limited therapeutic options, particularly in advanced stages. SIRT3, an NAD + -dependent deacetylase, is upregulated in GCTs and implicated in tumorigenesis, yet its functional role and underlying mechanisms remain poorly understood. This study aims to investigate the therapeutic efficacy of a novel SIRT3-specific inhibitor, 77-39, in GCTs by targeting SIRT3 and to elucidate the molecular mechanisms underlying its effects.

**Methods:**

This study investigated the effects of a SIRT3-specific inhibitor, 77-39, on GCT cell growth and explored its underlying mechanisms. Using human GCT cell lines KGN and COV434, we assessed the impact of 77–39 on cell viability and proliferation. RNA sequencing and gene set enrichment analyses were performed to elucidate the pathways affected by 77–39. Western blot assays were used to confirm the activation of specific signaling pathways. Additionally, SIRT3 was silenced or overexpressed to observe the corresponding effects on GCT cells. *In vivo* studies were conducted using xenograft tumor models to evaluate the efficacy and toxicity of 77–39 compared to cisplatin.

**Results:**

We demonstrated that 77–39 significantly suppressed cell viability and proliferation while inducing cellular senescence in human GCT cell lines KGN and COV434. RNA sequencing and gene set enrichment analyses revealed that 77–39 led to the activation of the p53 and NF-κB signaling pathways, which were confirmed by Western blot assay. Silencing SIRT3 recapitulated the effects of 77–39, while SIRT3 overexpression reversed these effects. Inhibition of p53 or NF-κB rescued GCT cells from 77-39-induced growth arrest and senescence. *In vivo* studies using xenograft tumor models showed that 77-39 effectively inhibited tumor growth without significant toxicity, contrasting with the higher toxicity of cisplatin.

**Conclusion:**

These findings suggest that 77–39 may serve as a novel therapeutic agent for GCTs by targeting SIRT3 and modulating the p53 and NF-κB pathways.

## 1 Introduction

Ovarian granulosa cell tumor (GCT), a major subtype of ovarian stromal tumors, derives from the sex cord-stromal cells and accounts for 2%–5% of all malignant ovarian neoplasms (Koukourakis et al., 2008; Leung et al., 2019). Unlike other types of ovarian carcinomas, GCTs can express estrogen, inhibin, and Mullerian inhibitory substance, which are also expressed by normal granulosa cells (GC) (Jamieson and Fuller, 2012; Leung et al., 2019). GCTs are commonly featured with slow and indolent disease progression and have a tendency for frequent, long delays to recurrence (Jamieson and Fuller, 2012; Kim et al., 2014). Based on the clinical manifestations and histological features, GCTs are classified into two subtypes, including juvenile granulosa cell tumors and adult granulosa cell tumors (Koukourakis et al., 2008). The C134W mutation of the FOXL2 gene has been identified in approximately 97% of adult-type GCTs. This mutation results in differential posttranslational modifications of FOXL2, contributing to the oncogenic development of GCT (Kim et al., 2014). Surgery is the main therapeutic strategy for GCT. While the disease progresses to the advanced stage, chemotherapy is the preferred choice of treatment (Koukourakis et al., 2008).

SIRT3 (sirtuin-3), an NAD^+^-dependent deacetylase, is a member of the sirtuin family residing in mitochondria and acting as a key regulator of metabolic reprogramming, DNA damage repair, and cell death in normal and tumor cells (Zhang et al., 2024). SIRT3 has been reported to play different roles in different cancer types. In epithelial ovarian cancer, SIRT3 prevents mitochondrial superoxide surges to promote cell survival by increasing the activity of the manganese superoxide dismutase (SOD2) in detached cancer cells during the metastatic process (Kim et al., 2020). Li et al. revealed that SIRT3 protects glioblastoma from ferroptosis by regulating SLC7A11 (a critical antagonist of ferroptosis) transcription through ATF4 (Li et al., 2024). However, SIRT3 serves as a tumor suppressor in certain types of cancer. In prostate cancer, SIRT3 inhibits epithelial-mesenchymal transition (EMT) and migration through attenuating the Wnt/β-catenin pathway to promote FOXO3A expression (Li et al., 2018). SIRT3 is underexpressed in hepatocellular carcinoma (HCC) and can delactylate lysine 348 lactylation of CCNE2 to prohibit HCC growth (Jin et al., 2023). This finding reveals a novel function of SIRT3 as a delactylase.

A previous study indicated that SIRT3 is highly expressed in GCT samples, and the intensity of SIRT3 staining in a GCT tissue microarray is positively correlated with the level of Ki67 (a marker of cell proliferation), which implies SIRT3 may regulate GCT growth (Schmid et al., 2021). Recently, Zhou et al. screened a selective small molecule inhibitor of SIRT3, 77-39, which displays the highest affinity for SIRT3 among several candidates (Zhou et al., 2018). The compound 77-39 is part of a bivalent binder system, where two small molecules (77 and 39) cooperatively bind to SIRT3 with a Kd of 2.14 μM. Individual components alone showed weak binding, emphasizing the importance of the bivalent system. Computational studies suggested that 77–39 binds to the cofactor-binding site of SIRT3, blocking its activity. This selective and cooperative binding mechanism makes 77-39 a potent and specific inhibitor of SIRT3 (Zhou et al., 2018). At present, researchers have not investigated targeting SIRT3 for GCT treatment. Herein, we aim to evaluate the biological effect of 77–39 on GCT cells and provide a novel approach for GCT therapy.

## 2 Materials and methods

### 2.1 Cell lines and culture

The human normal ovarian epithelial cell line IOSE-80 and human GCT-derived cell lines KGN and COV434 were used for investigation in this study. The KGN cells are derived from an adult-type GCT. The COV434 cells are derived from a juvenile-type GCT (Jamieson and Fuller, 2012). All cell lines were purchased from Shanghai iCell Bioscience Inc and cultured in Dulbecco’s modified Eagle’s medium (DMEM) (Gibco, USA) supplemented with 10% fetal bovine serum (FBS) (Gibco), 1% penicillin (Gibco), and 1% streptomycin (Gibco), at 37°C in a 5% CO_2_ humidified atmosphere.

### 2.2 Inhibitors and antibodies

The SIRT3 selective inhibitor 77-39 was provided by Professor Xiaoyu Li (The University of Hong Kong, China). The p53 inhibitor PFTα (S2929) and NF-κB inhibitor BAY 11-7082 (S2913) were purchased from Selleck (USA). Anti-p-IKKα/β (2697), anti-p65 (8242), anti-p-p65 (3033), anti-IκBα (4812), anti-p-IκBα (2859), anti-p53 (2524), anti-p21 (2947), anti-p18 (2896), anti-p16 (18769), anti-β-actin (4970), anti-mouse secondary antibody (7076) and anti-rabbit secondary antibody (7074) were purchased from Cell Signaling Technology (USA). Anti-IKKα/β (PA5-105292) antibody was purchased from Invitrogen (USA). Anti-ac-p53 (HW137) antibody was purchased from Signalway (China). Anti-SIRT3 (ab217319) antibody was from Abcam (USA).

### 2.3 SIRT3 overexpression and knockdown

SiRNA targeting SIRT3 (siSIRT3) and siRNA control (siNC) were synthesized by GenePharma Co., Ltd. (China). The indicated siRNA was transfected into KGN and COV434 cells using the RNAiMAX transfection reagent (Invitrogen). The siSIRT3 sequence was listed as follows: 5′-CCA​GCA​UGA​AAU​ACA​UUU​ATT-3’. The SIRT3 overexpression plasmid was synthesized by and purchased from GenePharma Co., Ltd. The plasmid was transfected into KGN and COV434 cells using the Lipofectamine 3000 transfection reagent (Invitrogen) according to the manufacturer’s instructions.

### 2.4 Western blot (WB) analysis

The KGN and COV434 cells were treated with the indicated treatments. Then, cells were lysed using RIPA buffer (Beyotime, China) containing phenylmethylsulfonyl fluoride (PMSF) and phosphatase inhibitor cocktail (Sigma-Aldrich, USA). The protein concentration of samples was quantified using a BCA Protein Assay Kit (Beyotime). Proteins were separated by 8%–12% sodium dodecyl sulfate-polyacrylamide (SDS-PAGE) gel electrophoresis and subsequently transferred to polyvinylidene fluoride (PVDF) membrane. After being blocked with 5% Skim milk, the membrane was incubated with primary antibodies at 4°C overnight. The membrane was then incubated with horseradish peroxidase (HRP)-conjugated secondary antibody at room temperature for 2 h. Finally, we used Clarity Western ECL Substrate (Bio-rad, USA) for imaging. The uncropped blots are available in the Supplementary Material.

### 2.5 Senescence-associated (SA) β-gal staining

Cell senescence was assessed by the detection of SA-β-gal activity using a senescence β-galactosidase staining kit (Beyotime). The cells were first fixed with the fixative solution at room temperature for 15 min. Then the cells were stained with the staining buffer at 37°C overnight. The stained cells were photographed and counted randomly from 4 to 9 fields per well under a microscope.

### 2.6 Colony formation assay

A total number of 300 KGN or 500 COV434 cells were seeded into a six-well plate. Indicated concentrations of SIRT3 inhibitor 77-39 were added to the medium. The cells were allowed to grow for 2 weeks. When the colonies were visible, cells were fixed with 4% paraformaldehyde, stained with 0.1% crystal violet, and photographed.

### 2.7 5-ethynyl-2′-deoxyuridine (EdU) assay

DNA synthesis was assessed by EdU assay using an EdU cell proliferation kit (Beyotime) according to the manufacturer’s instructions. As previously described (Zeng et al., 2024a), after treatment with 77–39, cells were incubated with the EdU staining buffer for 2.5 h and then fixed with 4% paraformaldehyde. Subsequently, the cell nuclei were stained with Hoechst and photographed under a fluorescence microscope.

### 2.8 Cell counting Kit-8 (CCK8) assay

Cell viability was evaluated by CCK8 assay. Briefly, cells were seeded at a density of 3000 cells per well in 96-well plates. Then, cells were treated with the indicated concentrations of 77–39 for 24 h. A volume of 10 μL CCK8 solution (Beyotime) was added to 100 μL culture medium and incubated with cells at 37°C for 1 h. The optical density (OD) at 450 nm was measured using a microplate reader.

### 2.9 Quantitative real-time PCR (qPCR)

TRIzol reagent (Invitrogen) was used to extract total RNA of cells, followed by the synthesis of complementary DNA (cDNA) using a PrimeScript RT reagent Kit (Takara, Japan). qPCR was performed using a TB Green Premix Ex Taq Kit (Takara) on a CFX96 real-time system (Bio-rad, USA). GAPDH was used as a reference control for normalization. The primer sequences were listed as follows: IL6 forward: 5′-AGA​CAG​CCA​CTC​ACC​TCT​TCA​G-3′ and reverse: 5′-TTC​TGC​CAG​TGC​CTC​TTT​GCT​G-3’; IL-1B forward: 5′-CCA​CAG​ACC​TTC​CAG​GAG​AAT​G-3′ and reverse: 5′-GTG​CAG​TTC​AGT​GAT​CGT​ACA​GG-3’; CXCL2 forward: 5′-GGC​AGA​AAG​CTT​GTC​TCA​ACC​C-3′ and reverse: 5′-CTC​CTT​CAG​GAA​CAG​CCA​CCA​A-3’; GAPDH forward: 5′-GGG​AAA​CTG​TGG​CGT​GAT-3′ and reverse: 5′-GAG​TGG​GTG​TCG​CTG​TTG​A-3’.

### 2.10 Co-immunoprecipitation (co-IP)

KGN and COV434 cells seeded in 10 cm culture dishes were lysed using IP lysis buffer (Beyotime, China) with protease inhibitors. Primary antibody was added to and incubated with the cleared lysate at 4°C overnight with gentle rotation. Then protein A/G beads (Invitrogen, USA) were added to and incubated with the lysate-antibody mixture at 4°C for 2 h with gentle rotation, followed by washing 3-4 times. The immunoprecipitate was eluted with the addition of elution buffer. The immunoprecipitated proteins were subsequently subjected to WB analysis.

### 2.11 RNA sequencing

Transcriptome sequencing and differential expression analysis were performed by Novogene Inc. (Tianjin, China). As previously reported (Zeng et al., 2024b), the total RNA of 77–39 treated and non-treated KGN or Cov434 cells was extracted with TRIzol reagent. Total amounts and integrity of RNA were assessed using the RNA Nano 6000 Assay Kit of the Bioanalyzer 2100 system (Agilent Technologies, USA). When the libraries were constructed and qualified, they were subsequently subjected to RNA sequencing with Illumina NovaSeq 6000.

### 2.12 Gene set enrichment analysis (GSEA)

GSEA was performed using GSEA software (https://www.gsea-msigdb.org/gsea/index.jsp) as previously described (Zeng et al., 2024a). The RNA sequencing expression profiles of 77–39 non-treated group and treated group were imported into the GSEA software. When the false discovery rate (FDR) was <0.25 and the nominal p-value was <0.05, the enriched gene sets were considered statistically significant.

### 2.13 Animal experiments

Four-week-old female BALB/c nude mice (six mice per group) were purchased from Charles River Laboratories and maintained under specific pathogen-free conditions. KGN cells (1 × 10^6^ cells in the mixture of 0.1 mL PBS and 0.1 mL Matrigel (Corning, USA) per mouse) were injected subcutaneously into the right dorsal flank (designated as day 1). Fourteen days after injection, mice were randomly assigned to 5 groups (6 mice per group). 77-39 was dissolved in normal saline. One hundred microliters of indicated concentrations of 77–39 were administered by gavage every day. Cisplatin (CDDP) was injected intraperitoneally into mice every other day. Tumor volume was measured every other day, and tumor volume was calculated as volume (mm^3^) = length × width^2^. Mice were euthanized on day 31. Tumors were isolated and harvested from indicated mice and subsequently were fixed, embedded with paraffin, and examined by hematoxylin and eosin (H&E), SA-β-gal, and immunohistochemical (IHC) staining. All experimental procedures and protocols were reviewed and approved by Shenzhen TopBiotech Co., Ltd. Animal Center (Shenzhen, China). The H-score scoring system was employed to assess the differences in IHC staining, with the scoring formula defined as H-score = ∑ (percentage of positive cells × intensity). The percentage of positive cells was expressed as a fraction of 100 (e.g., 20% = 20). The intensity of staining was graded on a scale from 0 to 3, with 0 representing no staining, 1 representing weak staining, 2 representing moderate staining, and 3 representing strong staining.

### 2.14 Statistical analysis

Data shown in this study were representative of the statistics [mean value ±standard deviation (SD)] of the results from at least three independent experiments. The students’ two-tailed t-test was used for all statistical analyses, with the level of significance set at (***) p < 0.005, (**) p < 0.01, and (*) p < 0.05. All the statistical analyses were conducted by GraphPad Prism 7 software.

## 3 Results

### 3.1 Inhibition of SIRT3 by 77–39 suppresses GCT cell growth and induces cellular senescence

**FIGURE 1:**
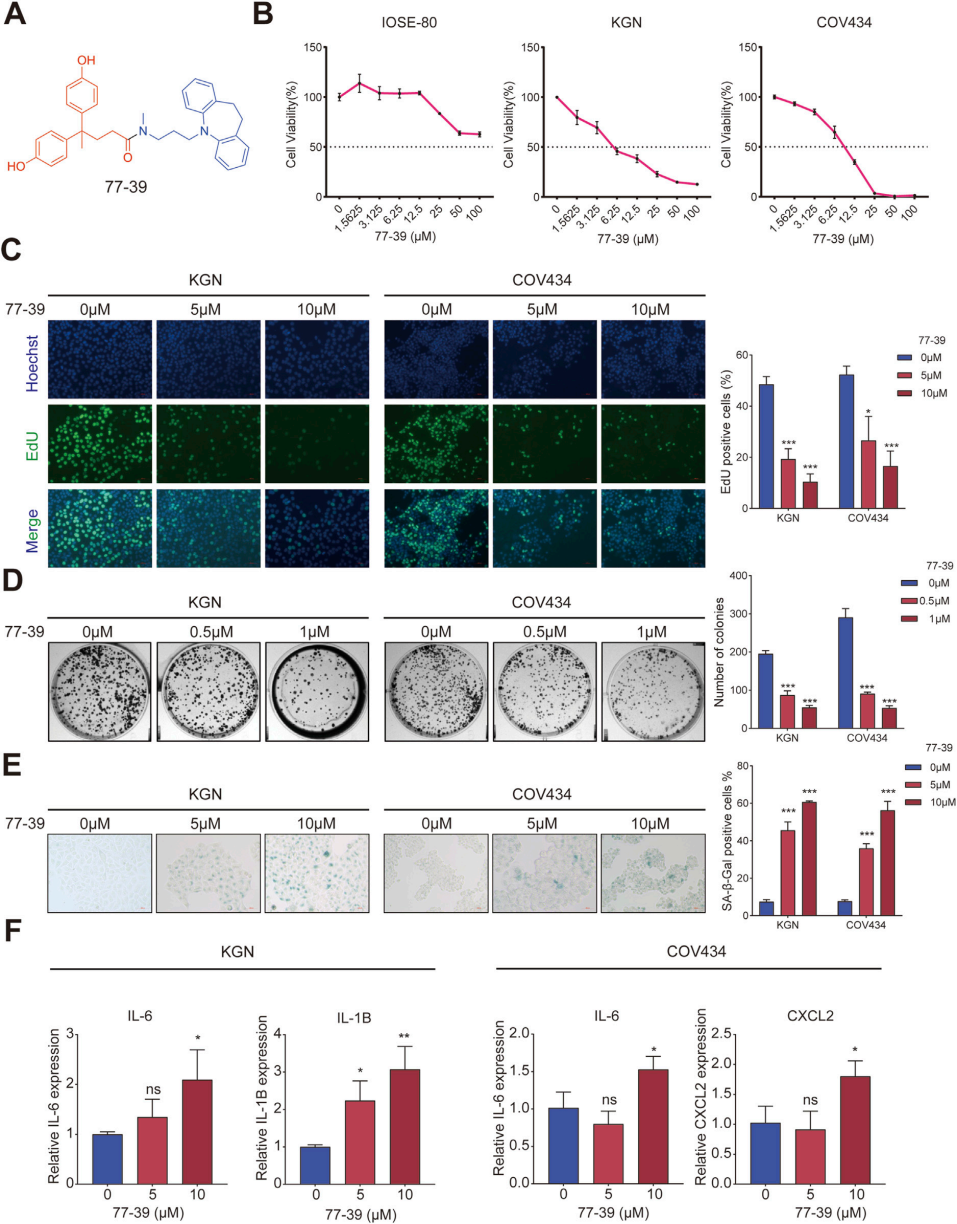
77–39 suppresses GCT cell growth and induces cellular senescence. **(A)** Chemical structure of 77–39. **(B)** CCK8 assay was used to evaluate the effects of 77–39 on the cell viability of IOSE-80, KGN, and COV434. **(C,D)** EdU assay and colony formation assay were used to evaluate the effects of 77–39 on the proliferative activity of KGN and COV434. **(E)** SA-β-gal staining was used to evaluate the effects of 77–39 on the cellular senescence of KGN and COV434. **(F)** qPCR experiment was performed to examine the changes in mRNA expression levels of IL-6, IL-1B, and CXCL2 after treatment with the indicated concentrations of compound 77-39.

The chemical structural formula of 77–39 is shown in Figure 1A. To evaluate the effect of 77–39 on GCT cells, we first examined the cell viability of normal ovarian epithelial cell line IOSE-80 and two GCT cell lines KGN and CO434 with the treatment of indicated 77-39 concentration. The results showed that 77–39 significantly suppressed the viability of KGN and COV434 cells in a concentration-dependent manner, while it did not inhibit the normal cell line IOSE-80 as effectively (Figure 1B). We further performed EdU assay and colony formation assay to assess the impact of 77–39 on GCT cell proliferation. As shown in Figures 1C,D, the proportion of EdU-positive cells and the number of colonies were significantly higher in the control group, which were remarkably reduced in a concentration-dependent manner in the presence of 77–39, indicating that 77-39 inhibited the proliferative activity of GCT cells. As SIRT3 is a protein related to aging and senescence (Zhou et al., 2022), we then tested whether 77-39 induces GCT senescence. By SA-β-gal staining assay, we found that 77–39 significantly induced cellular senescence compared with the control group (Figure 1E). Cellular senescence results in the secretion of a wide range of cytokines, chemokines, growth factors, and proteases/inhibitors, collectively known as the senescence-associated secretory phenotype (SASP) (Lasry and Ben-Neriah, 2015). We detected several SASP-associated proteins, such as IL-6, IL-1B, and CXCL2, by qPCR. As expected, treatment with 77-39 significantly elevated the mRNA expression levels of all cytokines (Figure 1F). In summary, the results demonstrate that 77-39 inhibits GCT cell growth by inducing senescence.

### 3.2 77–39 exerts its function through the p53 and NF-κB signaling pathways

**FIGURE 2:**
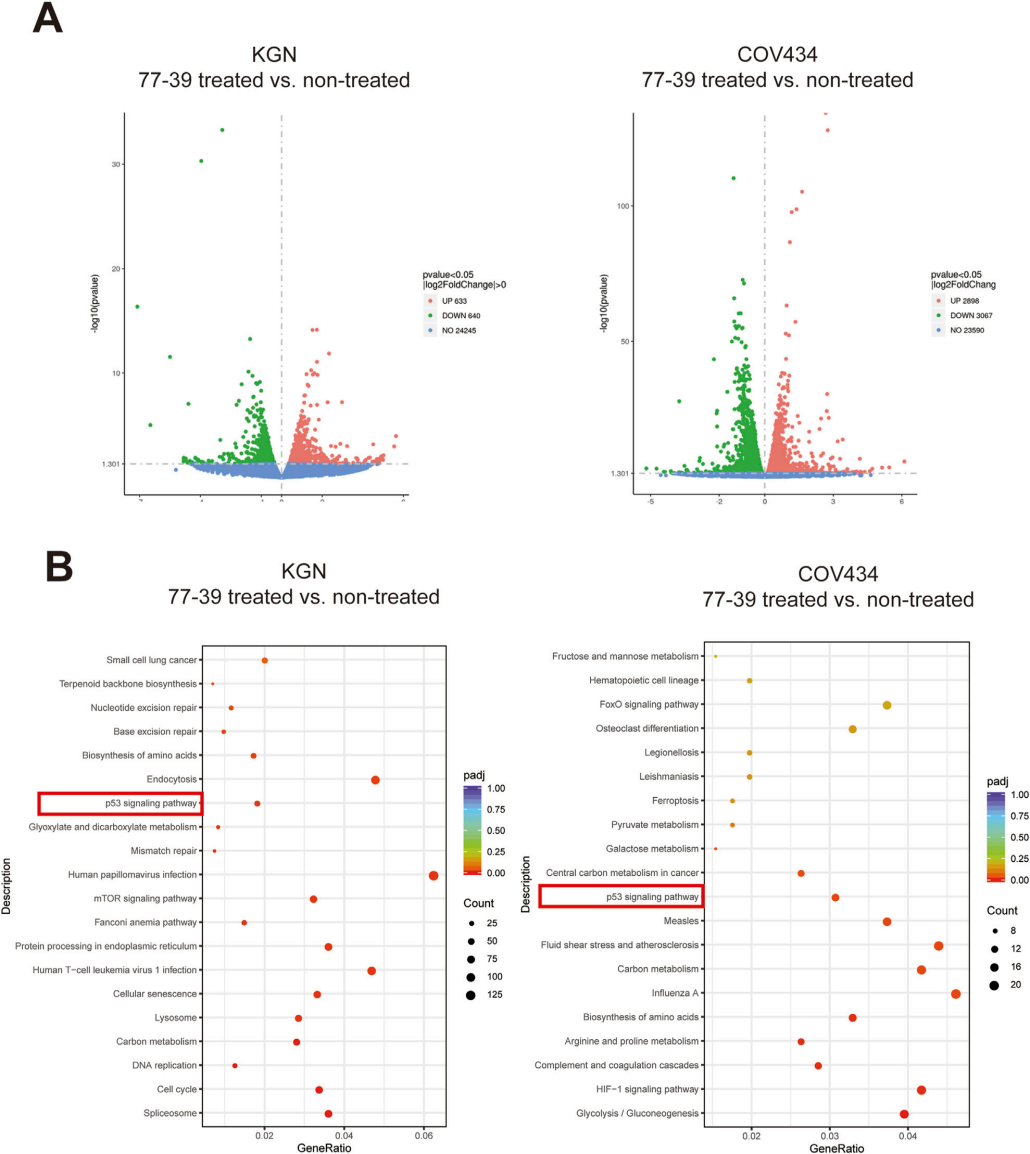
Transcriptomic sequencing and KEGG enrichment analysis of 77–39 treated and non-treated KGN and COV434 cells. **(A)** Volcano plots showing DEGs in KGN and COV434 cells treated with 77–39 compared to untreated controls. Genes with a p-value <0.05 and a log2|fold change| > 0 are considered DEGs. **(B)** KEGG enrichment analysis using DEGs in KGN and COV434 cells treated with 77–39 compared to untreated controls.

**FIGURE 3:**
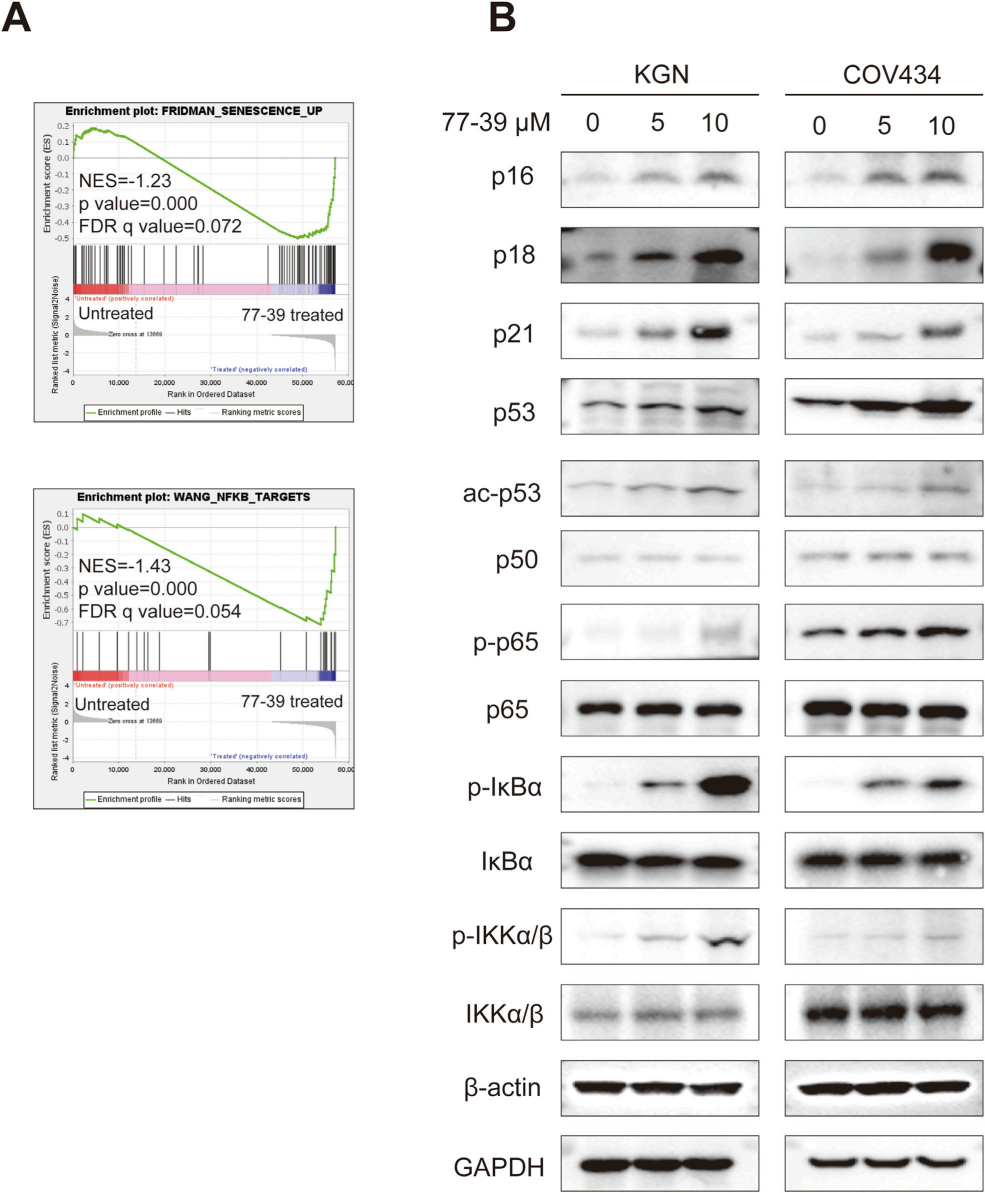
77-39 exerts its function through the p53 and NF-κB signaling pathways. **(A)** GSEA analysis showing senescence and NF-κB-related gene sets were enriched in the 77-39 treated group compared to the untreated group. **(B)** WB analyses showing that cell cycle-related proteins, p53, and NF-κB signaling were activated in a dose-dependent manner after treatment with 77–39.

To elucidate the potential mechanisms by which 77-39 inhibits the proliferation of GCT cells, we performed RNA sequencing on KGN and COV434 cells treated with or without 77-39. As summarized in Figure 2A, following treatment of 77–39, 2898 genes were significantly upregulated while 3067 genes were significantly downregulated in COV434 cells, 633 genes were significantly upregulated while 640 genes were significantly downregulated in KGN cells. Subsequently, we performed Kyoto Encyclopedia of Genes and Genomes (KEGG) enrichment analysis using the differentially expressed genes (DEGs), and we noticed that the term “p53 signaling pathway” was significantly enriched in both COV434 and KGN cells (Figure 2B), indicating that 77–39 might regulate p53 pathway in GCT. Indeed, p53 has been reported to be a downstream factor of SIRT3, and deacetylated and suppressed by SIRT3 (Li et al., 2010; Chen et al., 2017; Xiong et al., 2018). We further performed GSEA and found that gene sets including “FRIDMAN_SENESCENCE_UP” and “WANG_NFKB_TARGETS” were significantly enriched in the 77-39 treated group, indicating 77–39 might activate the NF-κB pathway and induce senescence (Figure 3A). By WB analyses, we found that, following treatment of 73–39, the protein expression of cell cycle-related proteins like p16, p18, p21, p53, and acetylated p53 were elevated in a dose-dependent manner. The key proteins in the NF-κB pathway, such as p65, IκBα, and IKKα/β, were also activated (Figure 3B). These results indicate that 77-39 inhibited cell growth and induced senescence through the p53 and NF-κB signaling in GCT cells.

### 3.3 Silencing SIRT3 in GCT cells has the same effect as treating with 77–39

**FIGURE 4:**
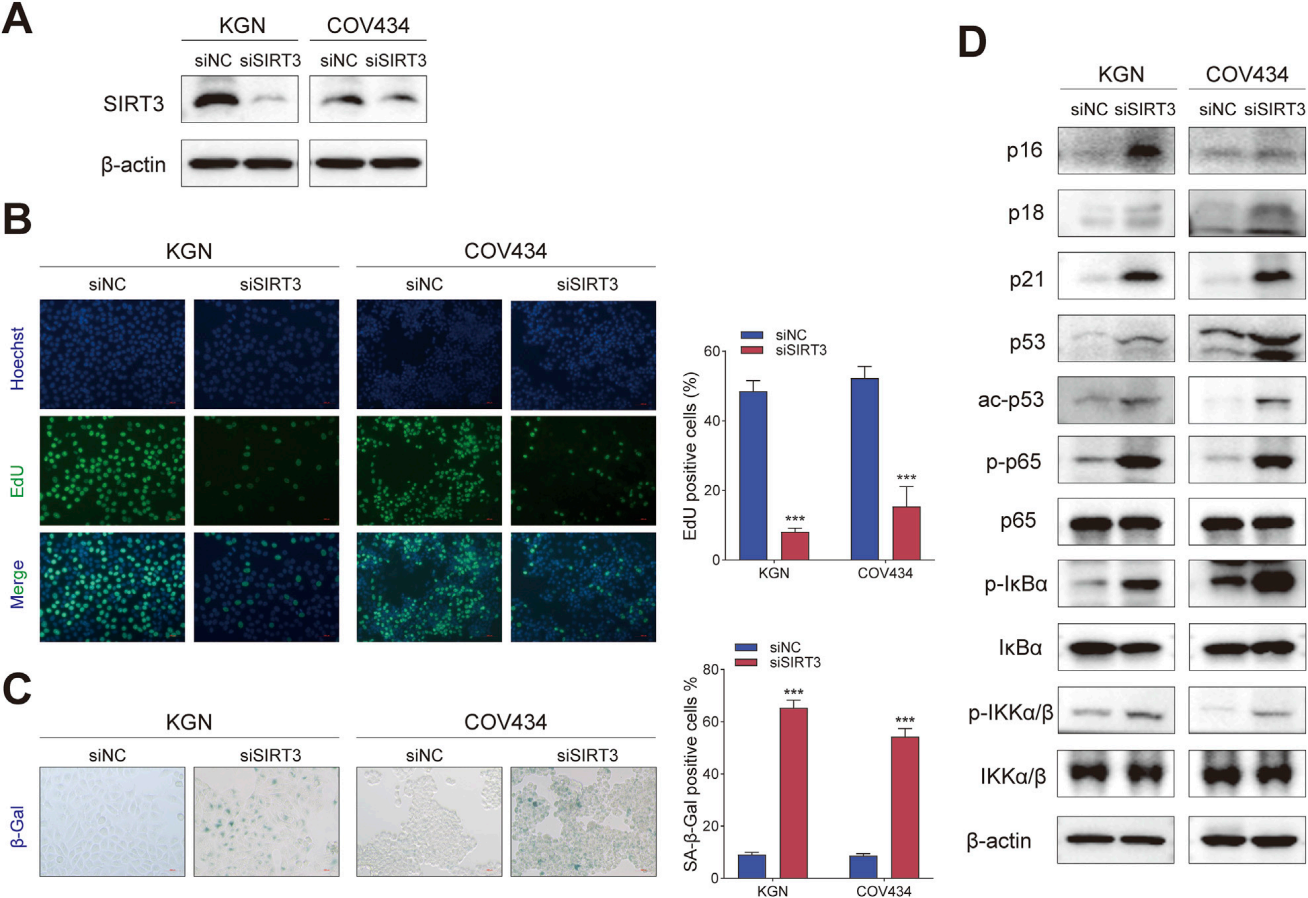
Silencing SIRT3 in GCT cells has the same effect as treating with 77–39. **(A)** WB analyses showing the knockdown efficacy of SIRT3 siRNA in KGN and COV434 cells. **(B)** EdU assay showing the effects of SIRT3 silence on cell proliferation in KGN and COV434 cells. **(C)** SA-β-gal staining was used to evaluate the effects of the silence of SIRT3 on the cellular senescence of KGN and COV434. **(D)** WB analyses showing that cell cycle-related proteins, p53, and NF-κB signaling were activated after silencing SIRT3 in KGN and COV434 cells.

To verify that 77-39 functioned through targeting SIRT3, we knocked down the expression of SIRT3 in KGN and COV434 cells by siRNA (Figure 4A). By EdU assay and SA-β-gal staining, we found that the silence of SIRT3 significantly inhibited cell proliferation and promoted senescence in GCT cells (Figures 4B,C). The protein expression of cell cycle-related proteins and the phosphorylation levels of key proteins in the NF-κB pathway also underwent significant changes (Figure 4D), which were consistent with the results observed upon treatment with 77–39.

**FIGURE 5:**
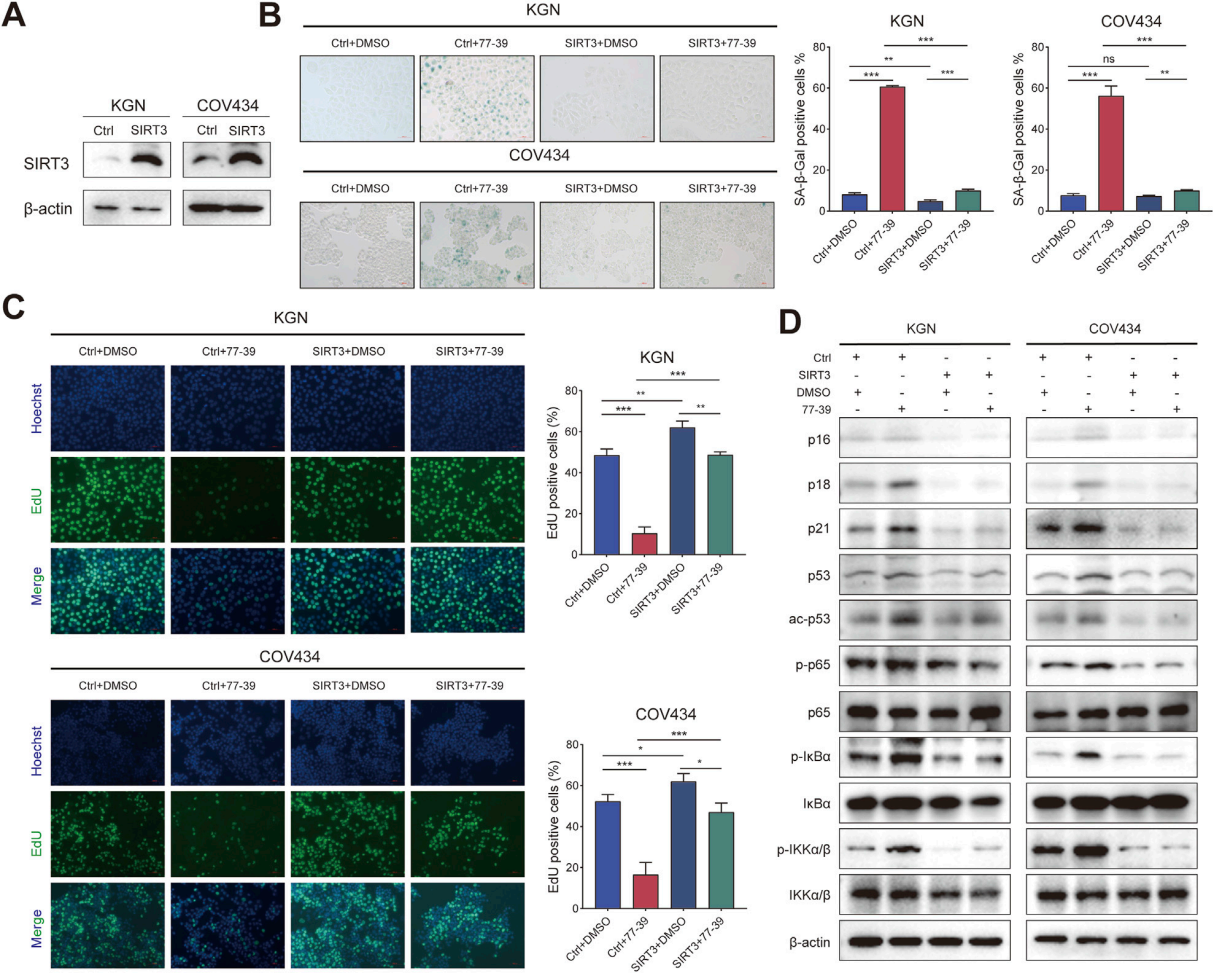
Overexpressing SIRT3 in GCT cells reverses the inhibitory effect of 77–39 in GCT cells. **(A)** WB analyses showing the successful overexpression of SIRT3 in KGN and COV434 cells. **(B)** SA-β-gal staining was used to evaluate the effects of overexpression of SIRT3 on the cellular senescence of KGN and COV434. **(C)** EdU assay showing the effects of SIRT3 overexpression on cell proliferation in KGN and COV434 cells. **(D)** WB analyses showing that cell cycle-related proteins, p53, and NF-κB signaling were inactivated after overexpressing SIRT3 in KGN and COV434 cells.

Correspondingly, we overexpressed SIRT3 in KGN and COV434 cells (Figure 5A). The inhibitory effect of 77–39 on cell proliferation and senescence in GCT cells was reversed by overexpression of SIRT3 (Figures 5B,C). The expression of proteins related to the p53 and NF-κB pathways was also suppressed by the overexpression of SIRT3 (Figure 5D). Overall, the above results indicated that SIRT3 is the target through which 77-39 exerts its effects.

### 3.4 Inhibition of p53 or NF-κB counteracts the effect of 77–39 in GCT cells

**FIGURE 6:**
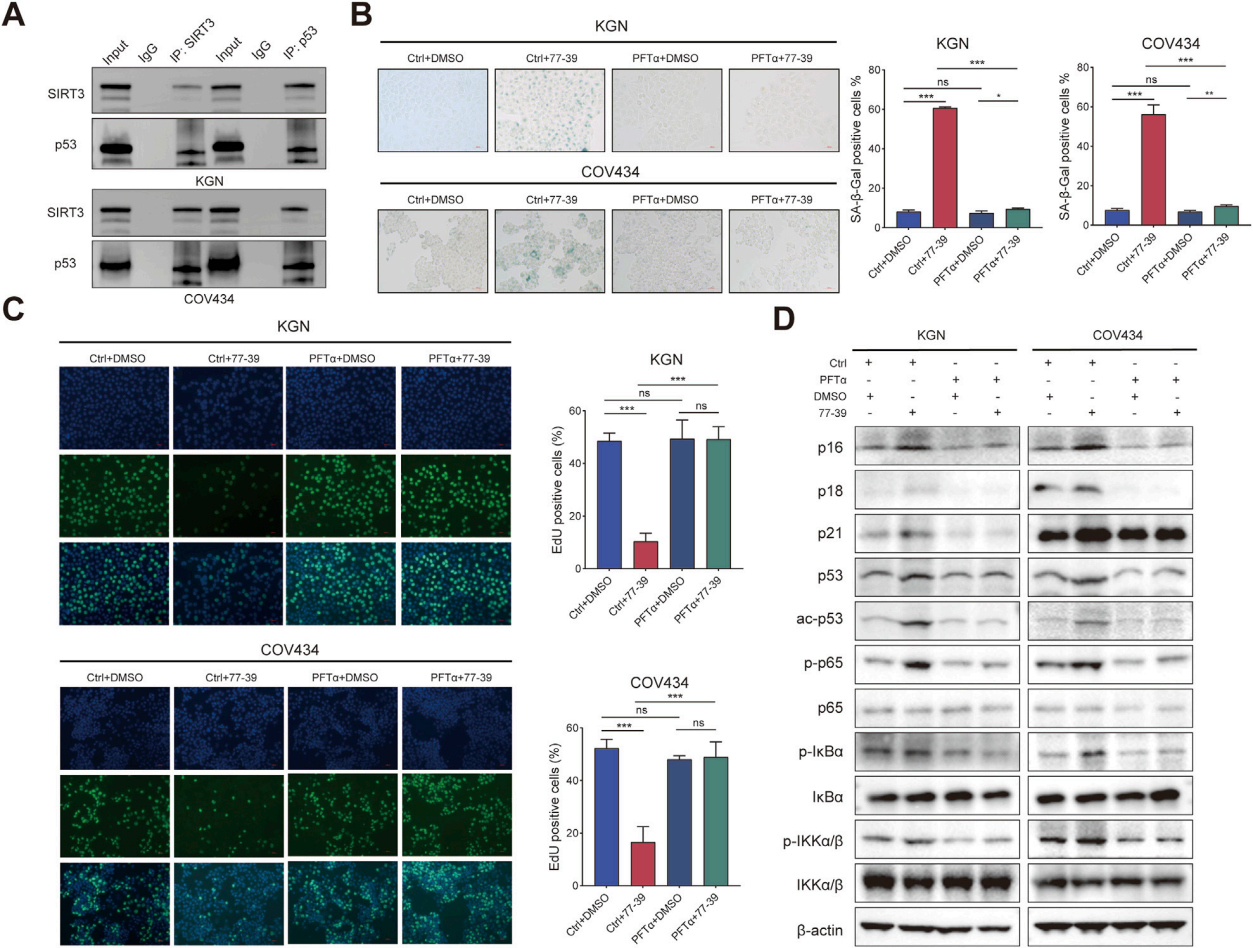
Inhibition of p53 counteracts the effect of 77–39 in GCT cells. **(A)** co-IP and WB analyses showing the interaction between SIRT3 and p53 in KGN and COV434 cells. **(B)** SA-β-gal staining was used to evaluate the effects of inhibition of p53 on the cellular senescence of KGN and COV434. **(C)** EdU assay showing the effects of p53 inhibition on cell proliferation in KGN and COV434 cells. **(D)** WB analyses showing that cell cycle-related proteins, p53, and NF-κB signaling were downregulated after p53 inhibition in KGN and COV434 cells.

To verify that p53 is a downstream effector molecule of SIRT3, we performed co-IP experiments and found that p53 interacted with SIRT3 in both KGN and COV434 cells (Figure 6A). To determine whether p53 mediated the inhibitory effect of 77–39 on GCT cells, we inhibited p53 using the inhibitor PFTα. The results showed that inhibiting p53 led to a significant recovery in cell proliferation and a reduced rate of senescence in GCT cells treated with 77–39 (Figures 6B,C). The proteins that inhibited the cell cycle process were also downregulated (Figure 6D). Intriguingly, we found that the inhibition of p53 significantly affected the phosphorylated level of NF-κB-related proteins, which indicated that p53 might regulate NF-κB signaling in GCT (Figure 6D).

**FIGURE 7:**
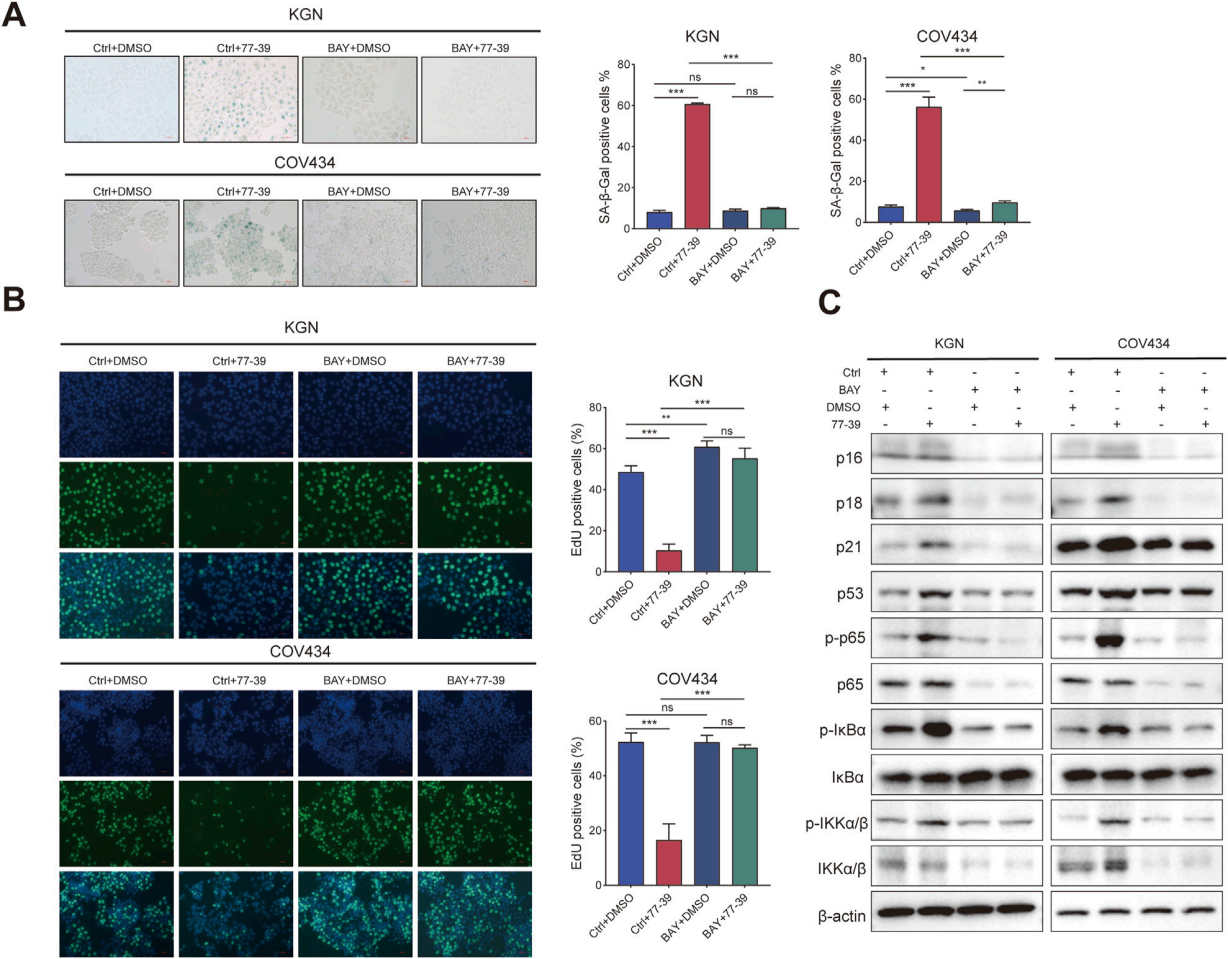
Inhibition of NF-κB counteracts the effect of 77–39 in GCT cells. **(A)** SA-β-gal staining was used to evaluate the effects of inhibition of NF-κB on the cellular senescence of KGN and COV434. **(B)** EdU assay showing the effects of NF-κB inhibition on cell proliferation in KGN and COV434 cells. **(C)** WB analyses showing that cell cycle-related proteins, p53, and NF-κB signaling were downregulated after NF-κB inhibition in KGN and COV434 cells.

The NF-κB pathway was reported to be activated in senescent cells and implicated in regulating the expression of the inflammatory factors (Chen et al., 2018; Wang et al., 2022). Similarly, we also inhibited the activity of NF-κB in GCT cells using the inhibitor BAY 11-7082. The proliferative capacity of GCT cells and the expression of related proteins in GCT cells treated with 77–39 were restored upon inhibition of NF-κB. (Figures 7A–C). In summary, these results demonstrated that p53 and NF-κB are downstream effectors of SIRT3.

### 3.5 77–39 suppresses GCT tumorigenicity *in vivo*

**FIGURE 8:**
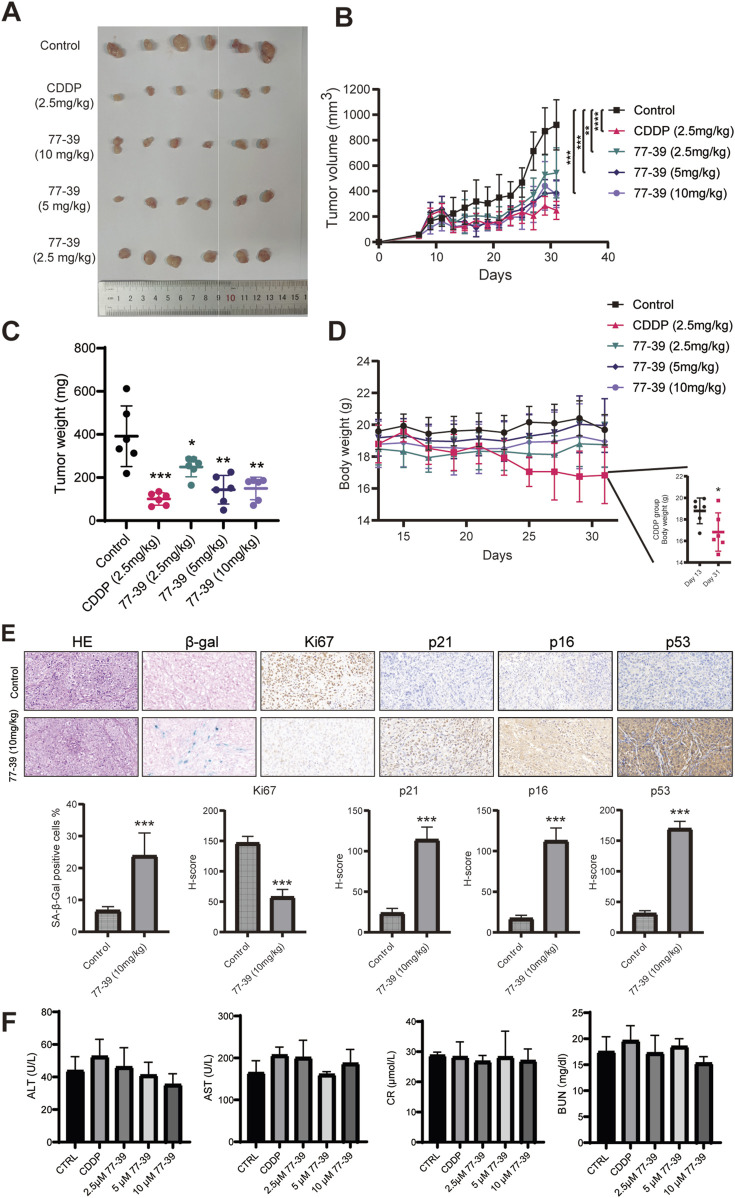
77–39 suppresses GCT tumorigenicity *in vivo*. **(A)** Image of the subcutaneous KGN xenografts from mice that received indicated treatment. **(B)** Growth curves of KGN xenografts measured by a digital caliper. **(C)** Weight of KGN xenografts. **(D)** Body weight of mice that received indicated treatment. **(E)** KGN xenografts from the control and 77-39 (10 mg/kg) treated groups were subjected to HE staining, SA-β-gal staining, and IHC staining for Ki67, p21, p16, and p53. Histogram showing the statistical results and differences of the proportion of SA-β-gal positive cells and H-score. **(F)** Measurement of liver and kidney function markers in different treatment groups.

To evaluate the efficacy and safety of 77–39 in inhibiting the tumorigenicity of GCT *in vivo*, we established a subcutaneous tumor model in nude mice. KGN cells were injected subcutaneously into the right dorsal flank of nude mice. Fourteen days after injection, mice were randomly assigned to 5 groups (6 mice per group). One hundred microliters of indicated concentrations of 77–39 were administered by gavage every day. CDDP, a chemotherapeutic agent used clinically for the treatment of GCT, was injected intraperitoneally into mice every other day. The results showed that both CDDP and 77-39 could effectively inhibit the tumor growth of GCT *in vivo* (Figures 8A–C). CDDP demonstrated the best tumor-inhibitory capacity (Figure 8C). However, the body weight of mice in the CDDP-treated group significantly decreased (Figure 8D), while the 77-39-treated groups did not show significant changes in body weight, indicating that, compared with CDDP, 77-39 exhibited lower drug toxicity. In addition, xenograft tumors excised from nude mice were further subjected to HE staining, SA-β-gal staining, and IHC staining. Consistent with our *in vitro* experimental results, 77-39 was capable of inducing tumor senescence, inhibiting tumor proliferation, and regulating the expression of cell cycle-related proteins (Figure 8E). At the endpoint of the animal experiment, we collected blood from the nude mice and measured the indicators of liver and kidney function. The results showed that compound 77–39 did not exhibit significant toxicity to the liver and kidneys, indicating its safety profile (Figure 8F).

## 4 Discussion

GCT is the most common non-epithelial malignancy of the ovary. However, it remains a rare disease, and there is a scarcity of high-level evidence to guide its management, especially in the advanced stage (Salkeni et al., 2024). Oophorectomy is currently a primary treatment method for primary GCT (Brown et al., 2009; Zhao et al., 2019). It is estimated that approximately 20% of adult GCTs will recur, with a median time to recurrence of 5 years from the initial diagnosis (Evans et al., 1980; Malmstrom et al., 1994). When GCT progresses to an inoperable or metastatic disease, several palliative treatment options are available, including conventional cytotoxic chemotherapy, endocrine therapy, radiation therapy, and targeted therapies (Salkeni et al., 2024). Developing novel therapeutic approaches is of paramount importance for patients with advanced GCT.

SIRT3 protein level was found to be upregulated in ovarian cancer tissues (Signorile et al., 2019). IDO1^high^ ovarian cancer cell-derived extracellular vesicles upregulated SIRT3 expression in endothelial cells by increasing acetylation modification. The endothelial SIRT3-mediated mitophagy, in turn, promoted angiogenesis in ovarian cancer (Ying et al., 2024). However, SIRT3 has also been shown to be a tumor suppressor in ovarian epithelial cancer. A prior investigation revealed that the expression of the SIRT3 protein was markedly reduced in ovarian cancer tissues and in the highly metastatic HO-8910PM cell line (Dong et al., 2016; Shi et al., 2020). Activation of SIRT3 in ovarian epithelial cancer induced apoptosis, decreased mitochondrial membrane potential, and promoted the fission process of mitochondria (Hou et al., 2019). Metformin, an inhibitor of mitochondrial complex 1, could induce energy stress and apoptosis in ovarian epithelial cancer cells. Interestingly, the activation of SIRT3, which is enhanced by Metformin, further aggravates these effects (Wu et al., 2018).

Despite the controversial role of SIRT3 in ovarian epithelial cancer, its function in polycystic ovary syndrome (PCOS)—an ovarian granulosa cell-associated disease—is well-defined. SIRT3 could enhance ovarian morphology and serum sex hormone levels in mice with PCOS induced by dihydrotestosterone (DHT). It also inhibited apoptosis both *in vitro* and *in vivo*. Additionally, SIRT3 inhibited mitochondrial reactive oxygen species (ROS) production to ameliorate mitochondrial dysfunction in DHT-induced KGN cells through the FOXO1/PGC-1α signaling pathway (Pang et al., 2023). Moreover, SIRT3 knockdown in granulosa cells led to mitochondrial dysfunction, increased oxidative stress, and impaired glucose metabolism. These alterations could potentially contribute to the development of compromised oocyte quality in PCOS (Fu et al., 2014; Zhang et al., 2022). It is also demonstrated that SIRT3 inactivation or knockdown could elevate ROS production, leading to embryonic development arrest and reduced proliferative activity of Leydig cells (Kawamura et al., 2010; Matoba et al., 2024). The above evidence suggested that SIRT3 exerted a protective role in granulosa cells.

In GCT, although a previous study showed that the knockdown of SIRT3 in KGN cells impaired their proliferative ability (Schmid et al., 2021), the underlying molecular mechanism by which SIRT3 regulates cell growth remains underexplored. In this study, we evaluated the therapeutic effects of 77–39, a novel SIRT3-selective inhibitor developed by Zhou et al., on GCT. Through clonogenic assays, EdU incorporation assays, and SA-β-gal staining, we found that compound 77–39 significantly inhibited the proliferation of GCT cells and induced cellular senescence, with an increase in the expression of SASP-related proteins (e.g., IL-6, IL-1B, CXCL2). Through RNA sequencing and subsequent KEGG and GSEA enrichment analyses, we found that pathways related to p53, NF-κB, and senescence were activated following treatment with 77–39. The results were further confirmed by WB experiments. Previously, Zhu et al. demonstrated that SIRT3 expression showed age-dependent decreases in the ovary, and depletion of SIRT3 in mice by CRISPR/Cas-9 accelerated ovarian aging, as evidenced by reduced offspring sizes, decreased follicle reserves, and lower levels of oocyte markers (Bmp15 and Gdf9), along with increased expression of genes related to aging and inflammation (p16, p21, Il-1α, and Il-1β). This study provided additional evidence to support our results. Our work, for the first time, has elucidated the oncogenic mechanisms of SIRT3 in GCT by using a SIRT3 selective inhibitor.

Next, to further validate that the p53 and NF-κB pathways mediated the inhibitory effects of 77–39 on GCT, we inhibited p53 and NF-κB, respectively. As expected, inhibiting p53 and NF-κB could reverse the inhibitory effects of 77–39 on GCT cells, demonstrating they were downstream effector of SIRT3. Unexpectedly, we found that p53 and NF-κB signaling pathways might interactively regulate each other in GCT. According to previous studies, p53 could activate NF-κB, and this activation is crucial for p53-mediated apoptosis (Ryan et al., 2000; Yin and Yu, 2018; Tan et al., 2022). Inhibition of NF-κB by BAY 11-7082 reduced the protein expression of p53 (Han et al., 2024). These findings may broaden our understanding of the crosstalk between p53 and NF-κB. Additionally, the xenograft tumor model was used to evaluate the efficacy of 77–39 in inhibiting tumor growth *in vivo*. Compound 77–39 demonstrated significant tumor-suppressive activity in in vivo experiments. Regarding the safety of 77–39, *in vitro* experiments revealed that compared with GCT cells, 77-39 exhibited weaker inhibitory activity against the normal ovarian epithelial cell line IOSE-80. In in vivo experiments using nude mice, the administration of 77–39 did not affect the body weight of the mice. Moreover, compared with the control group, 77–39 did not exhibit significant toxicity to the liver and kidneys, thereby emphasizing its safety profile. Although cisplatin remains the most commonly used therapeutic agent for advanced GCT and has demonstrated the strongest inhibitory efficacy in xenograft tumor models, our experiments indicated that 77–39 has superior safety compared with cisplatin. Overall, these works suggested that 77-39 could serve as a potential therapeutic agent for GCT.

Our study has several limitations. First, we only used immortalized cell lines for *in vitro* and *in vivo* experiments, which may not fully reflect the clinical therapeutic effects. Due to the scarcity of clinical samples of GCT, we were unable to evaluate the therapeutic efficacy of 77–39 by constructing patient-derived organoids and patient-derived xenograft models, both of which could better simulate the clinical therapeutic effects of 77–39 on GCT. Additionally, our experimental results indicated that treatment with 77–39, knockdown, or overexpression of SIRT3 could affect the p53 and NF-κB signaling pathways. It is known that SIRT3 can deacetylate p53 to regulate its stability. However, the mechanism by which SIRT3 affects the NF-κB signaling pathway remains to be elucidated. Moreover, our results showed that Inhibition of p53 using PFTα affected the NF-κB signaling, while inhibition of NF-κB using BAY 11-7082 affected the p53 signaling, indicating an interacting network between p53 and NF-κB signaling, which our study has not yet adequately explained. In conclusion, our findings propose a novel perspective and therapeutic option for GCT that primarily targets SIRT3. Future research should focus on elucidating the mechanisms underlying the interaction between SIRT3, the p53 signaling pathway, and the NF-κB signaling pathway and exploring the therapeutic potential of 77–39 in more clinically relevant models.

## Data Availability

The raw data of transcriptomic sequencing has been submitted to the GEO database with accession number GSE293585.

## References

[B1] Brown J. Sood A. K. Deavers M. T. Milojevic L. Gershenson D. M. (2009). Patterns of metastasis in sex cord-stromal tumors of the ovary: can routine staging lymphadenectomy be omitted? Gynecol. Oncol. 113 (1), 86–90. 10.1016/j.ygyno.2008.12.007 19162310

[B2] Chen F. Long Q. Fu D. Zhu D. Ji Y. Han L. (2018). Targeting SPINK1 in the damaged tumour microenvironment alleviates therapeutic resistance. Nat. Commun. 9 (1), 4315. 10.1038/s41467-018-06860-4 30333494 PMC6193001

[B3] Chen J. Wang A. Chen Q. (2017). SirT3 and p53 deacetylation in aging and cancer. J. Cell Physiol. 232 (9), 2308–2311. 10.1002/jcp.25669 27791271

[B4] Dong X. C. Jing L. M. Wang W. X. Gao Y. X. (2016). Down-regulation of SIRT3 promotes ovarian carcinoma metastasis. Biochem. Biophys. Res. Commun. 475 (3), 245–250. 10.1016/j.bbrc.2016.05.098 27216459

[B5] Evans A. T. Gaffey T. A. Malkasian G. D. Jr. Annegers J. F. (1980). Clinicopathologic review of 118 granulosa and 82 theca cell tumors. Obstet. Gynecol. 55 (2), 231–238. 6243409

[B6] Fu H. Wada-Hiraike O. Hirano M. Kawamura Y. Sakurabashi A. Shirane A. (2014). SIRT3 positively regulates the expression of folliculogenesis- and luteinization-related genes and progesterone secretion by manipulating oxidative stress in human luteinized granulosa cells. Endocrinology 155 (8), 3079–3087. 10.1210/en.2014-1025 24877629

[B7] Han J. Zhan L. N. Huang Y. Guo S. Zhou X. Kapilevich L. (2024). Moderate mechanical stress suppresses chondrocyte ferroptosis in osteoarthritis by regulating NF-κB p65/GPX4 signaling pathway. Sci. Rep. 14 (1), 5078. 10.1038/s41598-024-55629-x 38429394 PMC10907644

[B8] Hou L. Wang R. Wei H. Li S. Liu L. Lu X. (2019). ABT737 enhances ovarian cancer cells sensitivity to cisplatin through regulation of mitochondrial fission via Sirt3 activation. Life Sci. 232, 116561. 10.1016/j.lfs.2019.116561 31247208

[B9] Jamieson S. Fuller P. J. (2012). Molecular pathogenesis of granulosa cell tumors of the ovary. Endocr. Rev. 33 (1), 109–144. 10.1210/er.2011-0014 22240241

[B10] Jin J. Bai L. Wang D. Ding W. Cao Z. Yan P. (2023). SIRT3-dependent delactylation of cyclin E2 prevents hepatocellular carcinoma growth. EMBO Rep. 24 (5), e56052. 10.15252/embr.202256052 36896611 PMC10157311

[B11] Kawamura Y. Uchijima Y. Horike N. Tonami K. Nishiyama K. Amano T. (2010). Sirt3 protects in vitro-fertilized mouse preimplantation embryos against oxidative stress-induced p53-mediated developmental arrest. J. Clin. Invest 120 (8), 2817–2828. 10.1172/JCI42020 20644252 PMC2912189

[B12] Kim J. H. Kim Y. H. Kim H. M. Park H. O. Ha N. C. Kim T. H. (2014). FOXL2 posttranslational modifications mediated by GSK3β determine the growth of granulosa cell tumours. Nat. Commun. 5, 2936. 10.1038/ncomms3936 24390485

[B13] Kim Y. S. Gupta Vallur P. Jones V. M. Worley B. L. Shimko S. Shin D. H. (2020). Context-dependent activation of SIRT3 is necessary for anchorage-independent survival and metastasis of ovarian cancer cells. Oncogene 39 (8), 1619–1633. 10.1038/s41388-019-1097-7 31723239 PMC7036012

[B14] Koukourakis G. V. Kouloulias V. E. Koukourakis M. J. Zacharias G. A. Papadimitriou C. Mystakidou K. (2008). Granulosa cell tumor of the ovary: tumor review. Integr. Cancer Ther. 7 (3), 204–215. 10.1177/1534735408322845 18815151

[B15] Lasry A. Ben-Neriah Y. (2015). Senescence-associated inflammatory responses: aging and cancer perspectives. Trends Immunol. 36 (4), 217–228. 10.1016/j.it.2015.02.009 25801910

[B16] Leung D. T. H. Nguyen T. Oliver E. M. Matti J. Alexiadis M. Silke J. (2019). Combined PPARγ activation and XIAP inhibition as a potential therapeutic strategy for ovarian granulosa cell tumors. Mol. Cancer Ther. 18 (2), 364–375. 10.1158/1535-7163.MCT-18-0078 30530769

[B17] Li R. Quan Y. Xia W. (2018). SIRT3 inhibits prostate cancer metastasis through regulation of FOXO3A by suppressing Wnt/β-catenin pathway. Exp. Cell Res. 364 (2), 143–151. 10.1016/j.yexcr.2018.01.036 29421536

[B18] Li S. Banck M. Mujtaba S. Zhou M. M. Sugrue M. M. Walsh M. J. (2010). p53-induced growth arrest is regulated by the mitochondrial SirT3 deacetylase. PLoS One 5 (5), e10486. 10.1371/journal.pone.0010486 20463968 PMC2864751

[B19] Li X. Zhang W. Xing Z. Hu S. Zhang G. Wang T. (2024). Targeting SIRT3 sensitizes glioblastoma to ferroptosis by promoting mitophagy and inhibiting SLC7A11. Cell Death Dis. 15 (2), 168. 10.1038/s41419-024-06558-0 38395990 PMC10891132

[B20] Malmstrom H. Hogberg T. Risberg B. Simonsen E. (1994). Granulosa cell tumors of the ovary: prognostic factors and outcome. Gynecol. Oncol. 52 (1), 50–55. 10.1006/gyno.1994.1010 8307501

[B21] Matoba H. Fujii C. Maruyama K. Kawakubo M. Momose M. Sano K. (2024). Sirt3 regulates proliferation and progesterone production in leydig cells via suppression of reactive oxygen species. Endocrinology 165 (4), bqae017. 10.1210/endocr/bqae017 38354290

[B22] Pang X. Cheng J. Wu T. Sun L. (2023). SIRT3 ameliorates polycystic ovary syndrome through FOXO1/PGC-1α signaling pathway. Endocrine 80 (1), 201–211. 10.1007/s12020-022-03262-x 36598711

[B23] Ryan K. M. Ernst M. K. Rice N. R. Vousden K. H. (2000). Role of NF-kappaB in p53-mediated programmed cell death. Nature 404 (6780), 892–897. 10.1038/35009130 10786798

[B24] Salkeni M. A. Shin S. Takebe N. Stevens S. Chen A. (2024). Advanced granulosa cell tumors of the ovary: a review with a focus on current and novel therapeutic approaches. J. Immunother. Precis. Oncol. 7 (4), 263–271. 10.36401/JIPO-23-40 39524463 PMC11541922

[B25] Schmid N. Dietrich K. G. Forne I. Burges A. Szymanska M. Meidan R. (2021). Sirtuin 1 and sirtuin 3 in granulosa cell tumors. Int. J. Mol. Sci. 22 (4), 2047. 10.3390/ijms22042047 33669567 PMC7923107

[B26] Shi Y. He R. Yang Y. He Y. Zhan L. Wei B. (2020). Potential relationship between Sirt3 and autophagy in ovarian cancer. Oncol. Lett. 20 (5), 162. 10.3892/ol.2020.12023 32934730 PMC7471650

[B27] Signorile A. De Rasmo D. Cormio A. Musicco C. Rossi R. Fortarezza F. (2019). Human ovarian cancer tissue exhibits increase of mitochondrial biogenesis and cristae remodeling. Cancers (Basel) 11 (9), 1350. 10.3390/cancers11091350 31547300 PMC6770021

[B28] Tan J. Sun M. Yin J. Zhou Q. Zhao R. Chen Q. (2022). Hsa_circ_0005050 interacts with ILF3 and affects cell apoptosis and proliferation by disrupting the balance between p53 and p65. Chem. Biol. Interact. 368, 110208. 10.1016/j.cbi.2022.110208 36208777

[B29] Wang C. Long Q. Fu Q. Xu Q. Fu D. Li Y. (2022). Targeting epiregulin in the treatment-damaged tumor microenvironment restrains therapeutic resistance. Oncogene 41 (45), 4941–4959. 10.1038/s41388-022-02476-7 36202915 PMC9630100

[B30] Wu Y. Gao W. N. Xue Y. N. Zhang L. C. Zhang J. J. Lu S. Y. (2018). SIRT3 aggravates metformin-induced energy stress and apoptosis in ovarian cancer cells. Exp. Cell Res. 367 (2), 137–149. 10.1016/j.yexcr.2018.03.030 29580688

[B31] Xiong Y. Wang L. Wang S. Wang M. Zhao J. Zhang Z. (2018). SIRT3 deacetylates and promotes degradation of P53 in PTEN-Defective non-small cell lung cancer. J. Cancer Res. Clin. Oncol. 144 (2), 189–198. 10.1007/s00432-017-2537-9 29103158 PMC11813316

[B32] Yin L. Yu X. (2018). Arsenic-induced apoptosis in the p53-proficient and p53-deficient cells through differential modulation of NFkB pathway. Food Chem. Toxicol. 118, 849–860. 10.1016/j.fct.2018.06.053 29944914 PMC6689409

[B33] Ying X. Zheng X. Zhang X. Yin Y. Wang X. (2024). Kynurenine in IDO1(high) cancer cell-derived extracellular vesicles promotes angiogenesis by inducing endothelial mitophagy in ovarian cancer. J. Transl. Med. 22 (1), 267. 10.1186/s12967-024-05054-5 38468343 PMC10929174

[B34] Zeng C. Li H. Liang W. Chen J. Zhang Y. Zhang H. (2024a). Loss of STARD13 contributes to aggressive phenotype transformation and poor prognosis in papillary thyroid carcinoma. Endocrine 83 (1), 127–141. 10.1007/s12020-023-03468-7 37541962

[B35] Zeng C. Zhang Y. Lin C. Liang W. Chen J. Chen Y. (2024b). TFCP2L1, a potential differentiation regulator, predicts favorable prognosis and dampens thyroid cancer progression. J. Endocrinol. Invest 47, 2953–2968. 10.1007/s40618-024-02392-5 38753296

[B36] Zhang J. Ye J. Zhu S. Han B. Liu B. (2024). Context-dependent role of SIRT3 in cancer. Trends Pharmacol. Sci. 45 (2), 173–190. 10.1016/j.tips.2023.12.005 38242748

[B37] Zhang Q. Ren J. Wang F. Pan M. Cui L. Li M. (2022). Mitochondrial and glucose metabolic dysfunctions in granulosa cells induce impaired oocytes of polycystic ovary syndrome through sirtuin 3. Free Radic. Biol. Med. 187, 1–16. 10.1016/j.freeradbiomed.2022.05.010 35594990

[B38] Zhao D. Song Y. Zhang Y. Li B. (2019). Outcomes of fertility-sparing surgery in ovarian juvenile granulosa cell tumor. Int. J. Gynecol. Cancer 29 (4), 787–791. 10.1136/ijgc-2018-000083 30728165

[B39] Zhou L. Pinho R. Gu Y. Radak Z. (2022). The role of SIRT3 in exercise and aging. Cells 11 (16), 2596. 10.3390/cells11162596 36010672 PMC9406297

[B40] Zhou Y. Li C. Peng J. Xie L. Meng L. Li Q. (2018). DNA-encoded dynamic chemical library and its applications in ligand discovery. J. Am. Chem. Soc. 140 (46), 15859–15867. 10.1021/jacs.8b09277 30412395

