# Supplementary material for: Inhibition of SIRT3 by a specific inhibitor induces cellular senescence and growth arrest of ovarian granulosa cell tumor via p53 and NF-κB axis
Source: Front Pharmacol. 2025 Jul 24;16:1608156. doi: 10.3389/fphar.2025.1608156 (PMC12328414; doi:10.3389/fphar.2025.1608156)
Supplement: Supplementary file 1 [file DataSheet1.pdf]

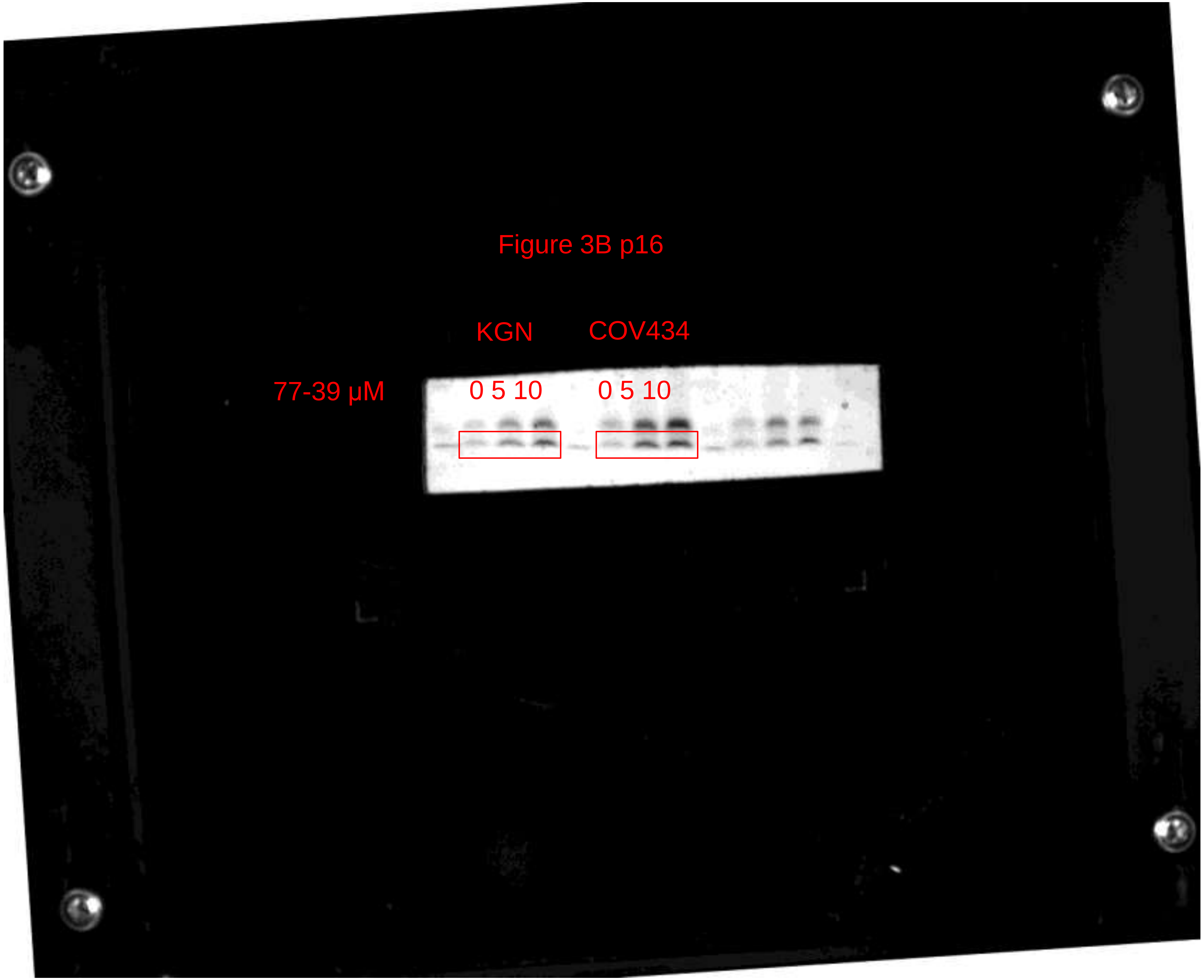
Figure 3B p16
KGN
COV434
77-39 μM
0 5 10
0 5 10

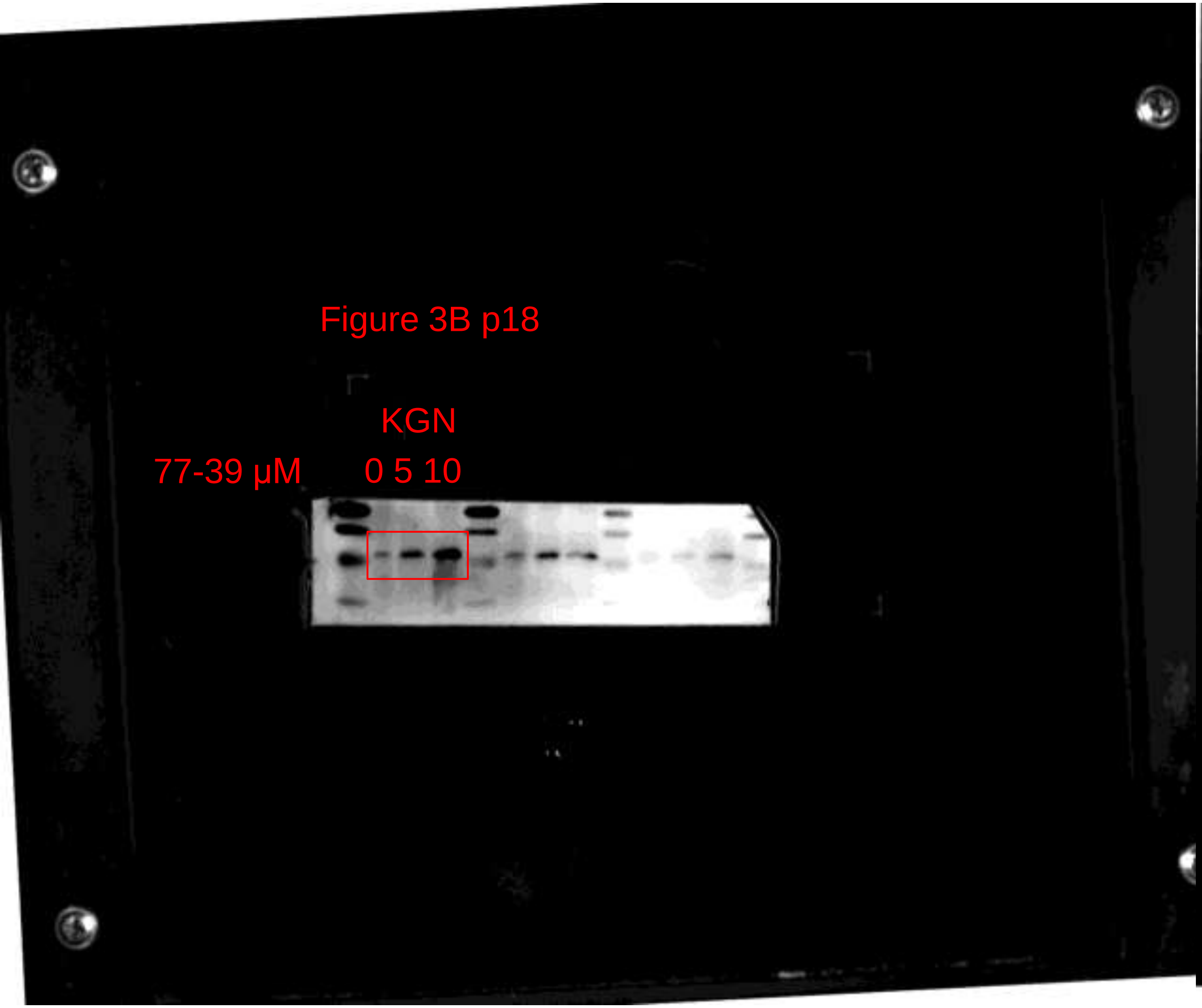
Figure 3B p18
KGN
77-39 μM
0 5 10

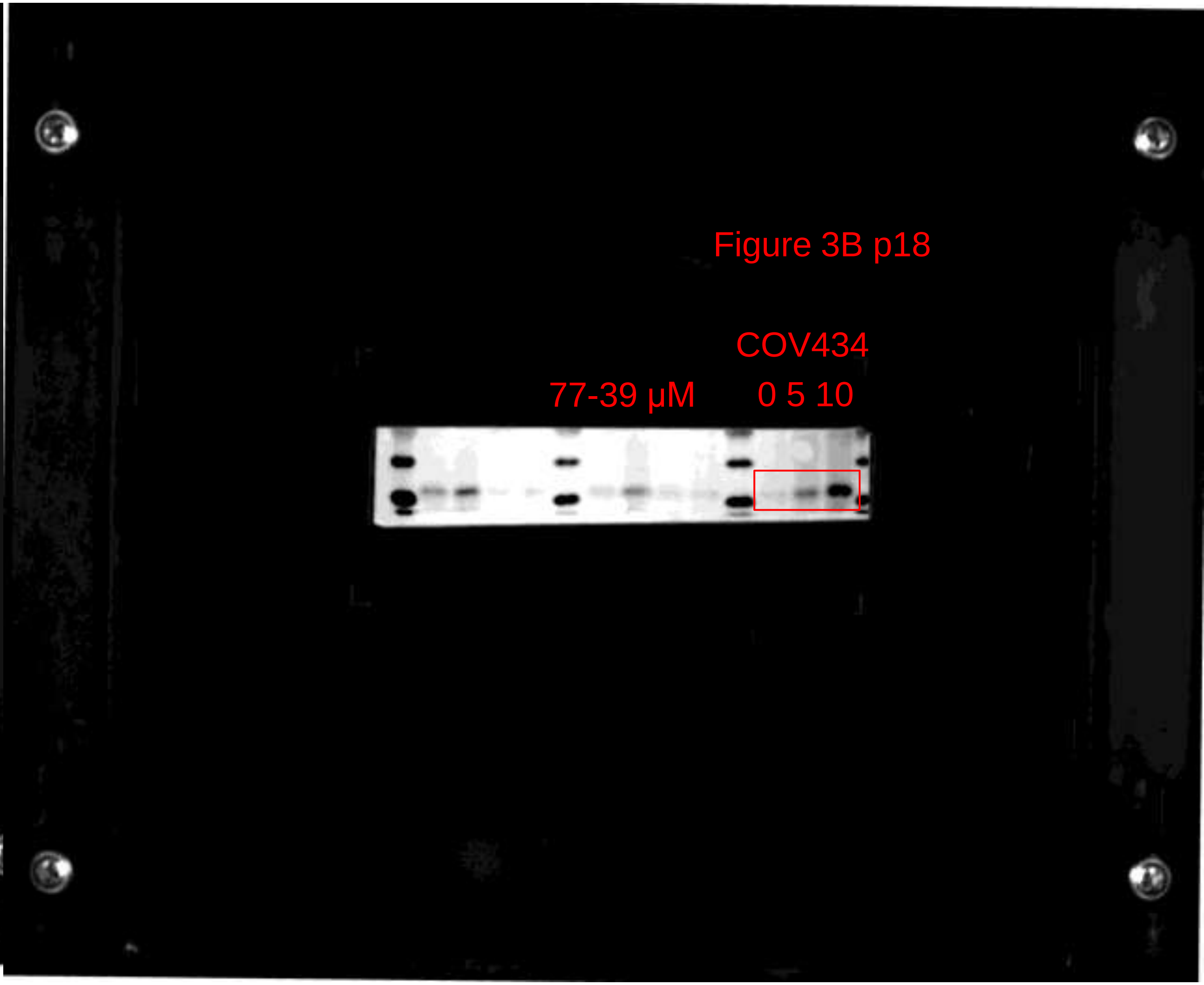
Figure 3B p18
COV434
77-39 μM
0 5 10

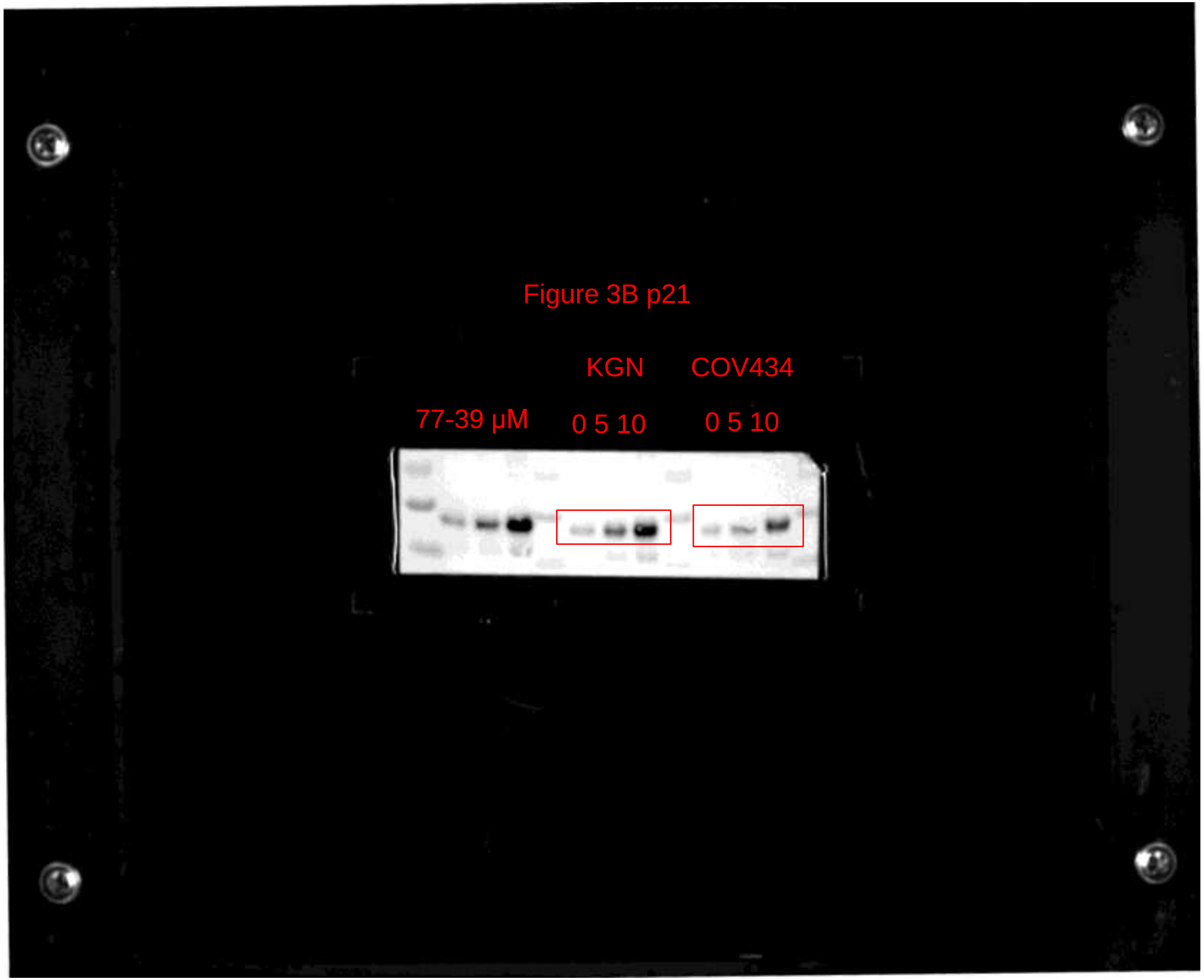
Figure 3B p21
KGN
COV434
77-39 μM
0 5 10
0 5 10

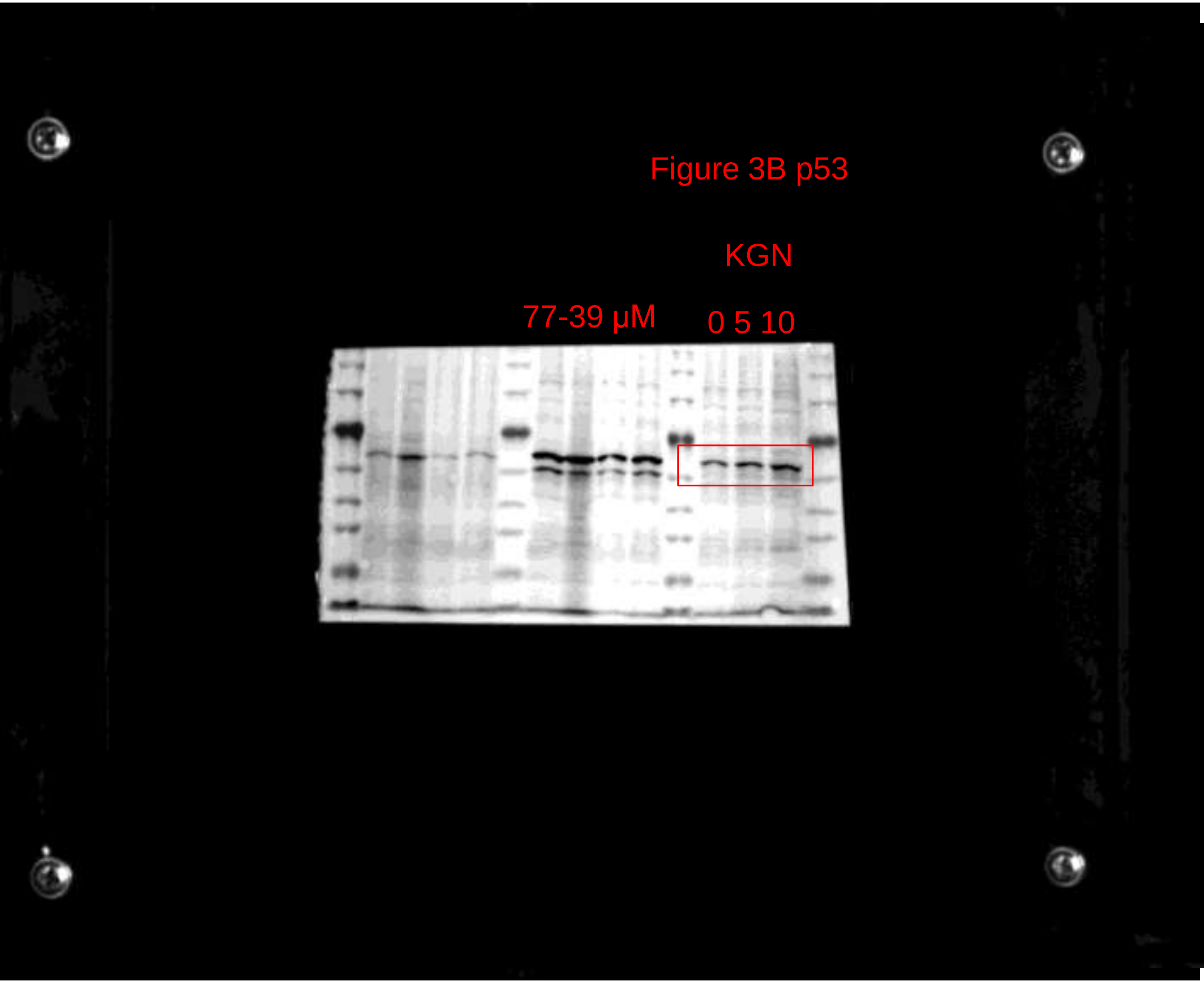
Figure 3B p53
KGN
77-39 μM
0 5 10

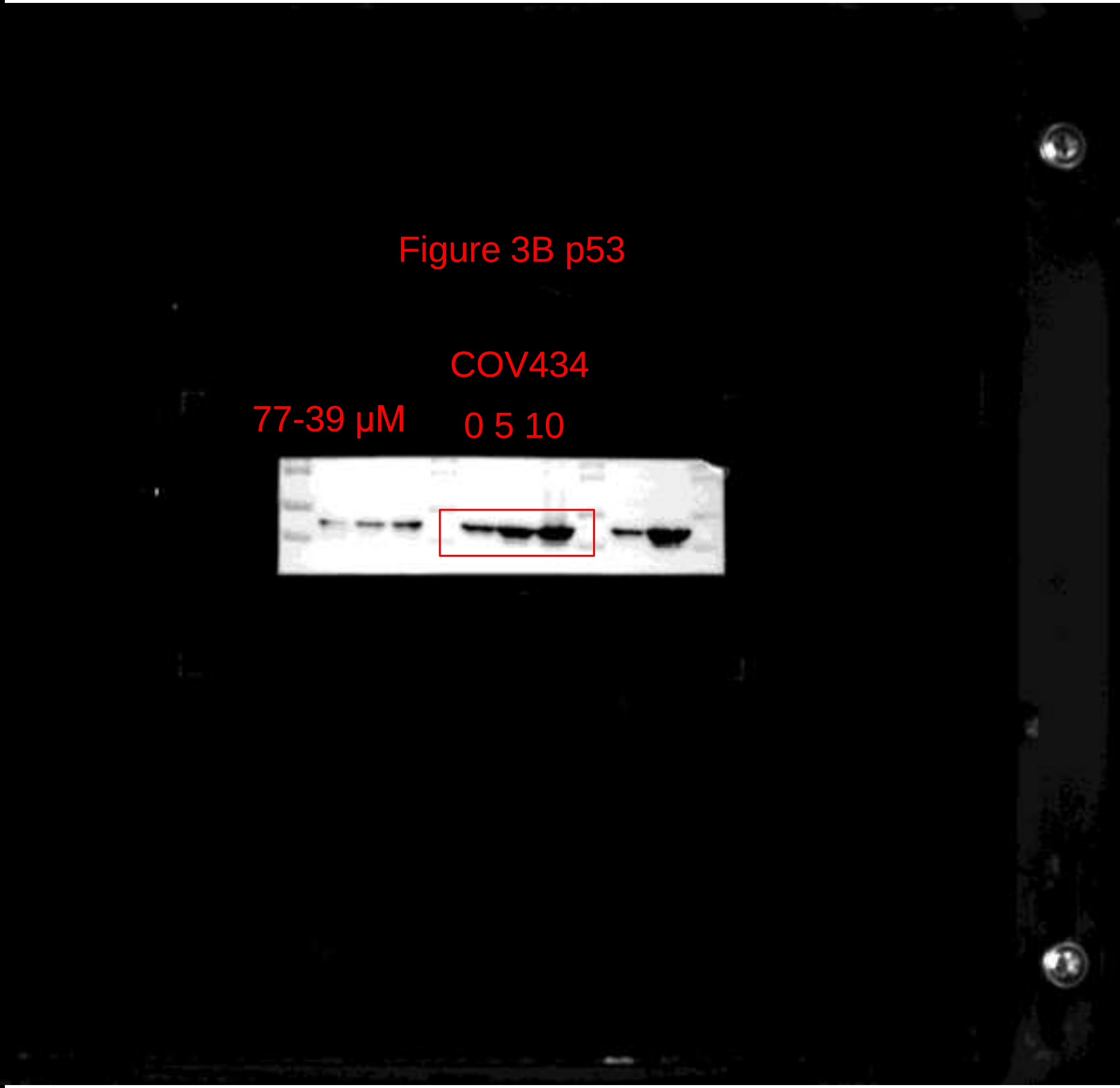
Figure 3B p53
COV434
77-39 μM
0 5 10

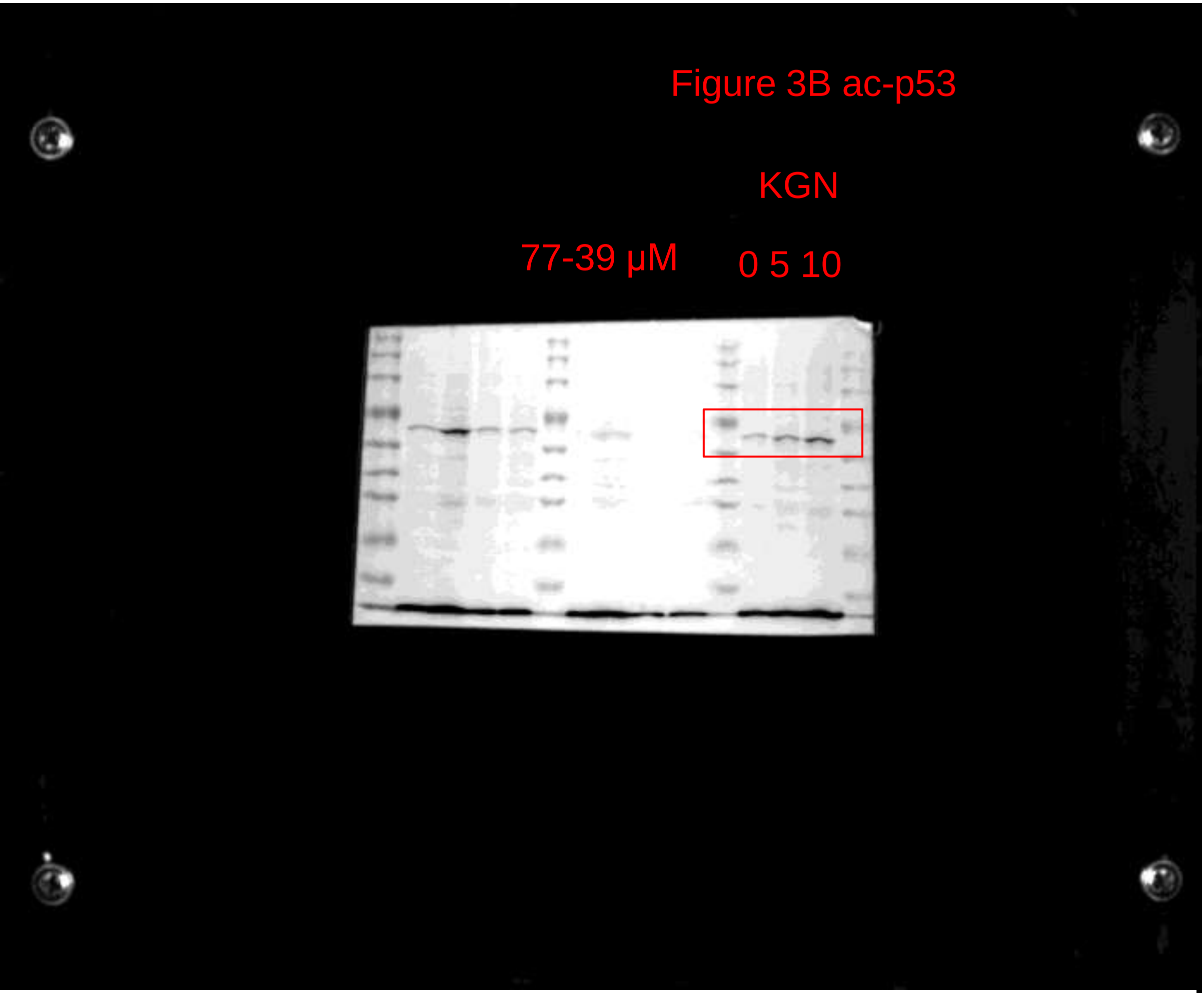
Figure 3B ac-p53
KGN
77-39 μM
0 5 10

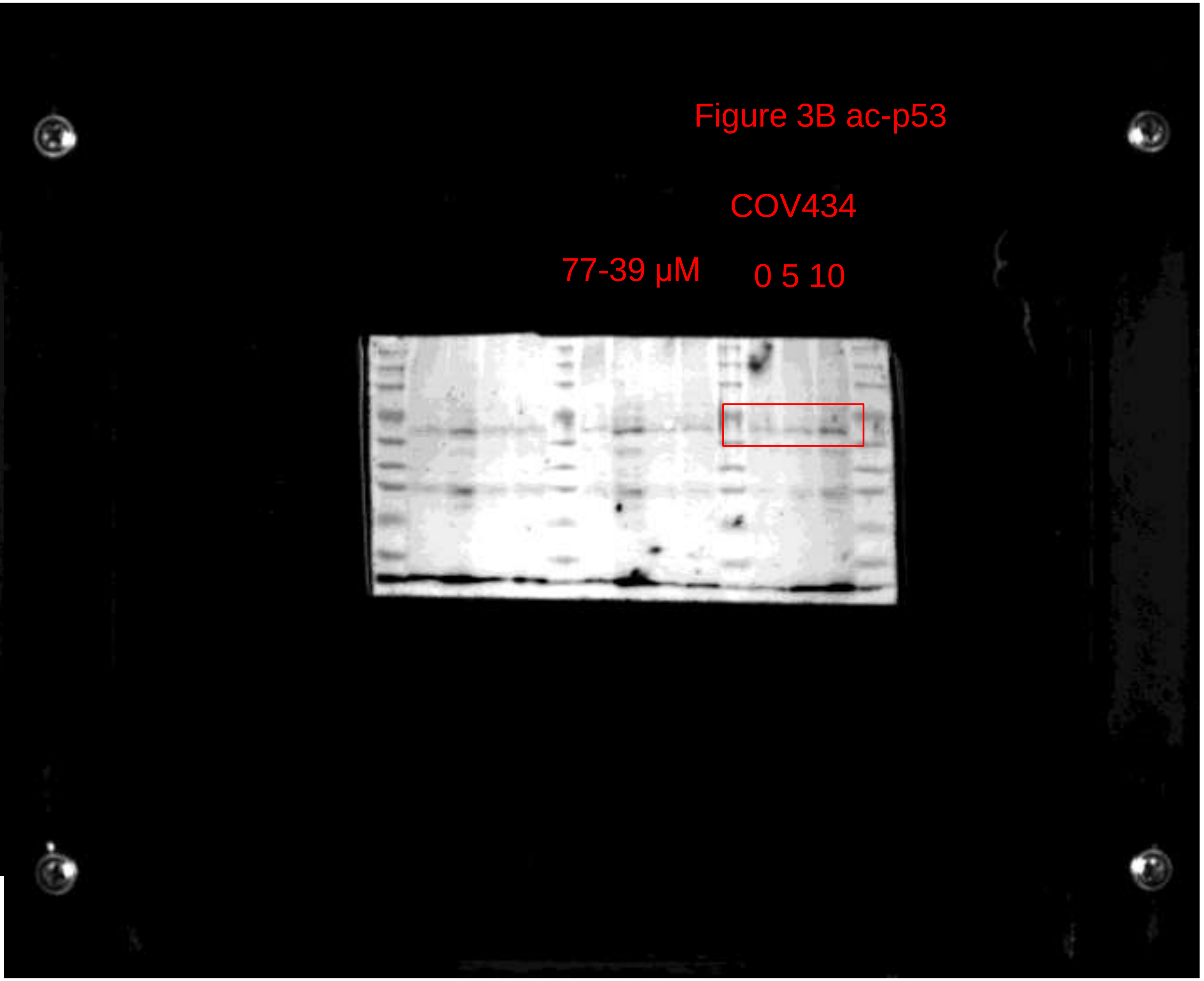
Figure 3B ac-p53
COV434
77-39 μM
0 5 10

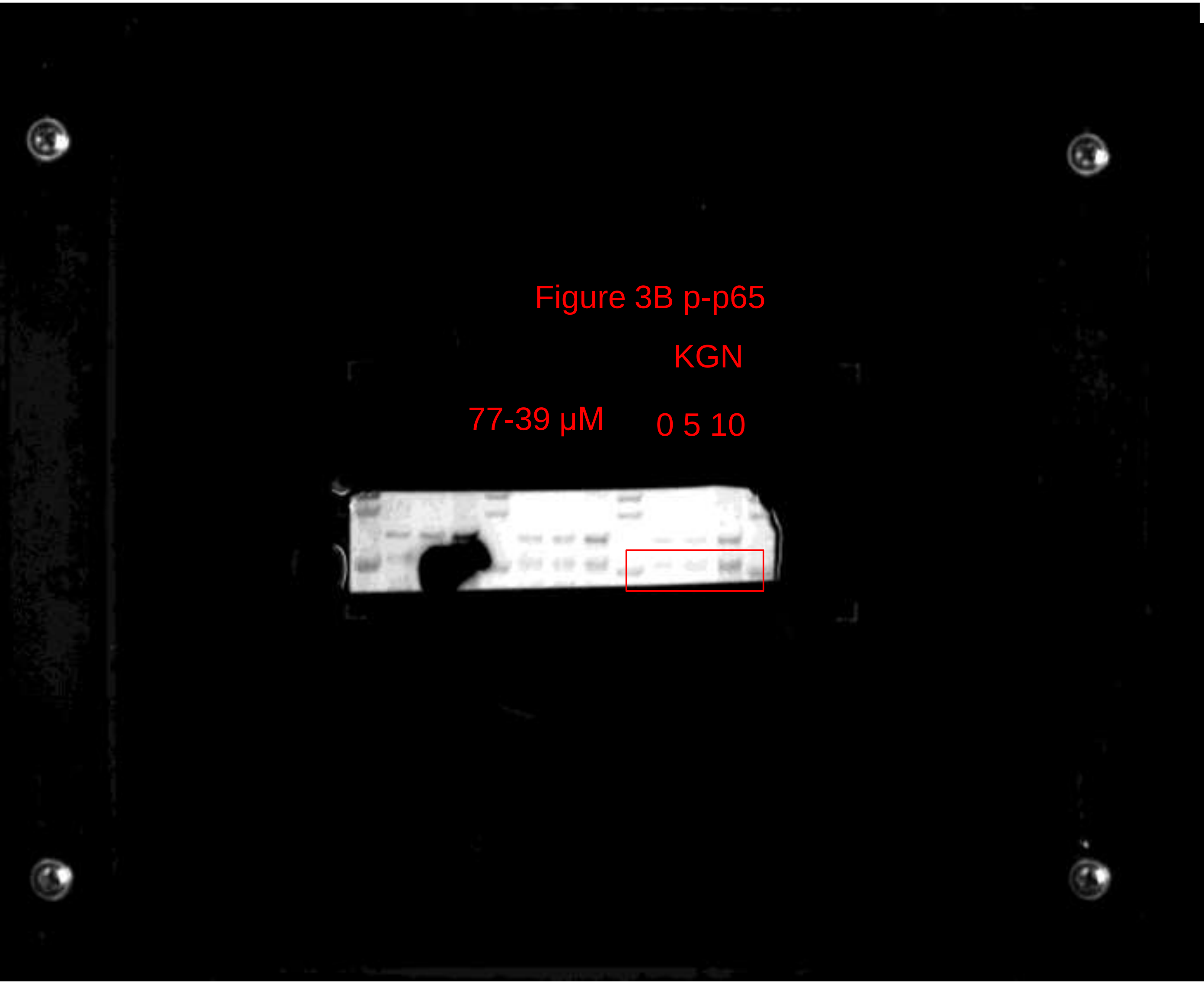
Figure 3B p-p65
KGN
77-39 μM
0 5 10

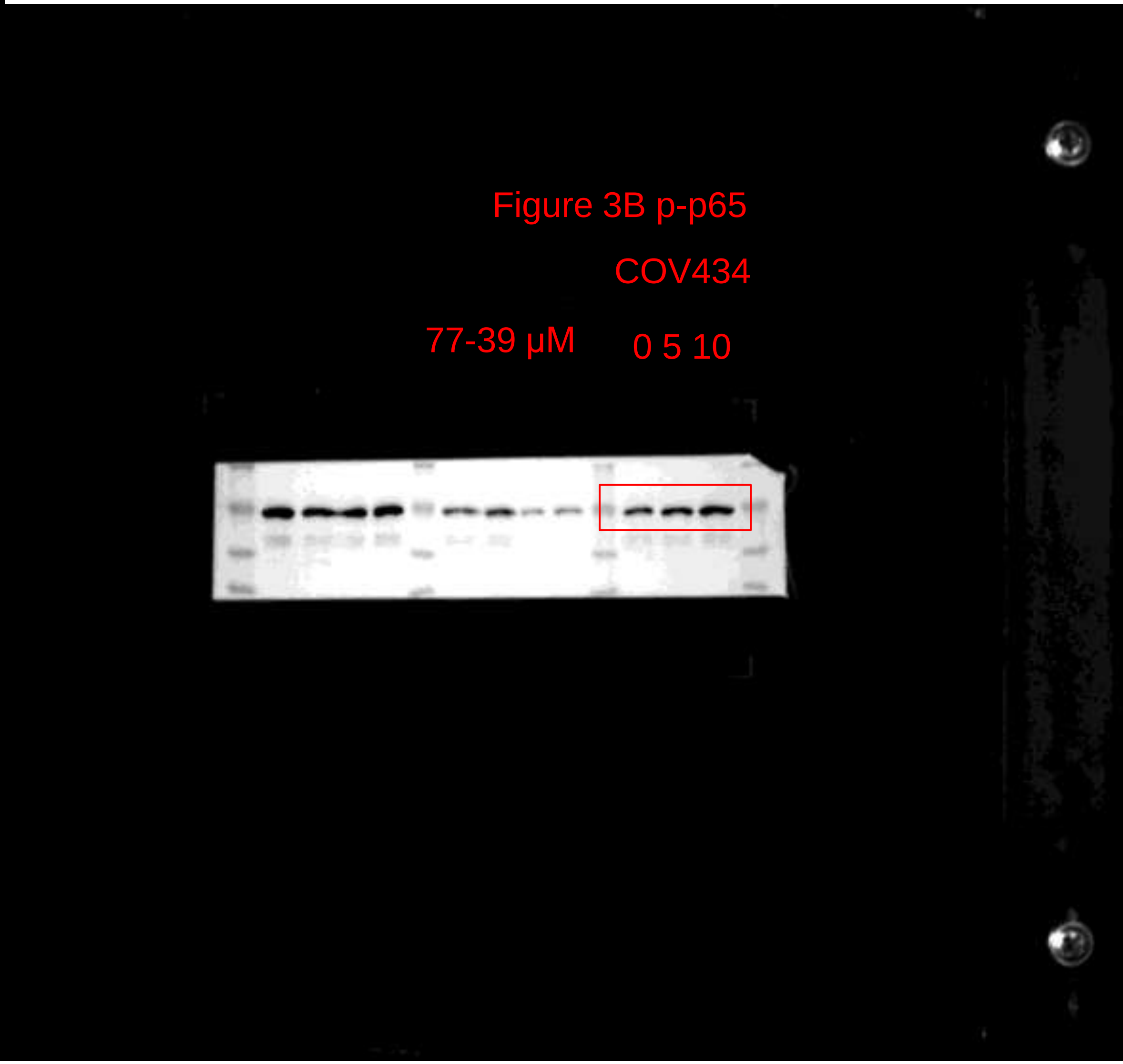
Figure 3B p-p65
COV434
77-39 μM
0 5 10

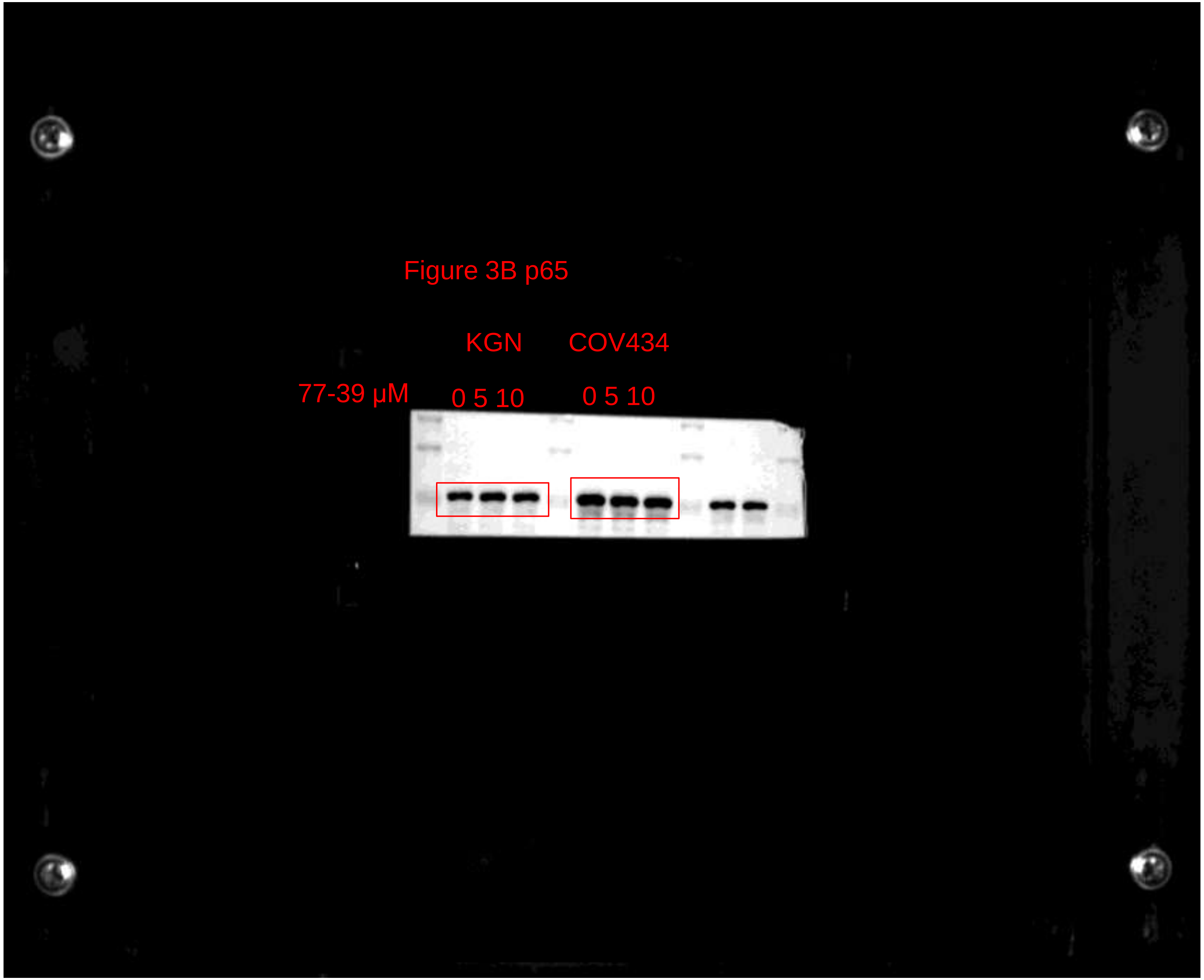
Figure 3B p65
KGN
COV434
77-39 μM
0 5 10
0 5 10

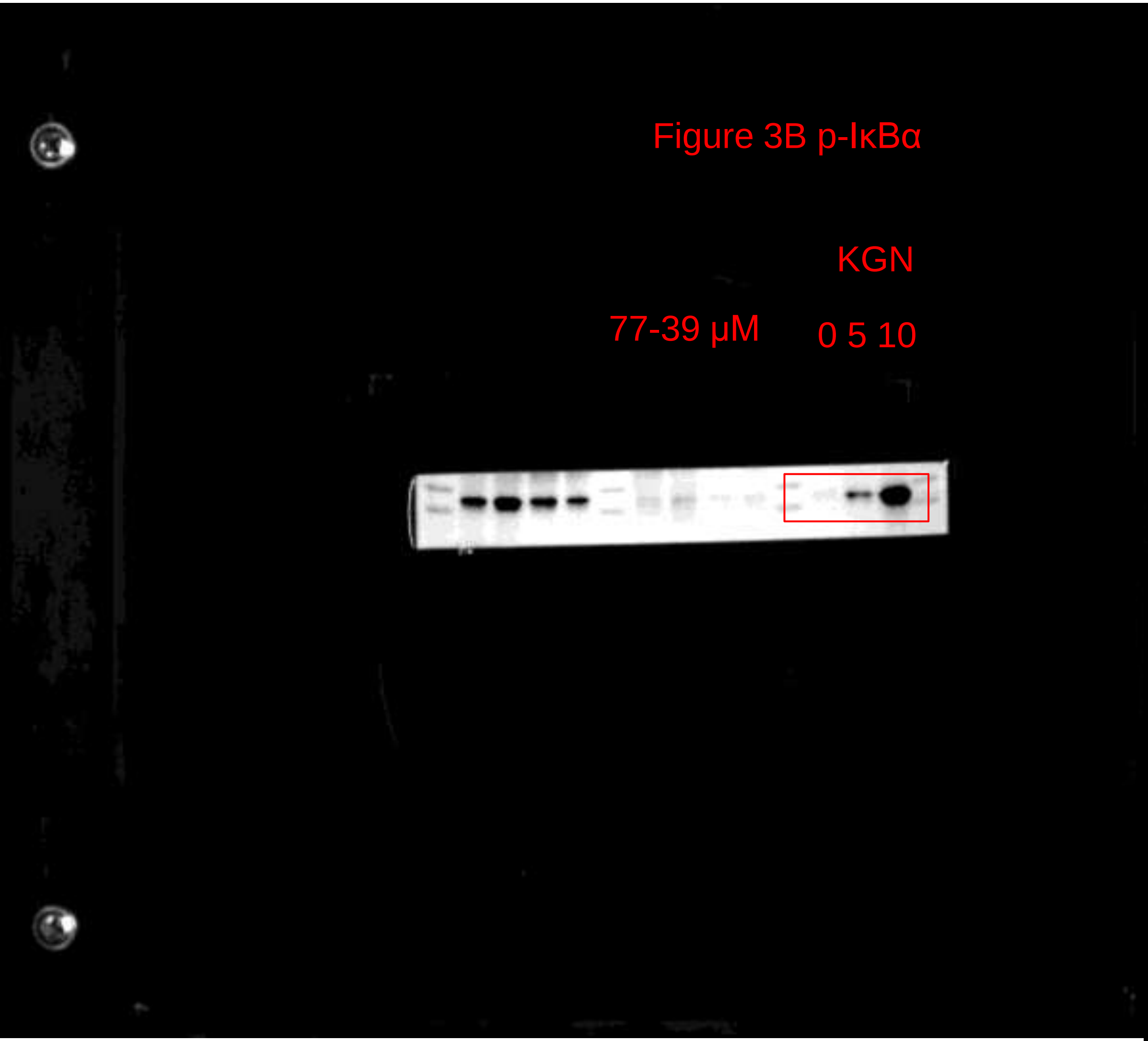
Figure 3B p-IκBα
KGN
77-39 μM
0 5 10

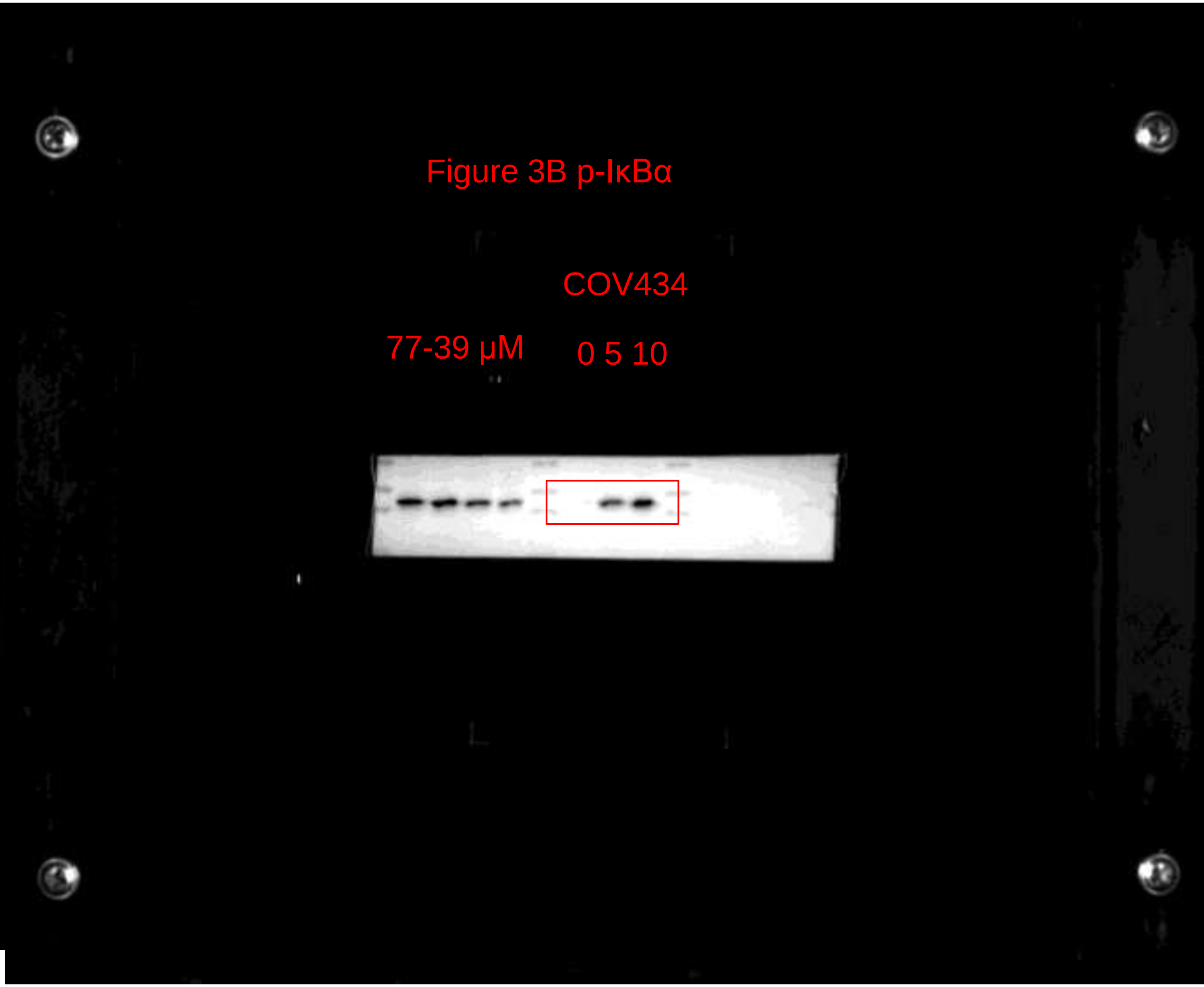
Figure 3B p-IκBα
COV434
77-39 μM
0 5 10

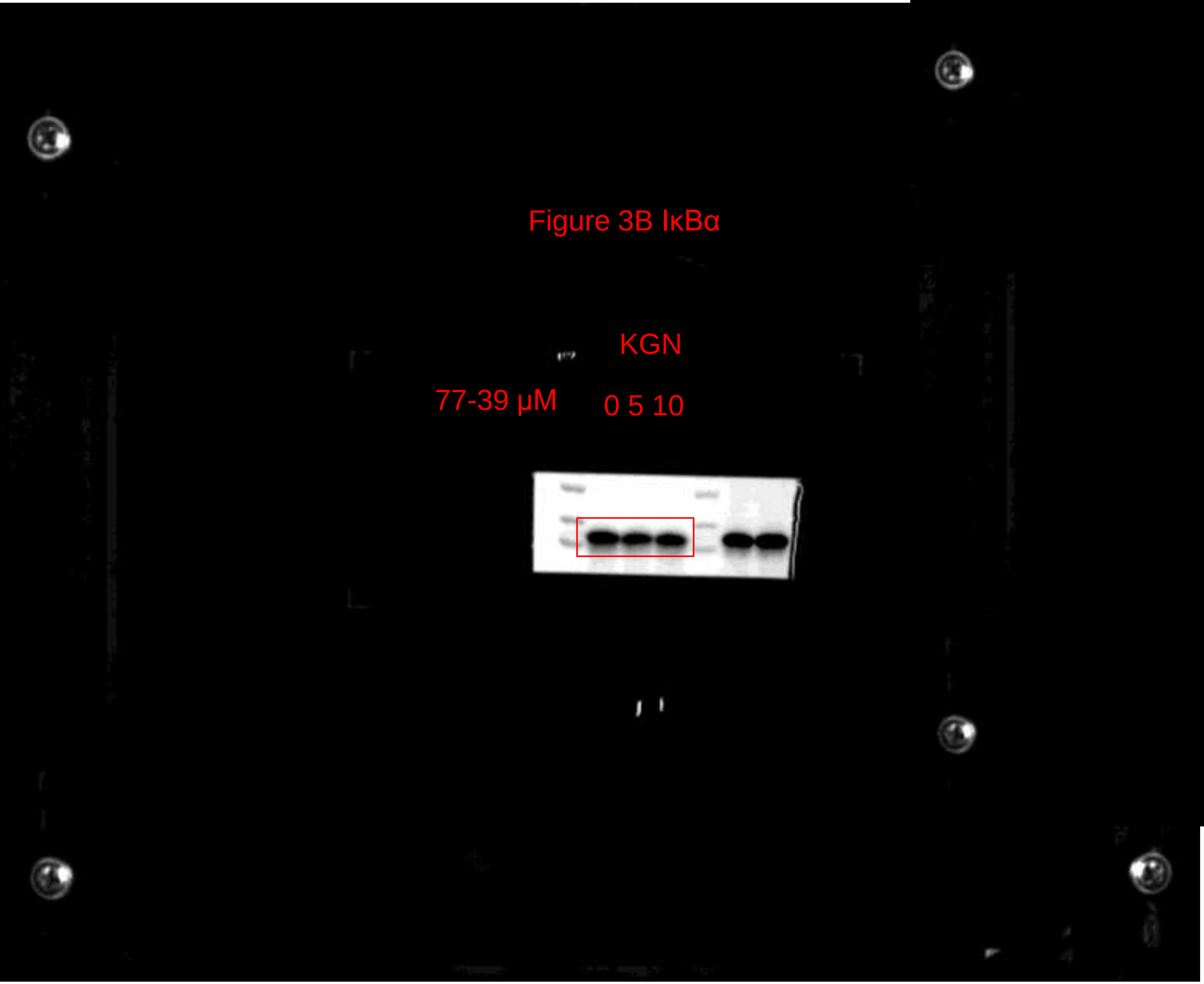
Figure 3B IκBα
KGN
77-39 μM
0 5 10

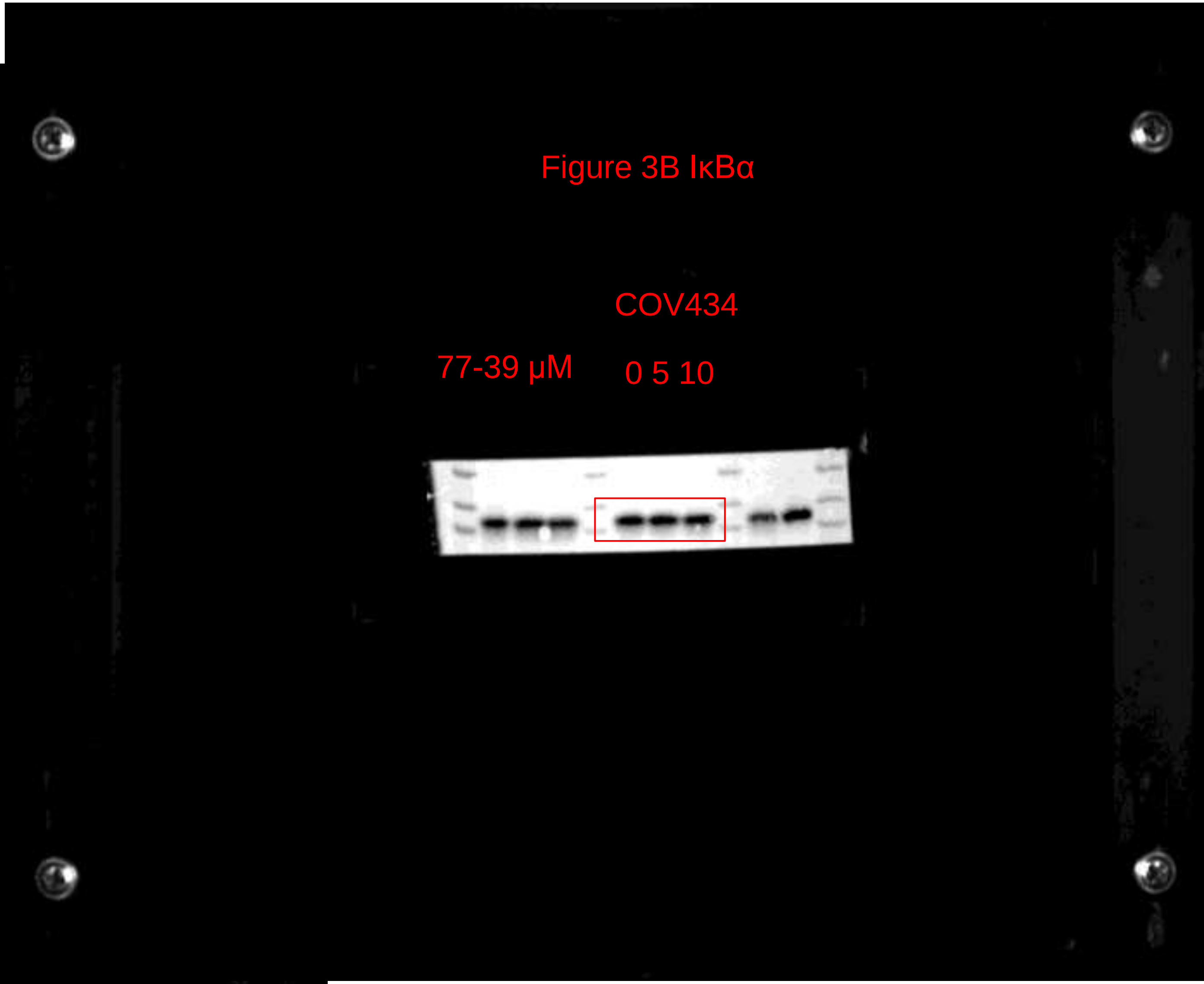
Figure 3B IκBα
COV434
77-39 μM
0 5 10

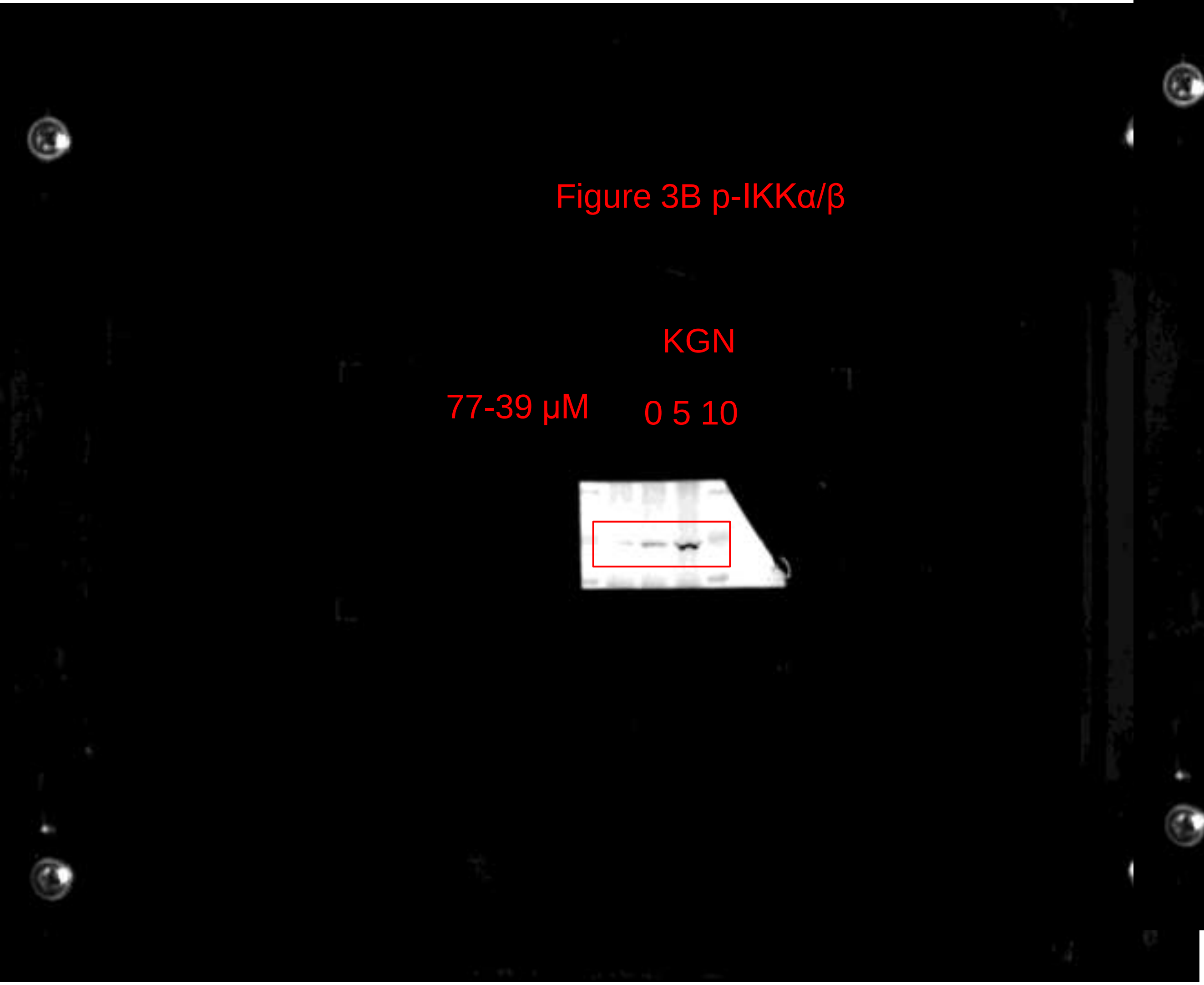

Figure 3B p-IKKα/β
KGN
77-39 μM
0 5 10

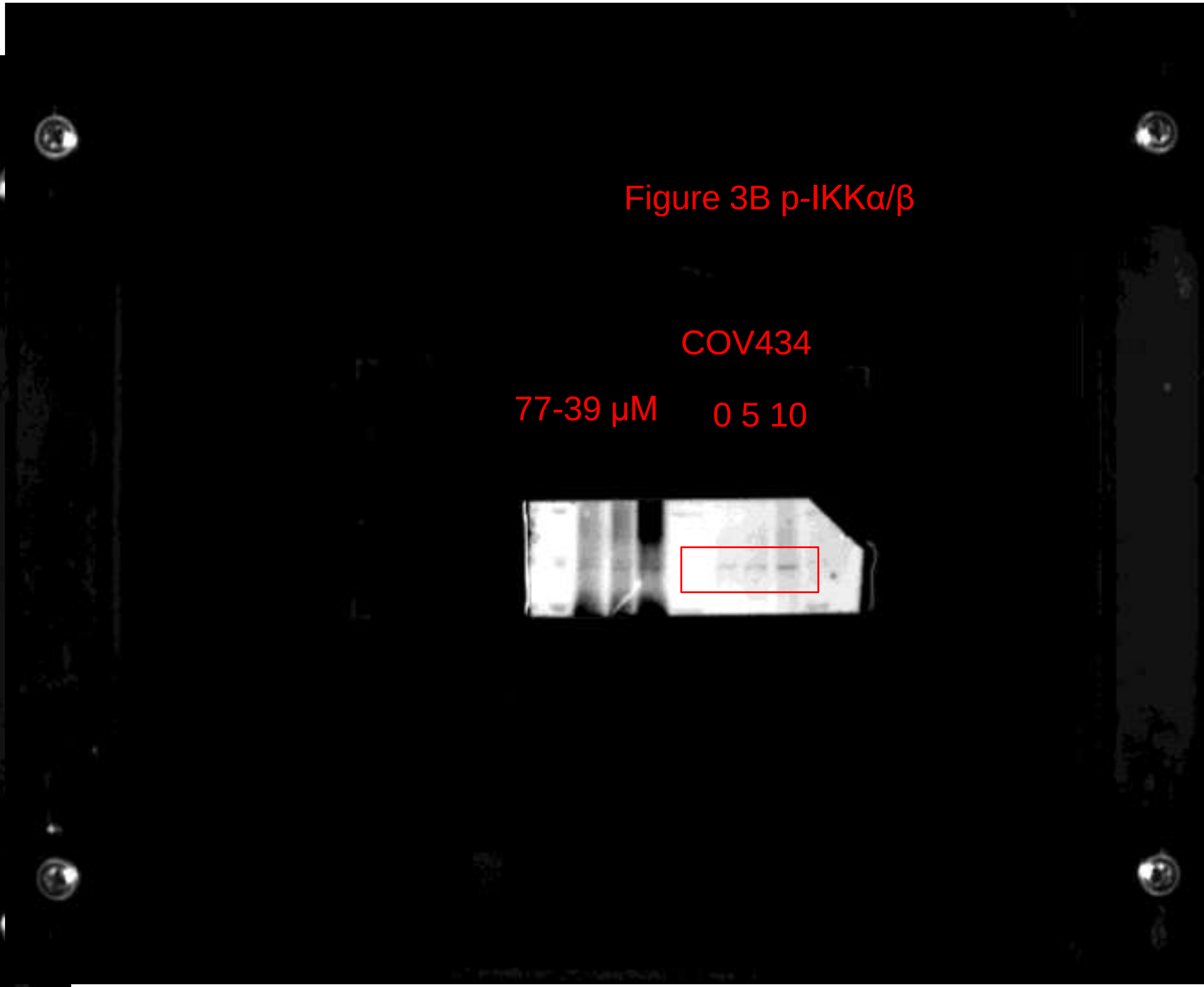

Figure 3B p-IKKα/β
COV434
77-39 μM
0 5 10

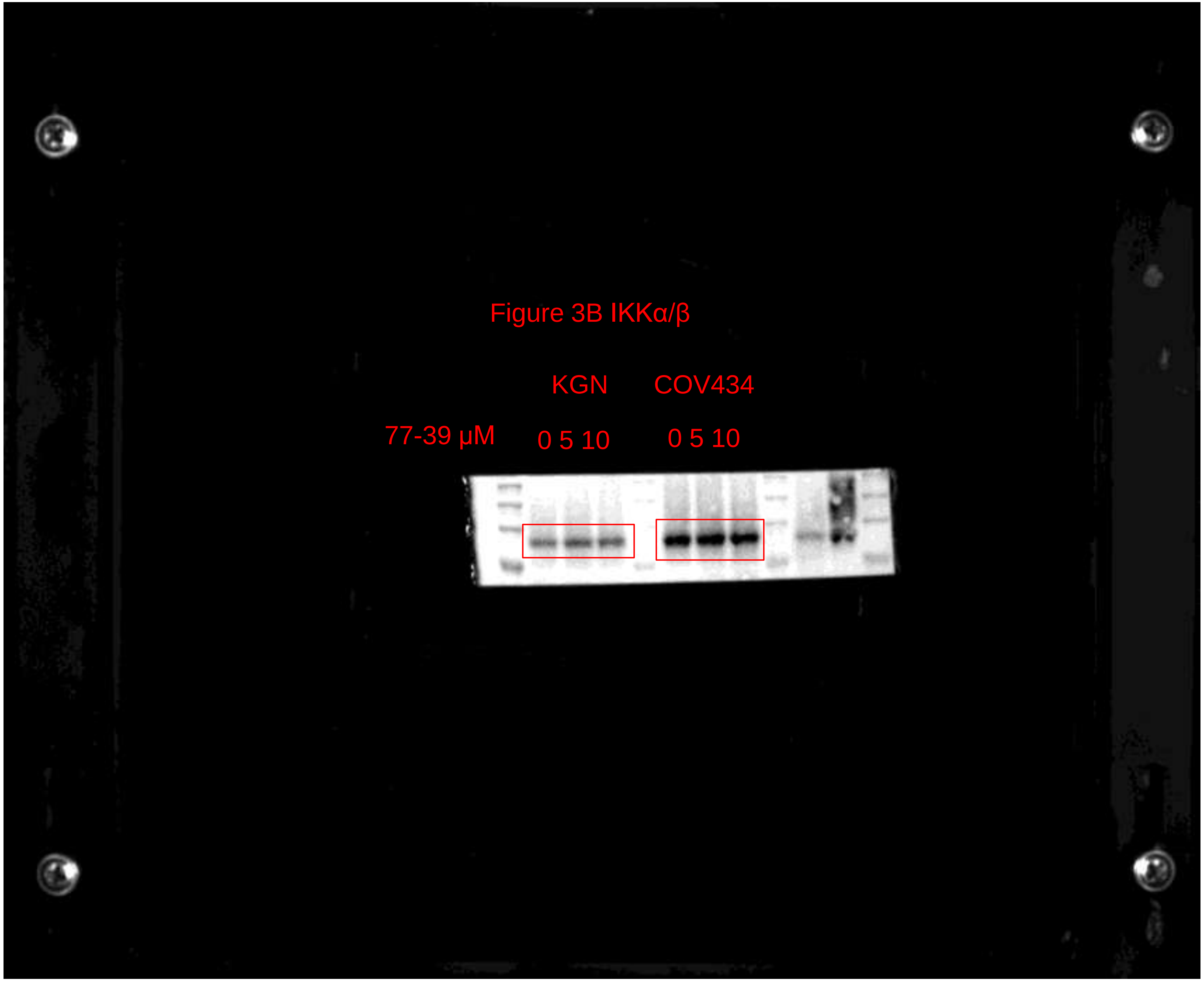
Figure 3B IKKα/β
KGN
COV434
77-39 μM
0 5 10
0 5 10

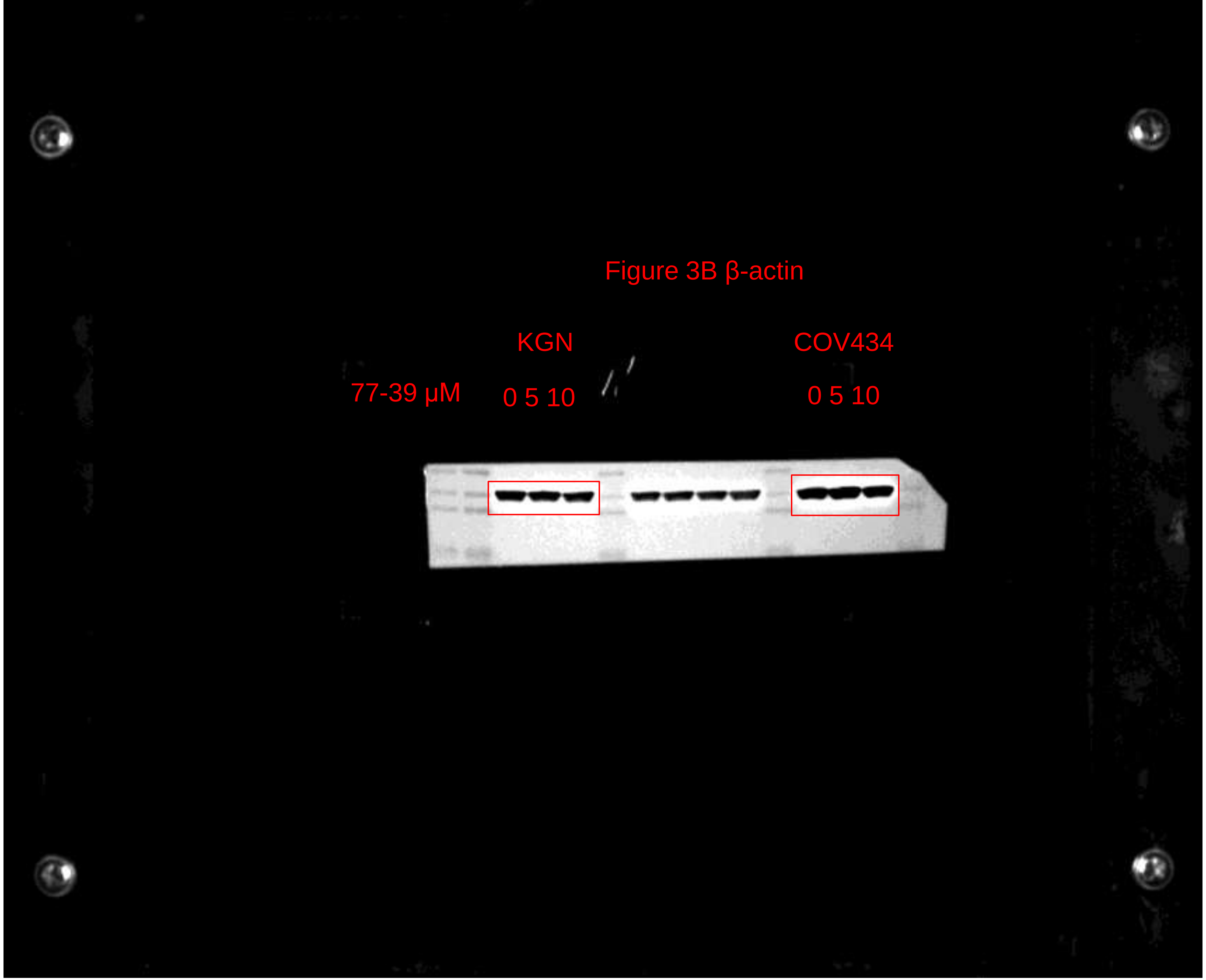
Figure 3B β-actin
KGN
COV434
77-39 μM
0 5 10
0 5 10

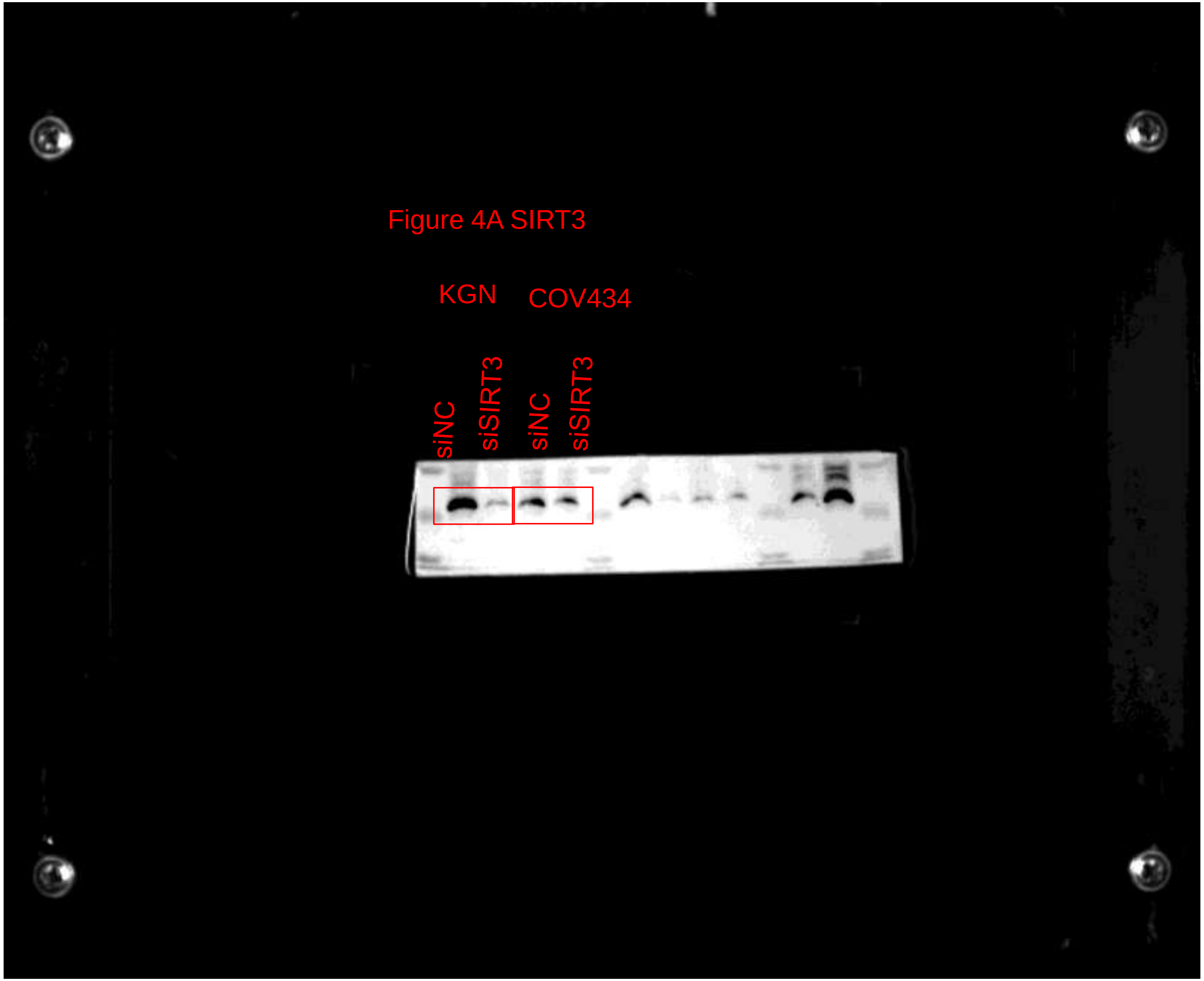
Figure 4A SIRT3
KGN
COV434
siNC
siSIRT3
siNC
siSIRT3

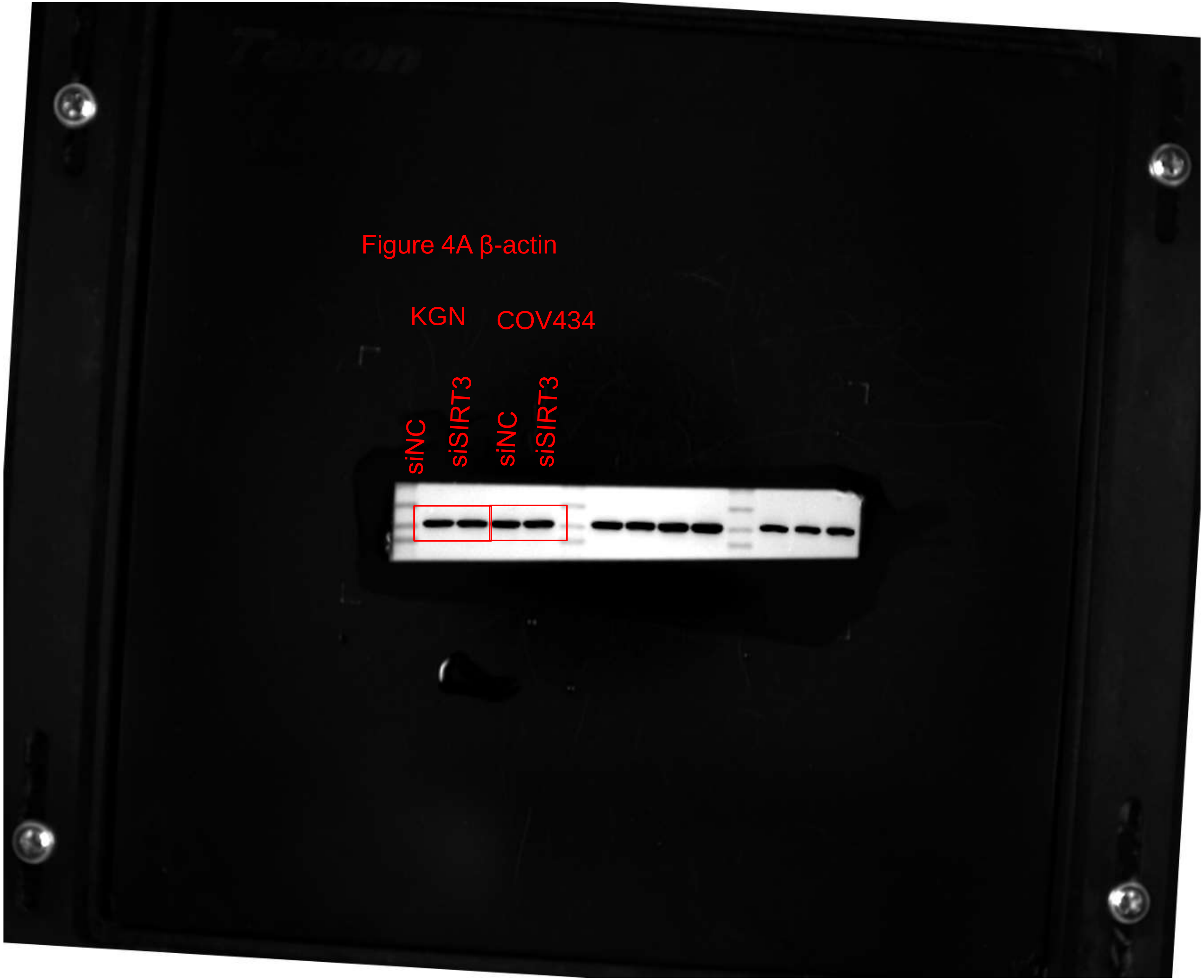

Figure 4A β-actin

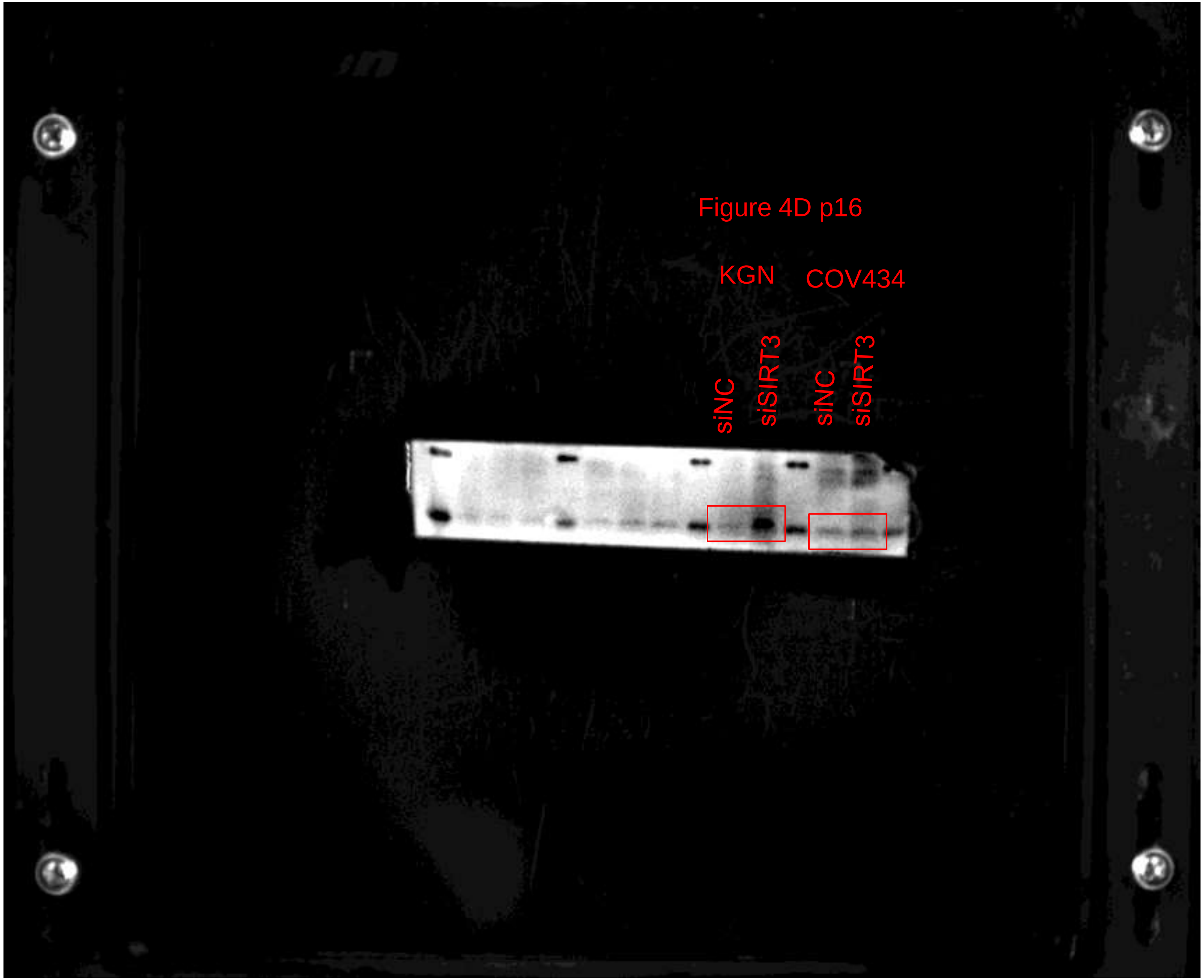
Figure 4D p16
KGN
COV434
siNC
siSIRT3
siNC
siSIRT3

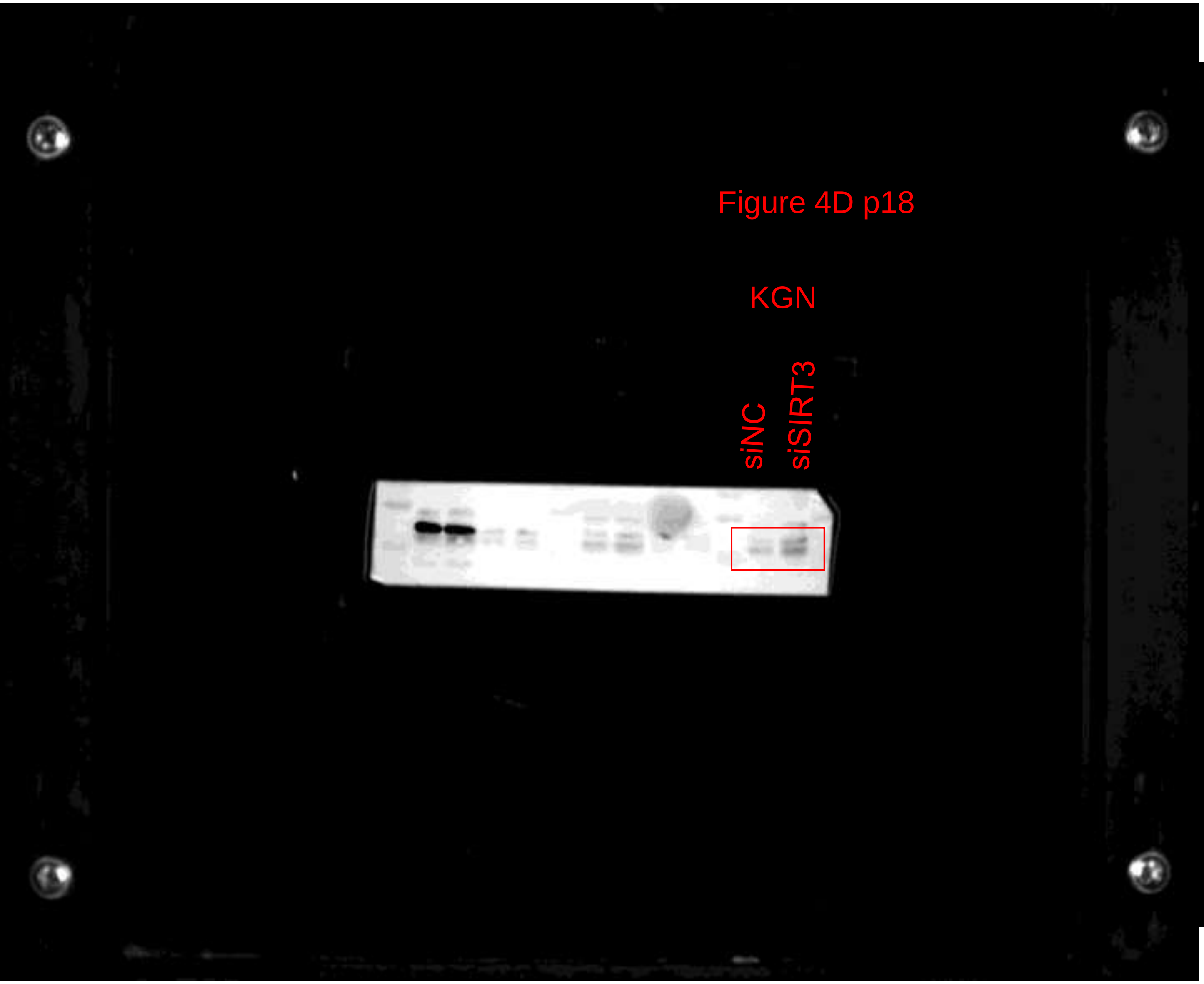
Figure 4D p18
KGN
siNC
siSIRT3

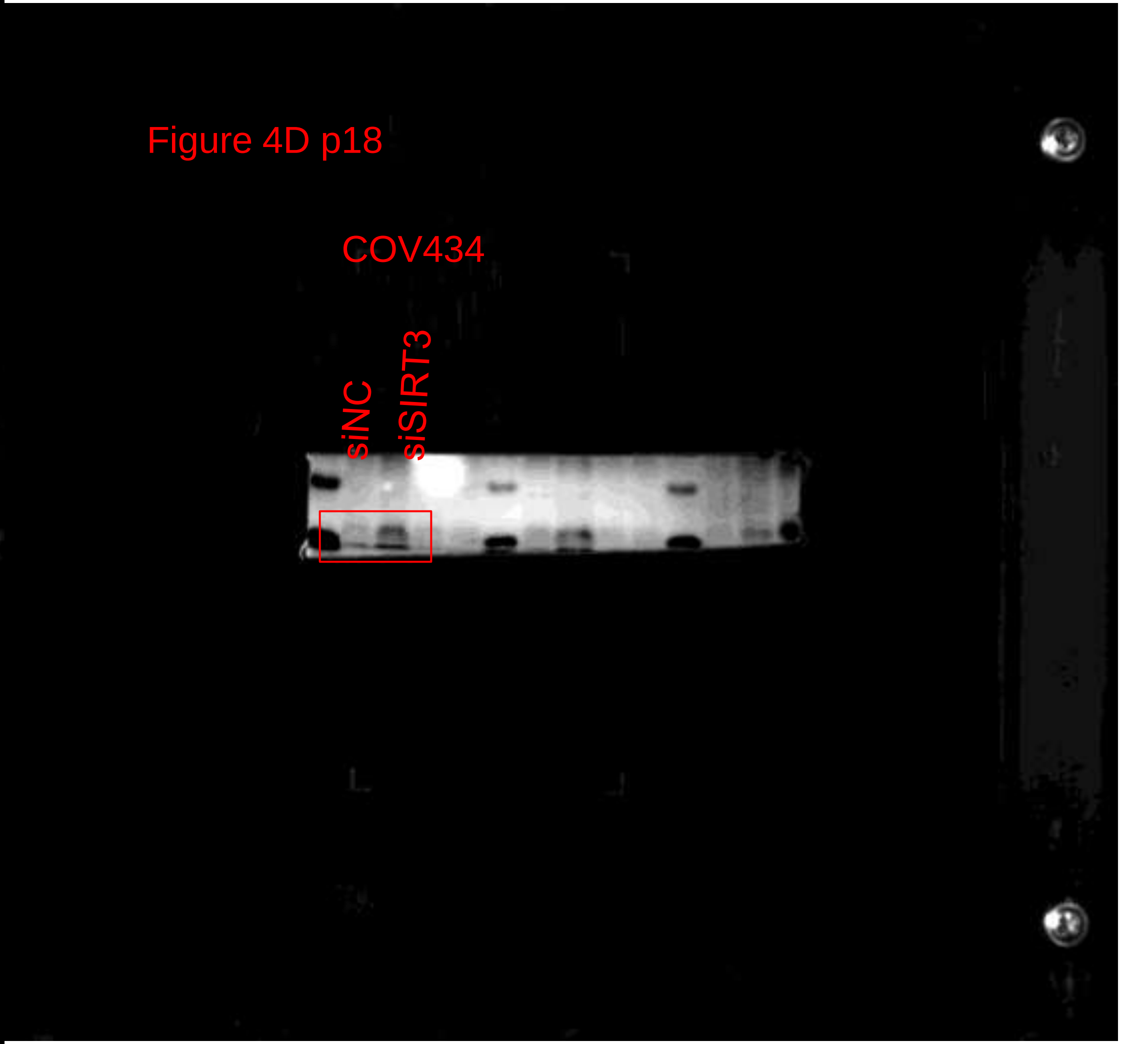
Figure 4D p18
COV434
siNC
siSIRT3

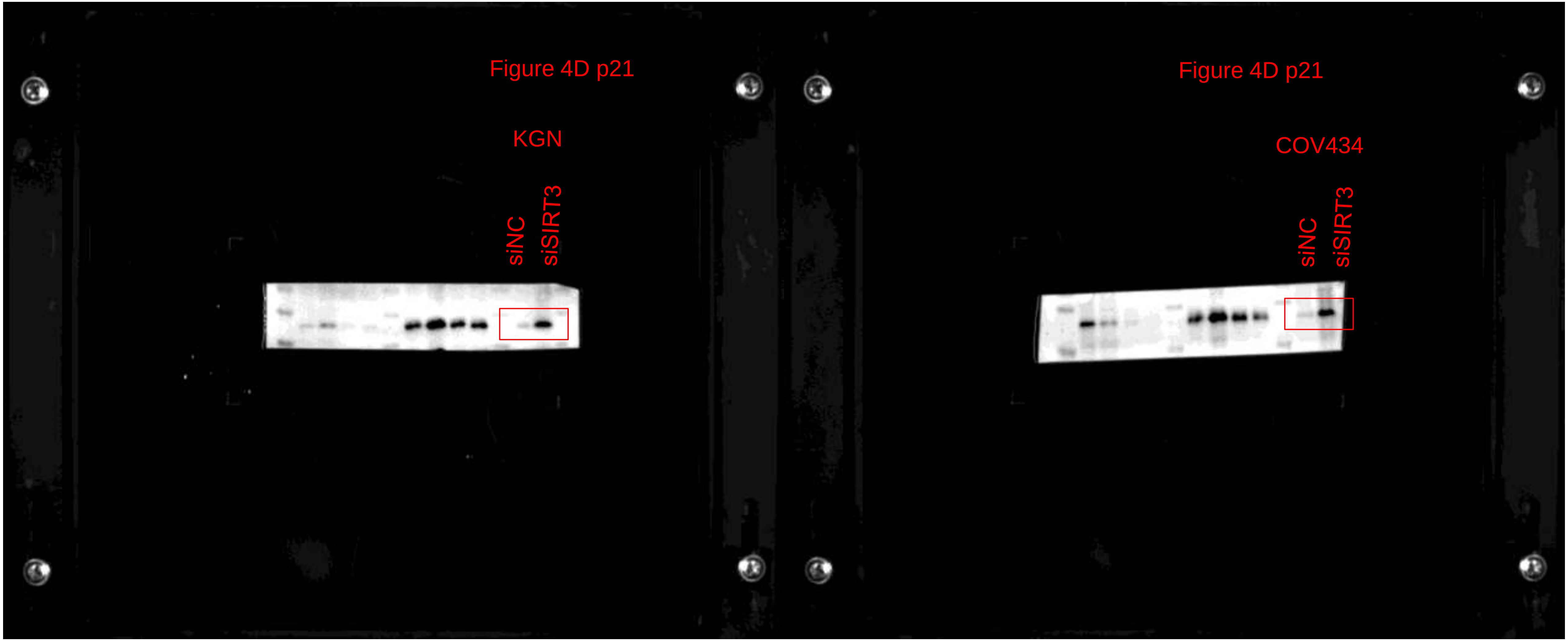
Figure 4D p21
KGN
siNC
siSIRT3
Figure 4D p21
COV434
siNC
siSIRT3

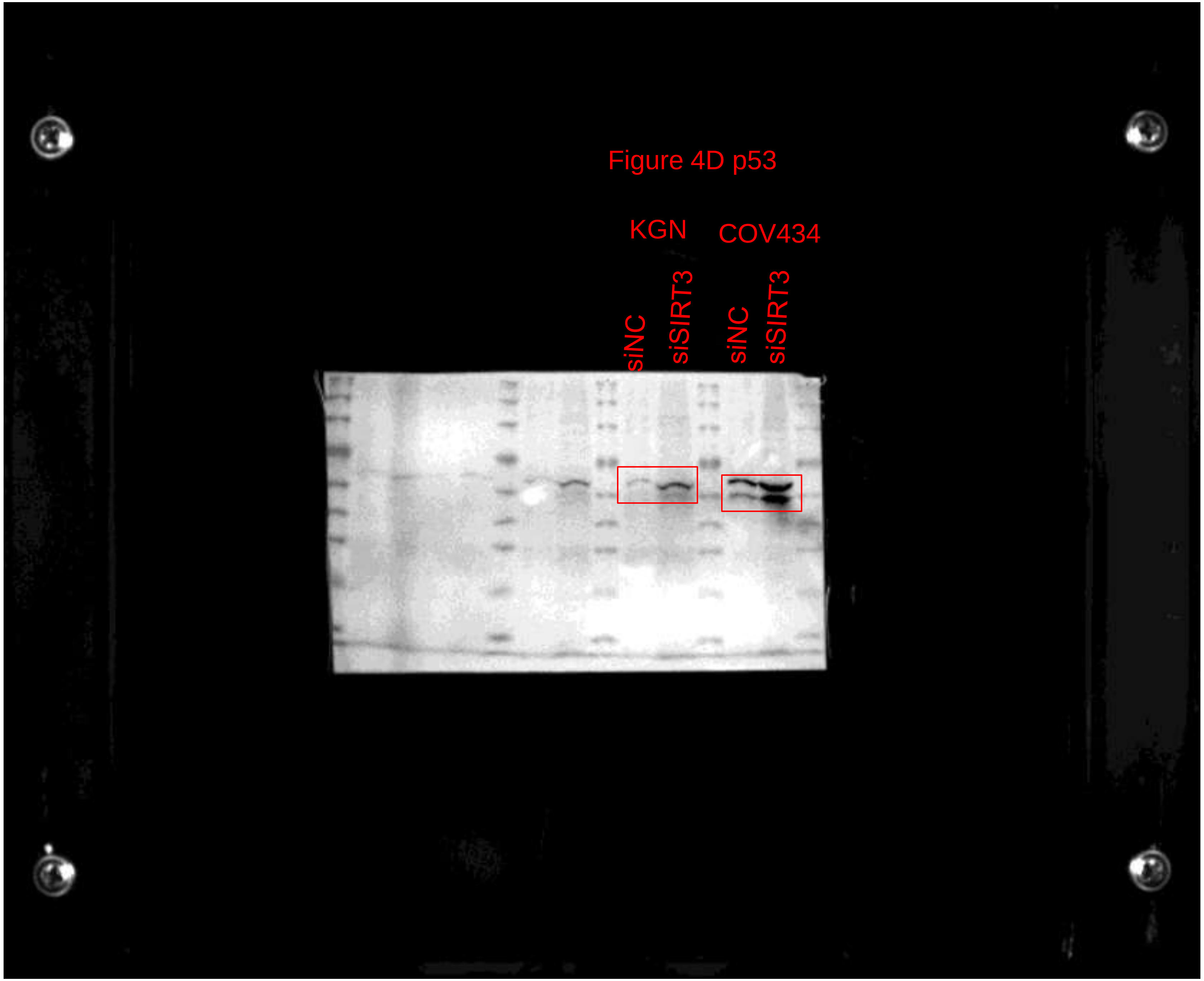
Figure 4D p53
KGN
COV434
siNC
siSIRT3
siNC
siSIRT3

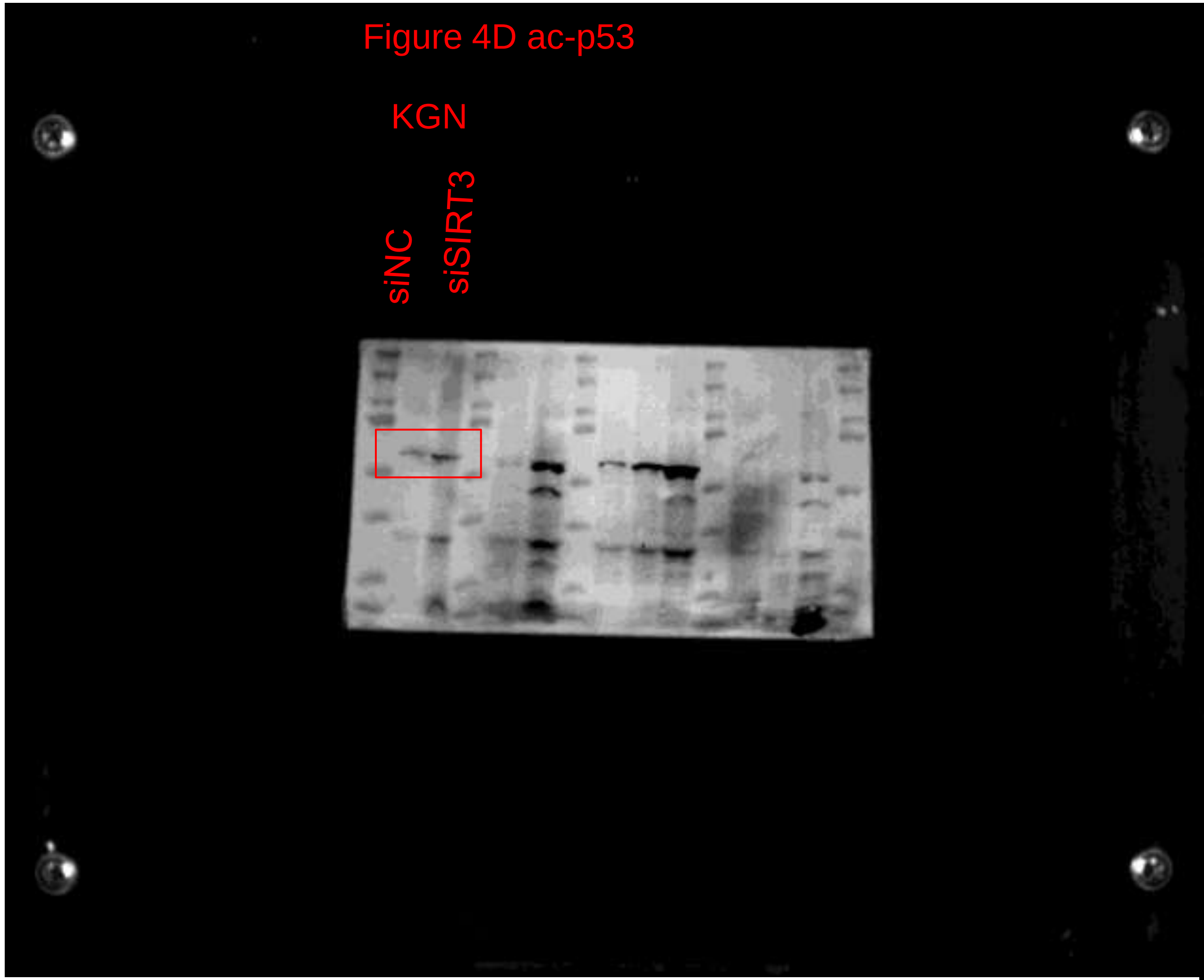

Figure 4D ac-p53
KGN
siNC
siSIRT3

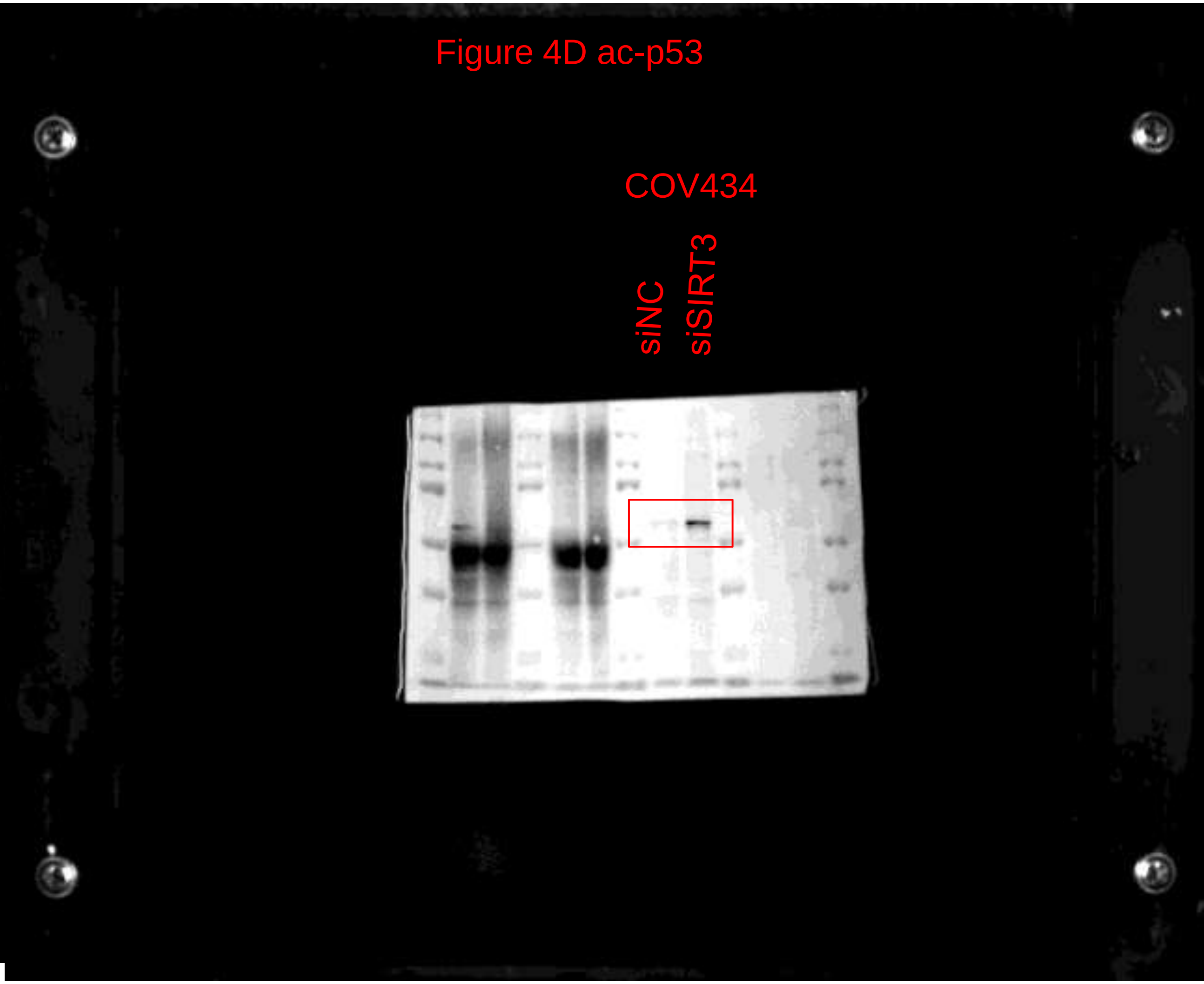

Figure 4D ac-p53
COV434
siNC
siSIRT3

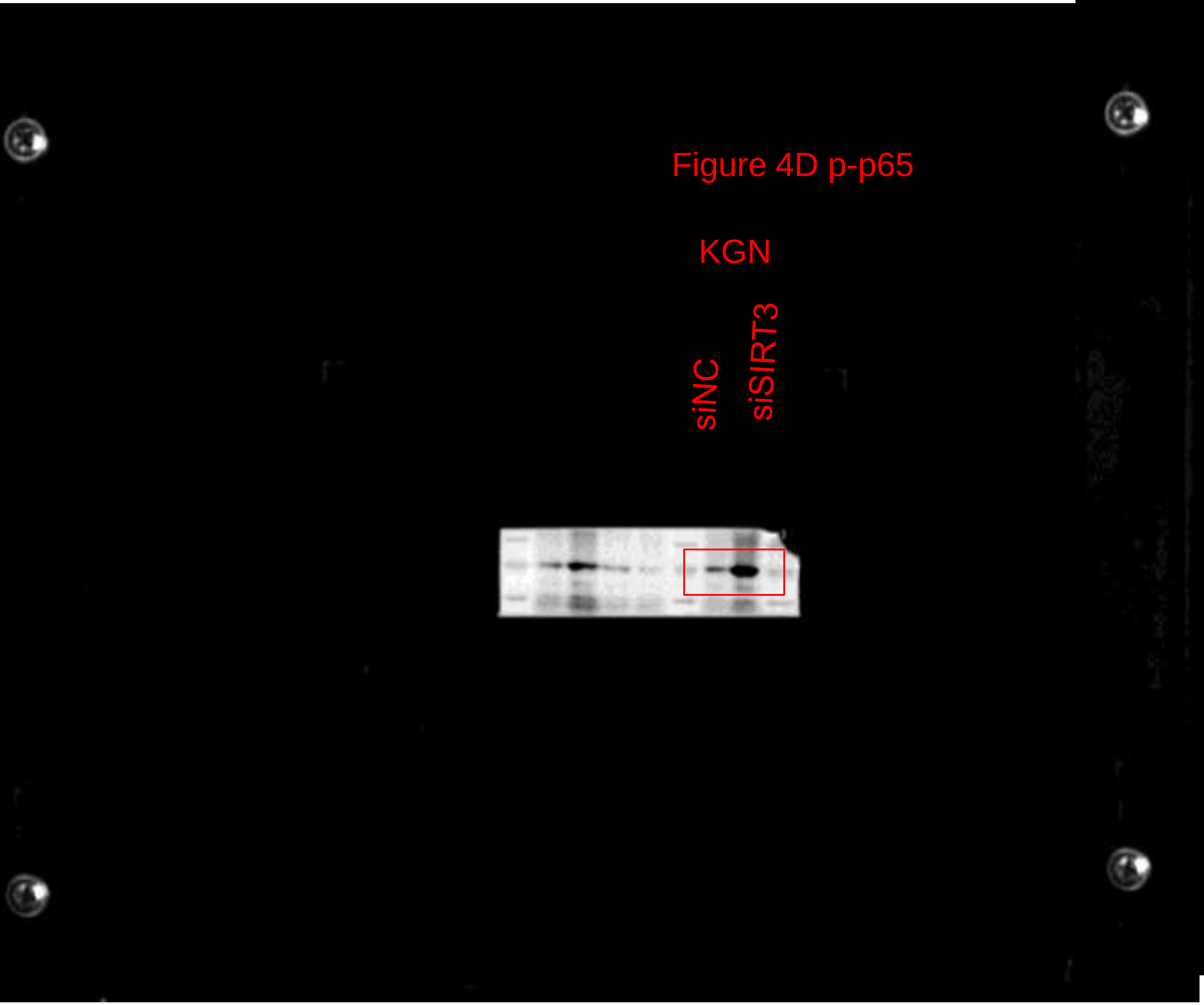
Figure 4D p-p65
KGN
siNC
siSIRT3

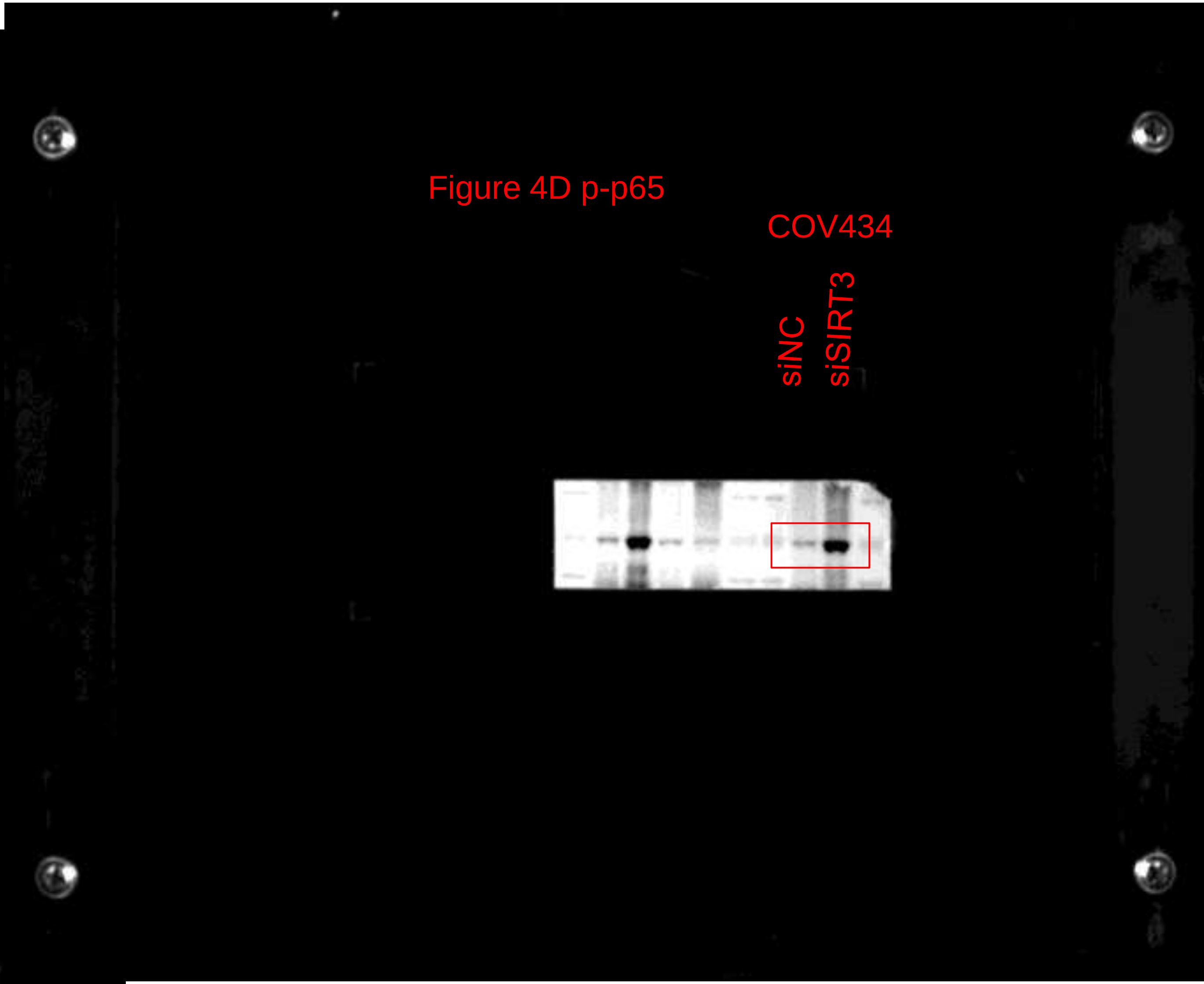
Figure 4D p-p65
COV434
siNC
siSIRT3

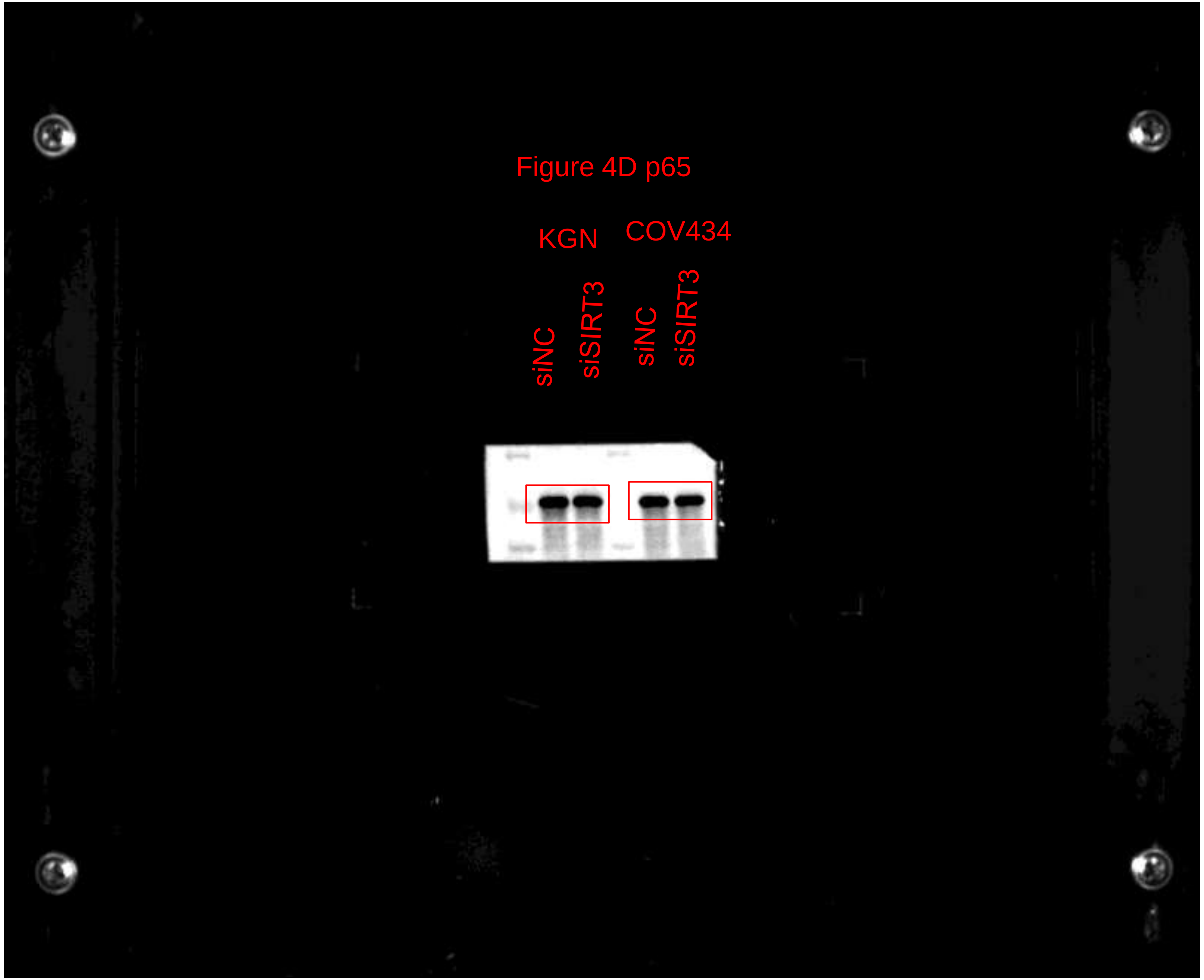
Figure 4D p65
KGN
COV434
siNC
siSIRT3
siNC
siSIRT3

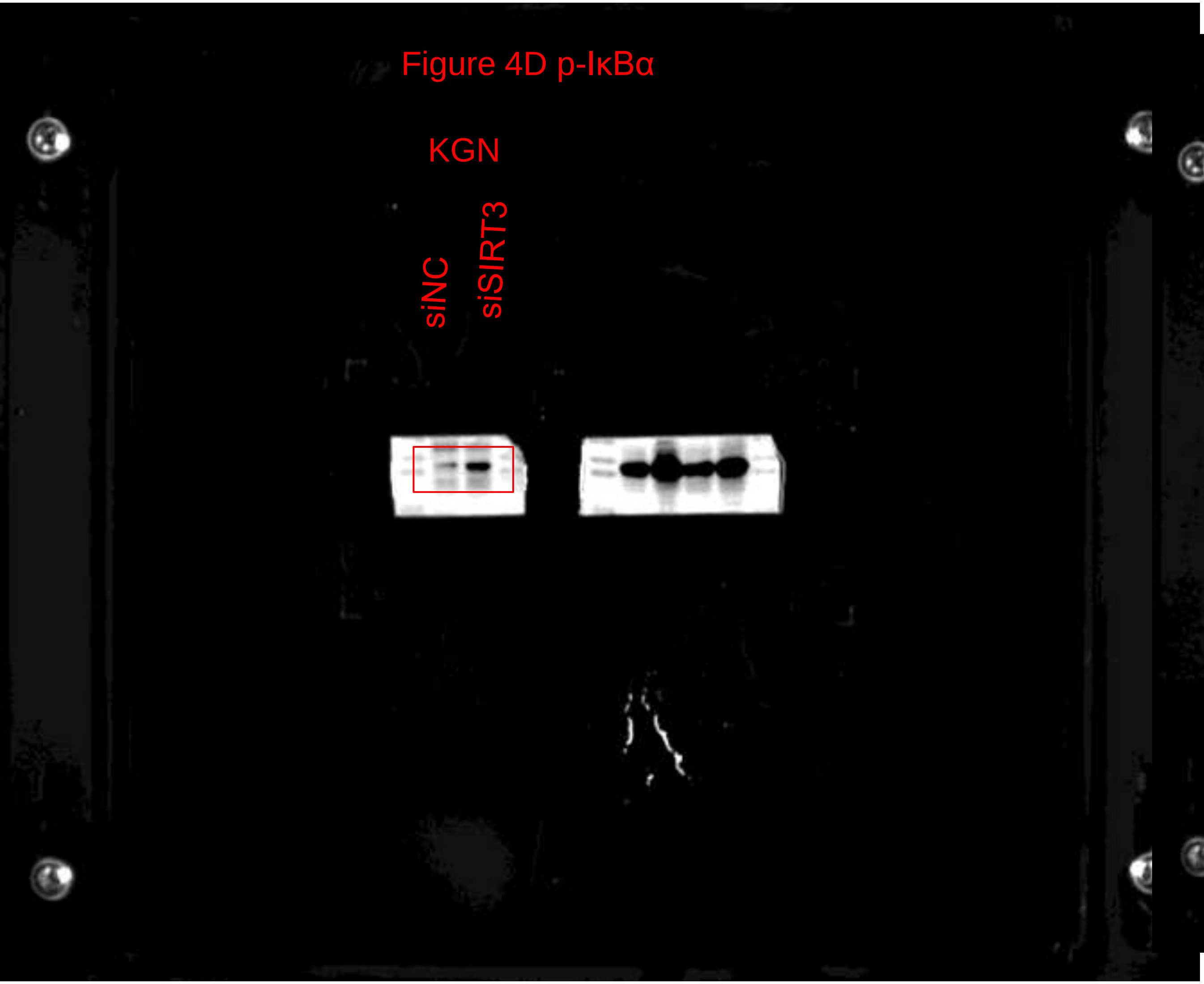
Figure 4D p-IκBα
KGN
siNC
siSIRT3

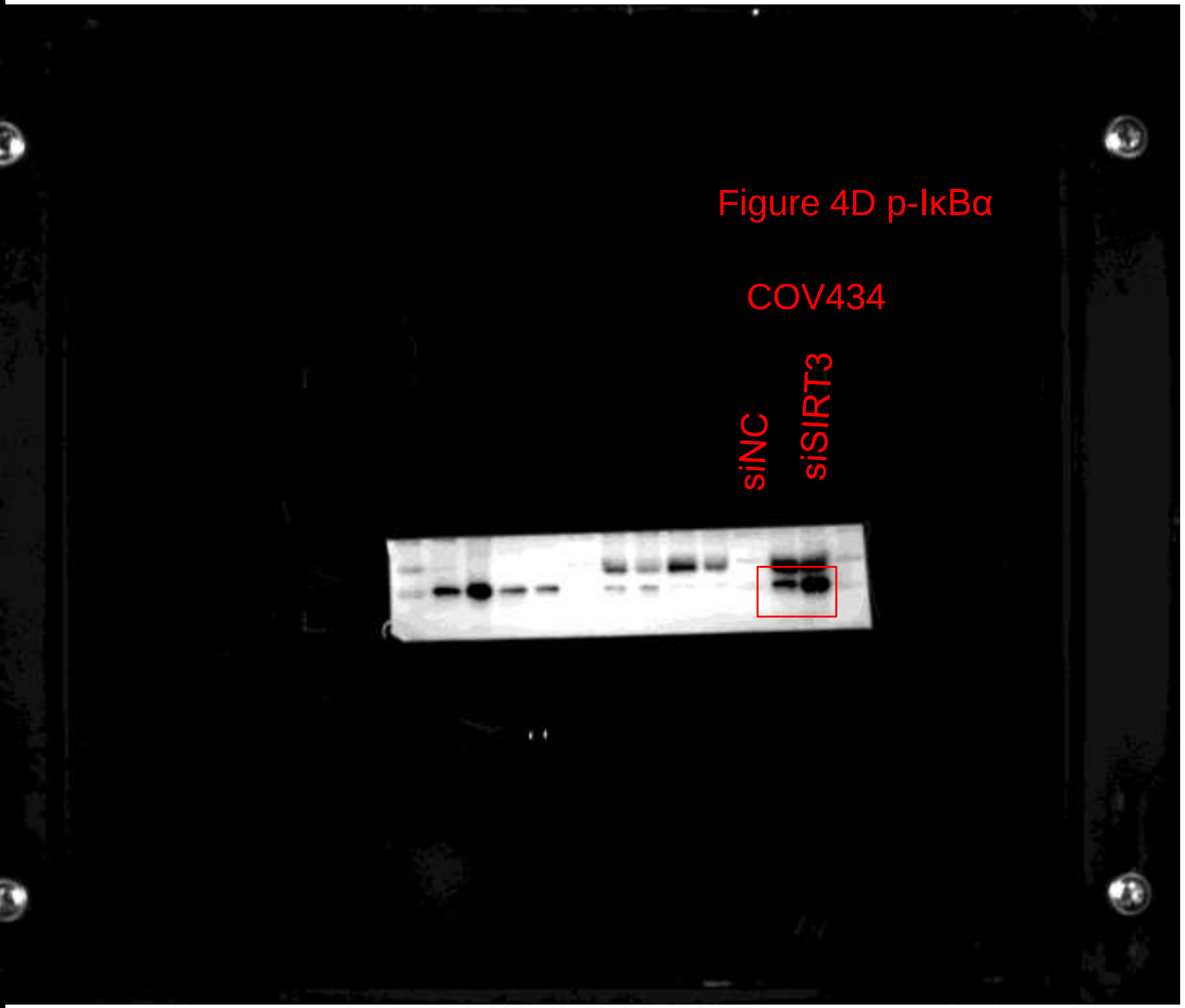
Figure 4D p-IκBα
COV434
siNC
siSIRT3

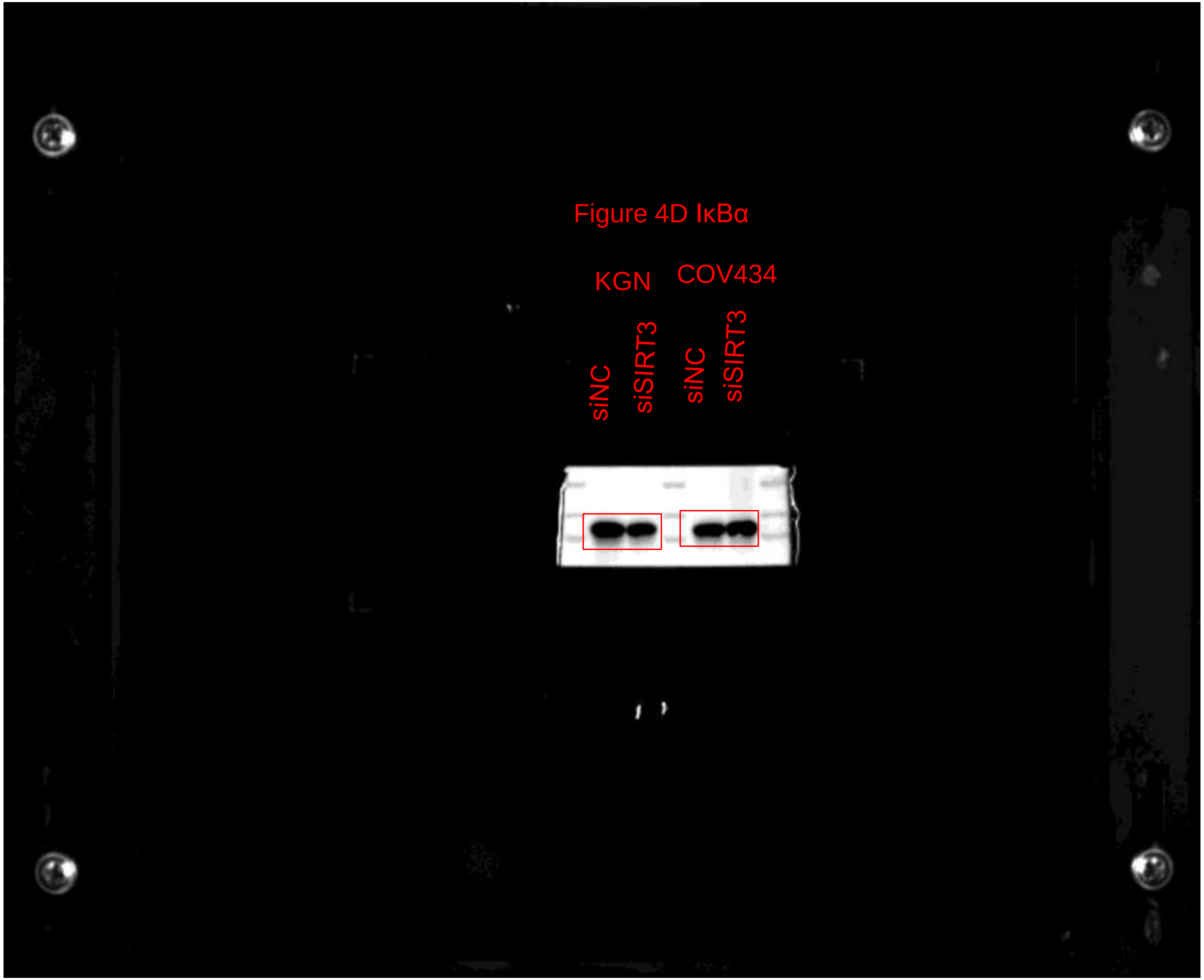
Figure 4D IκBα
KGN
COV434
siNC
siSIRT3
siNC
siSIRT3

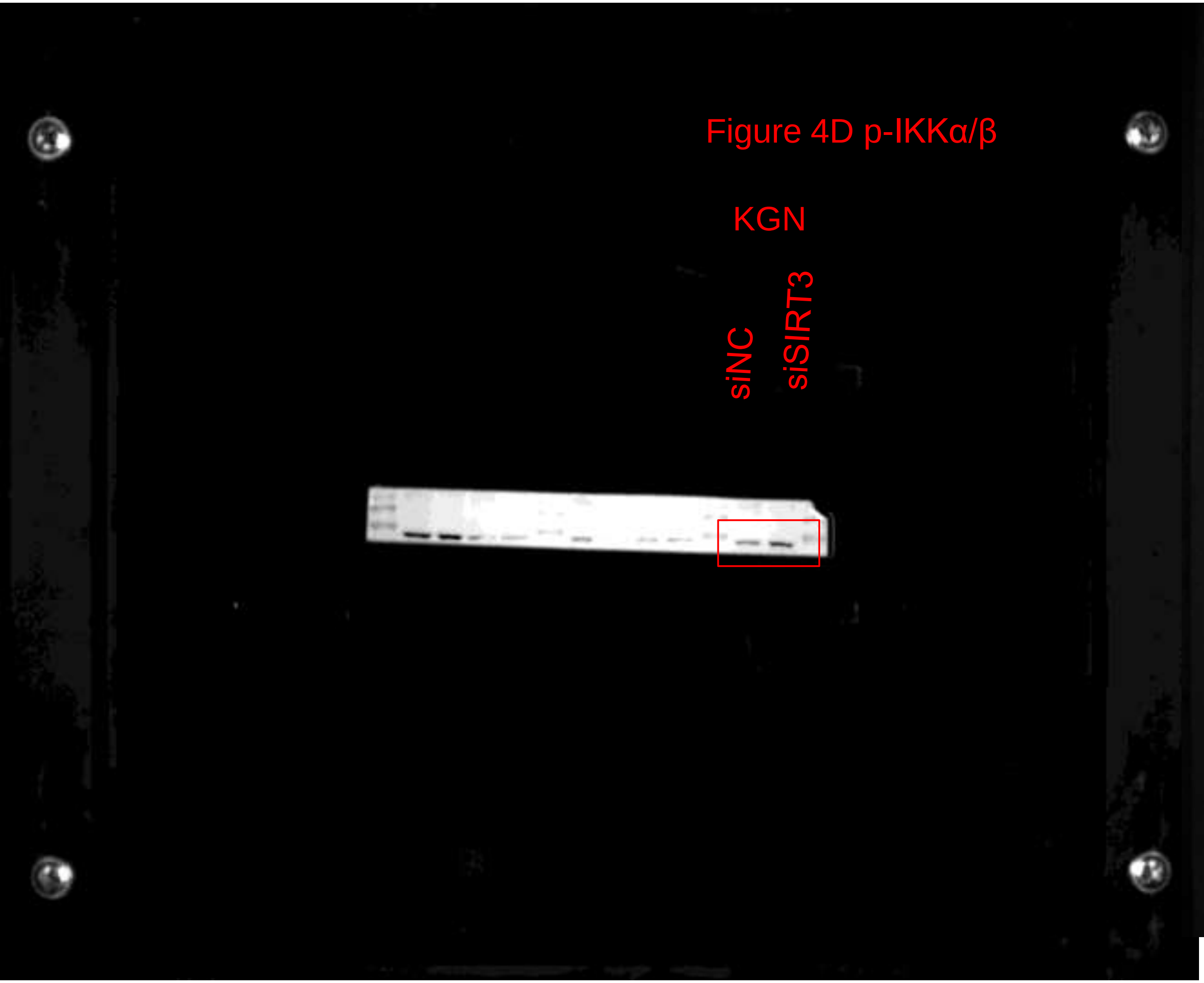
Figure 4D p-IKKα/β
KGN
siNC
siSIRT3

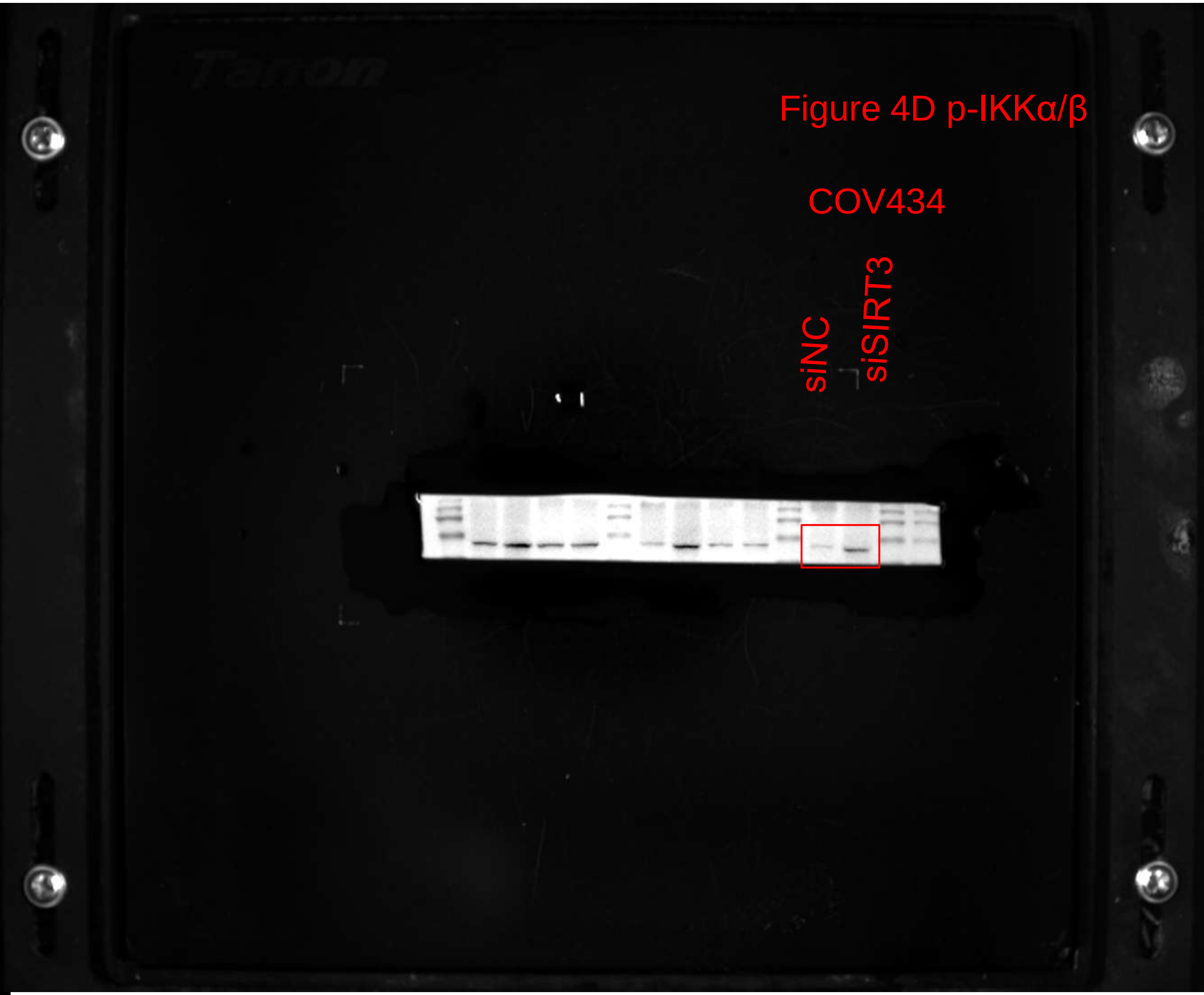
Figure 4D p-IKKα/β
COV434
siNC
siSIRT3

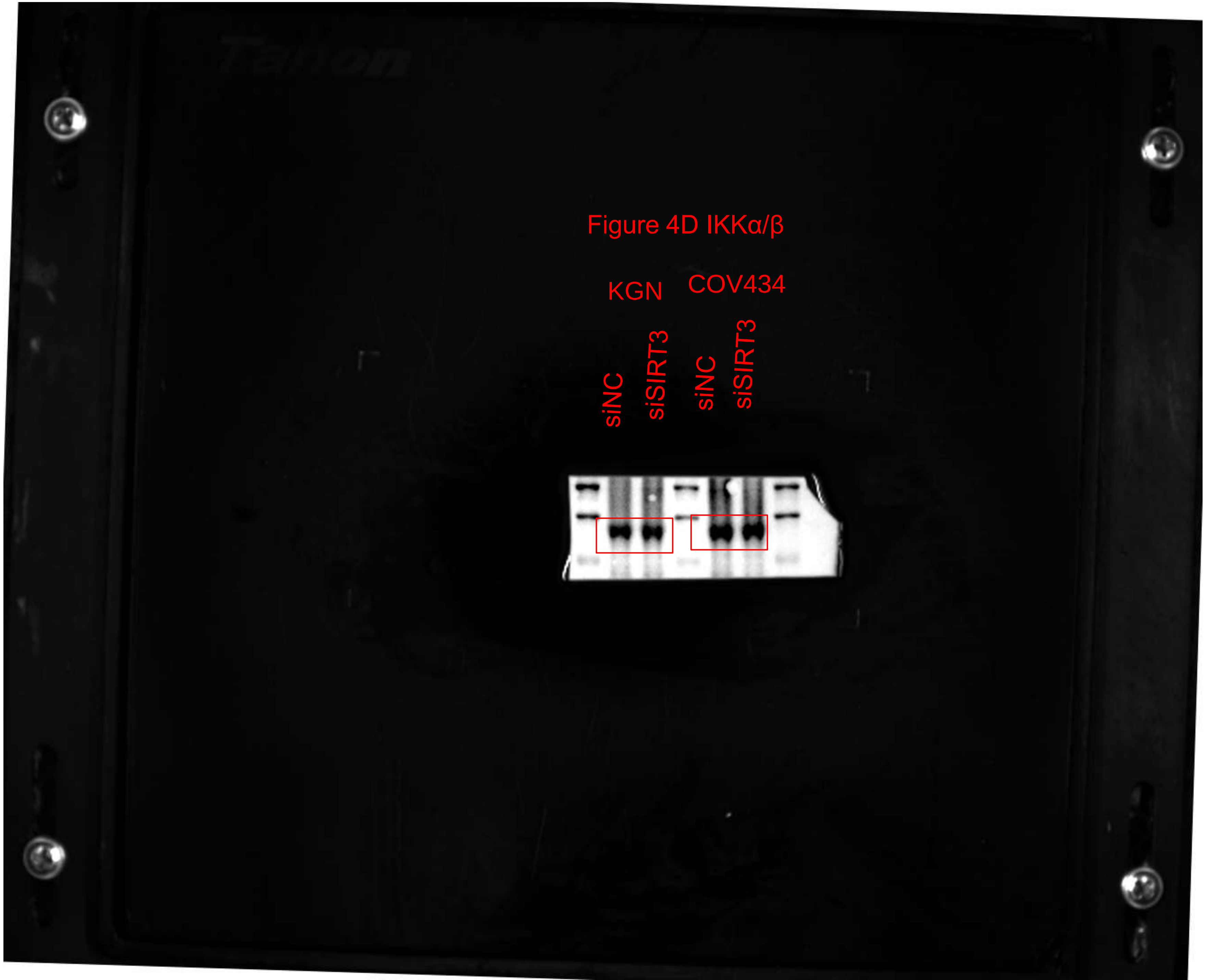
Figure 4D IKKα/β
KGN
COV434
siNC
siSIRT3
siNC
siSIRT3

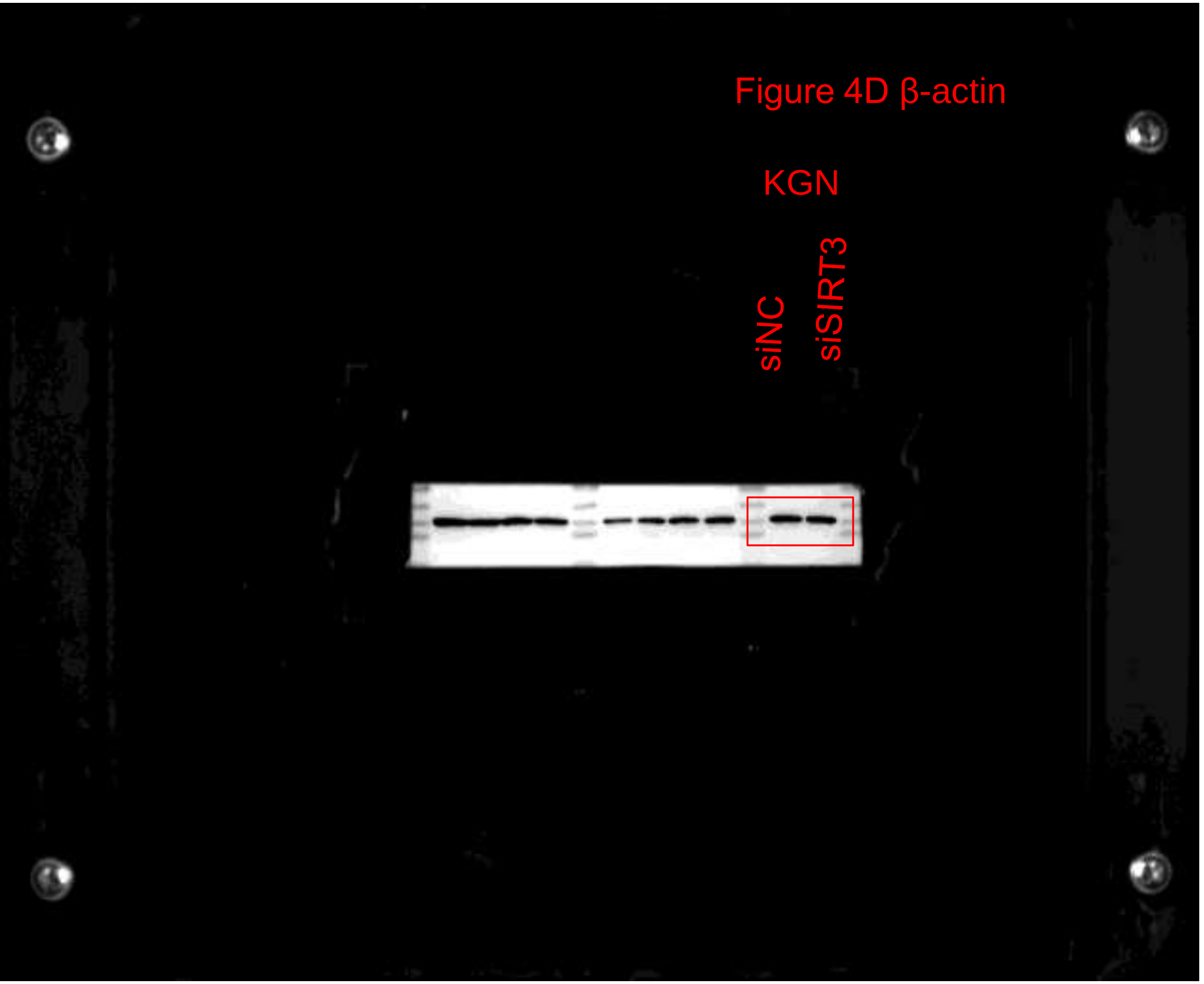
Figure 4D β-actin
KGN
siNC
siSIRT3

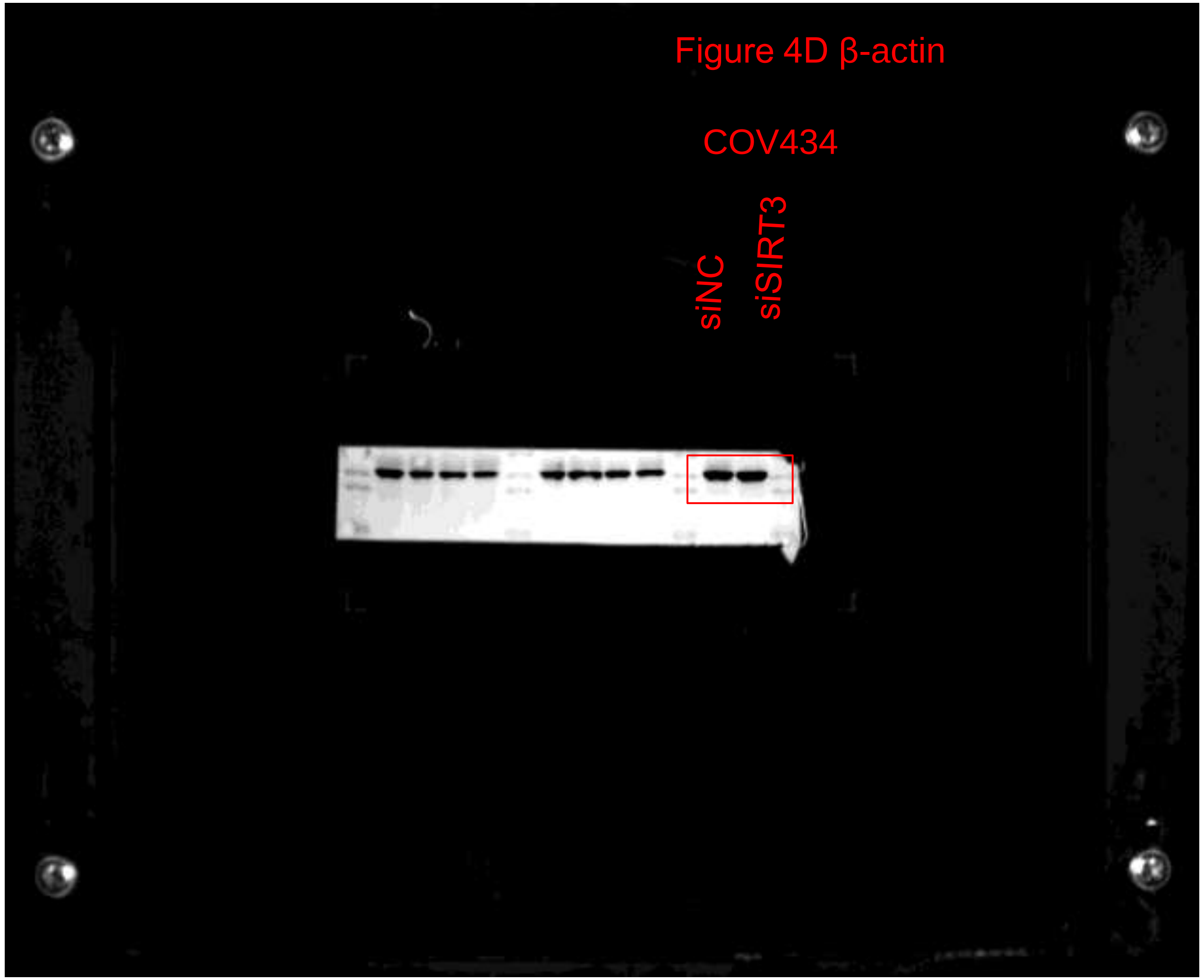
Figure 4D β-actin
COV434
siNC
siSIRT3

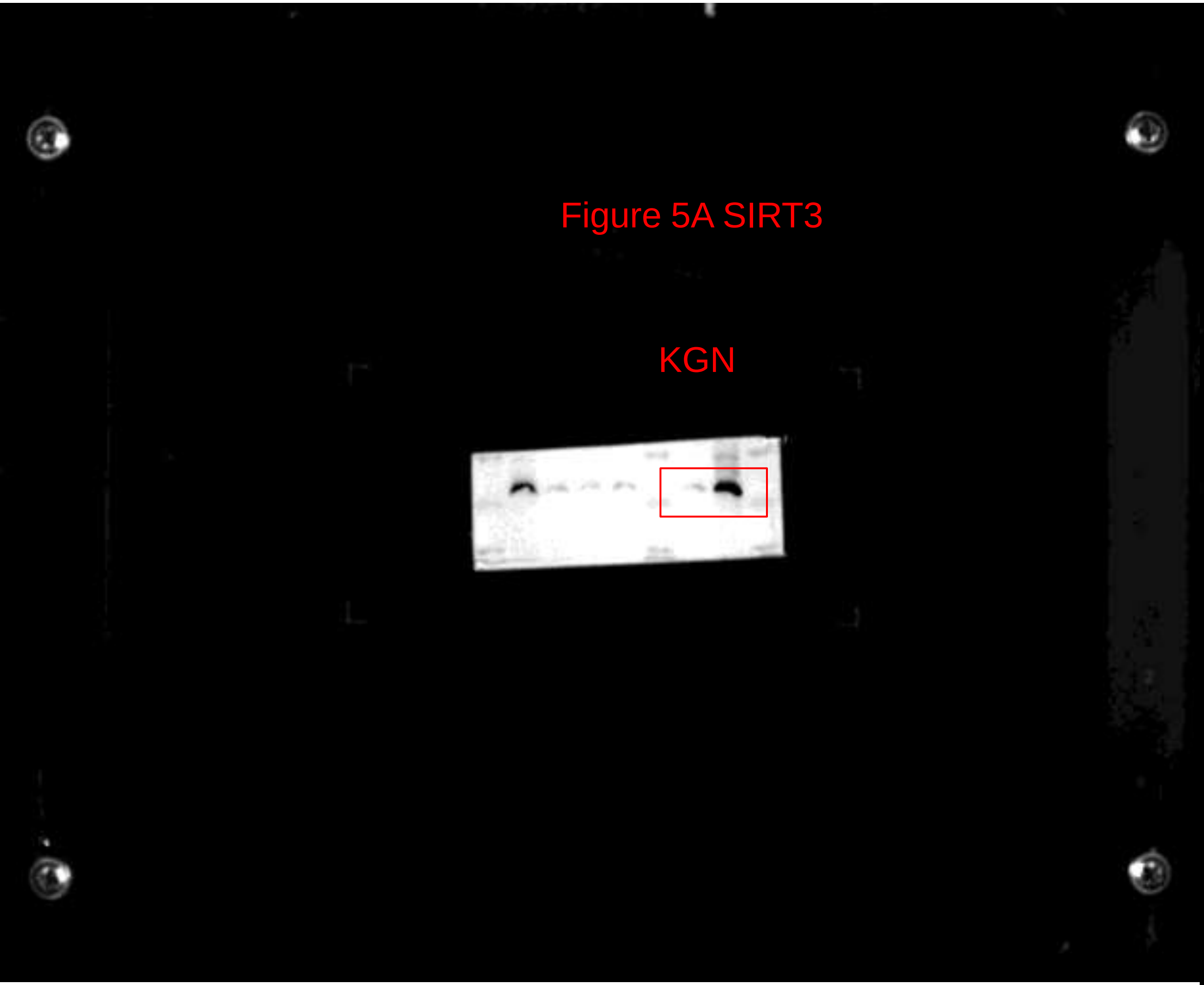
Figure 5A SIRT3
KGN

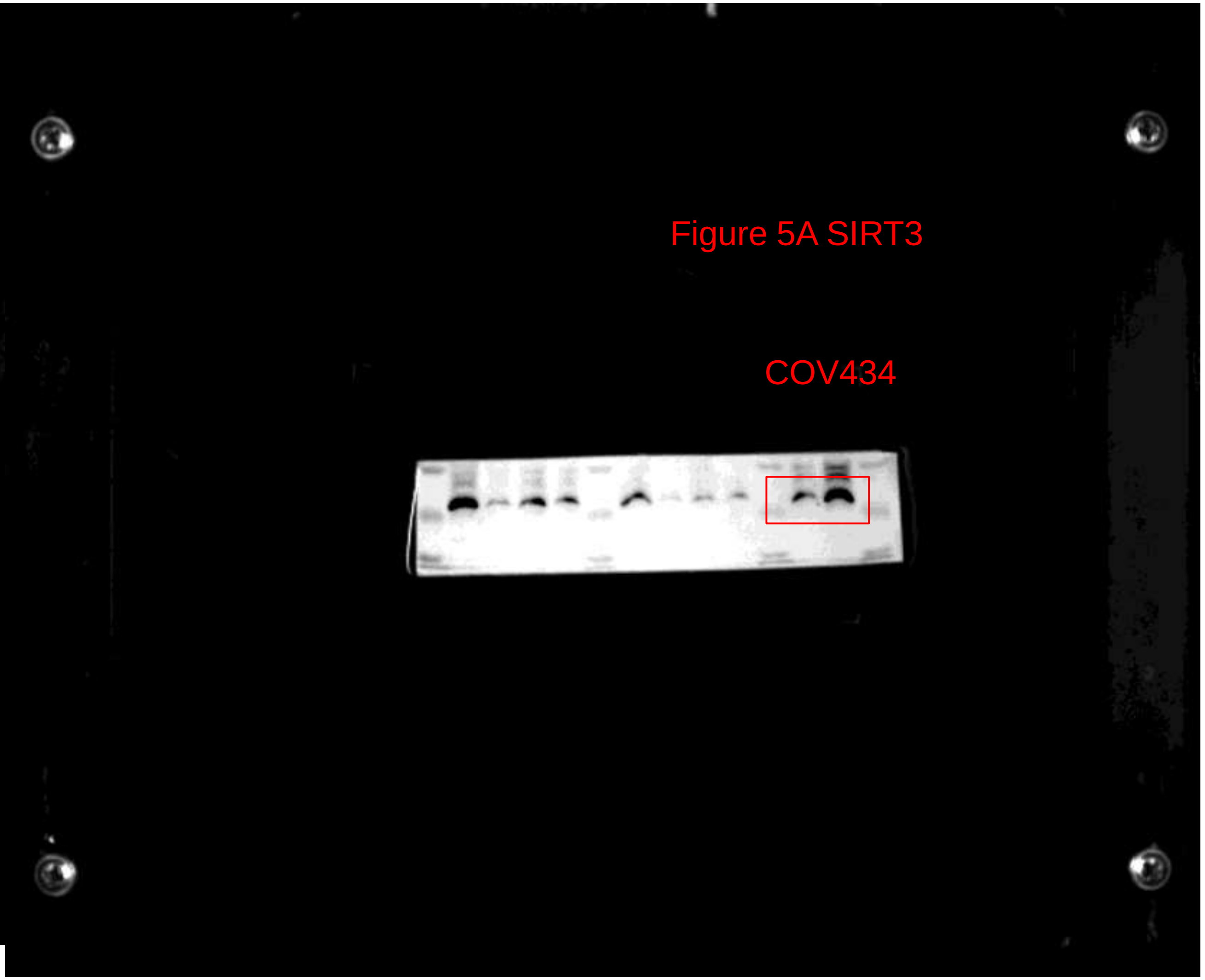
Figure 5A SIRT3
COV434

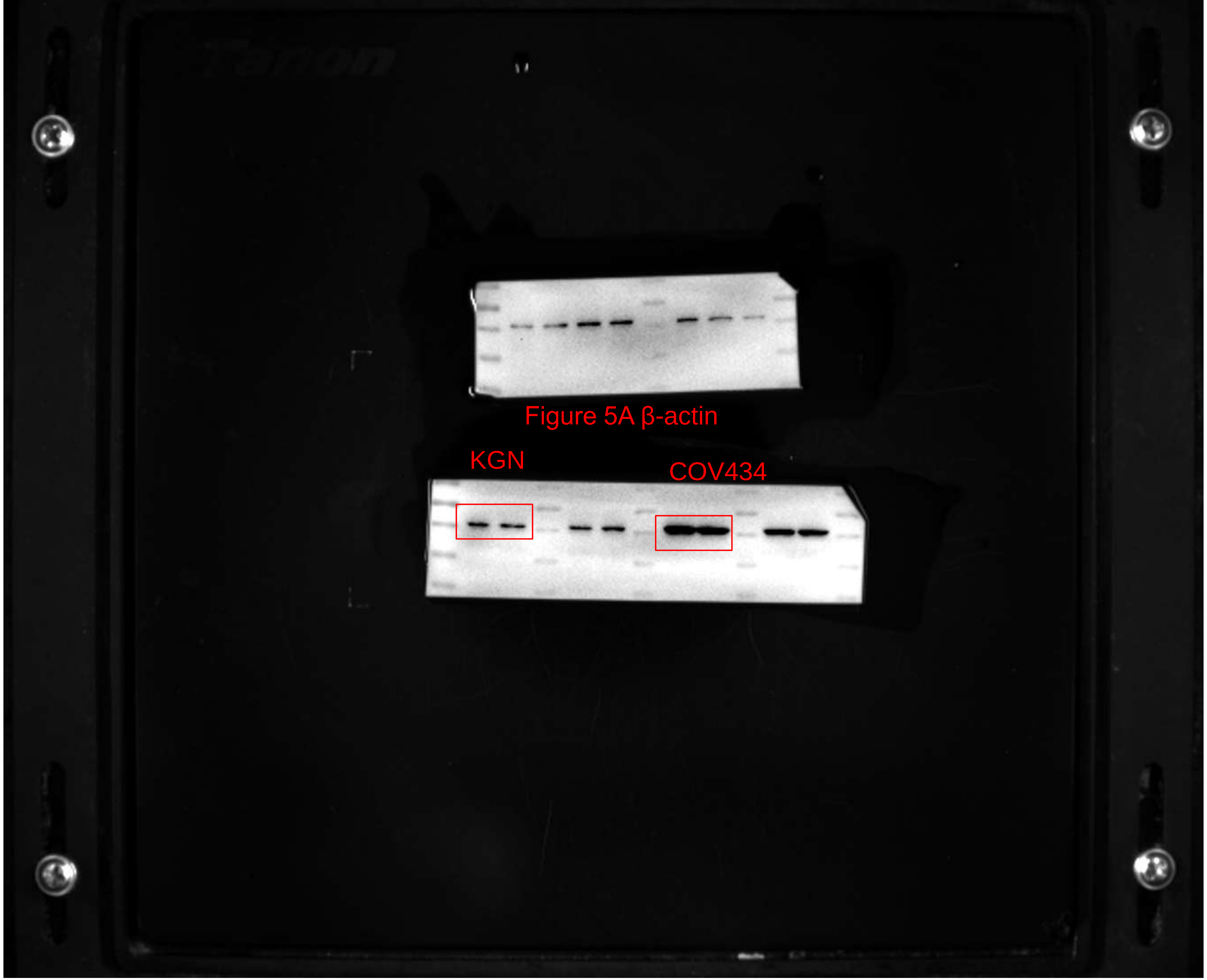
Figure 5A β-actin
KGN
COV434

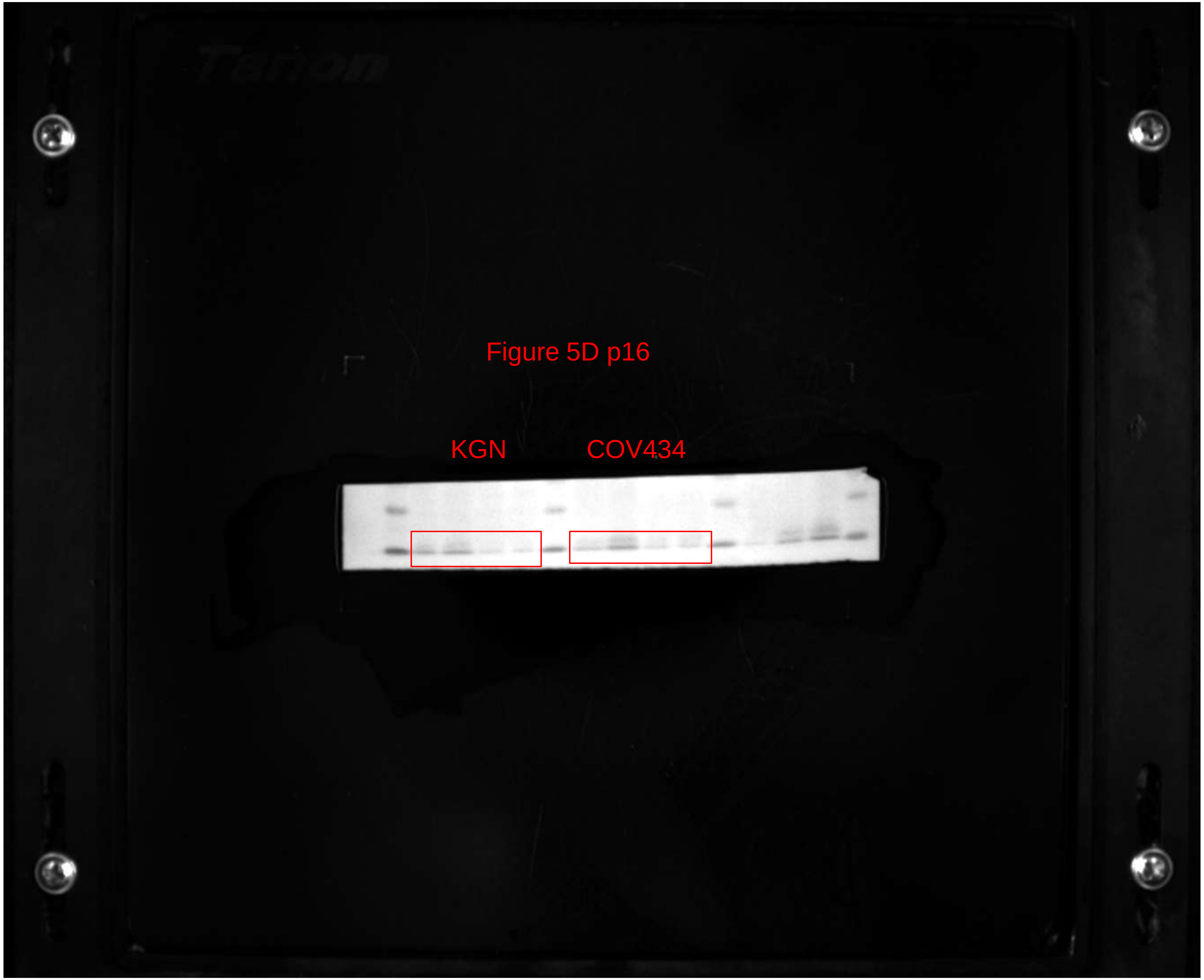
Figure 5D p16
KGN
COV434

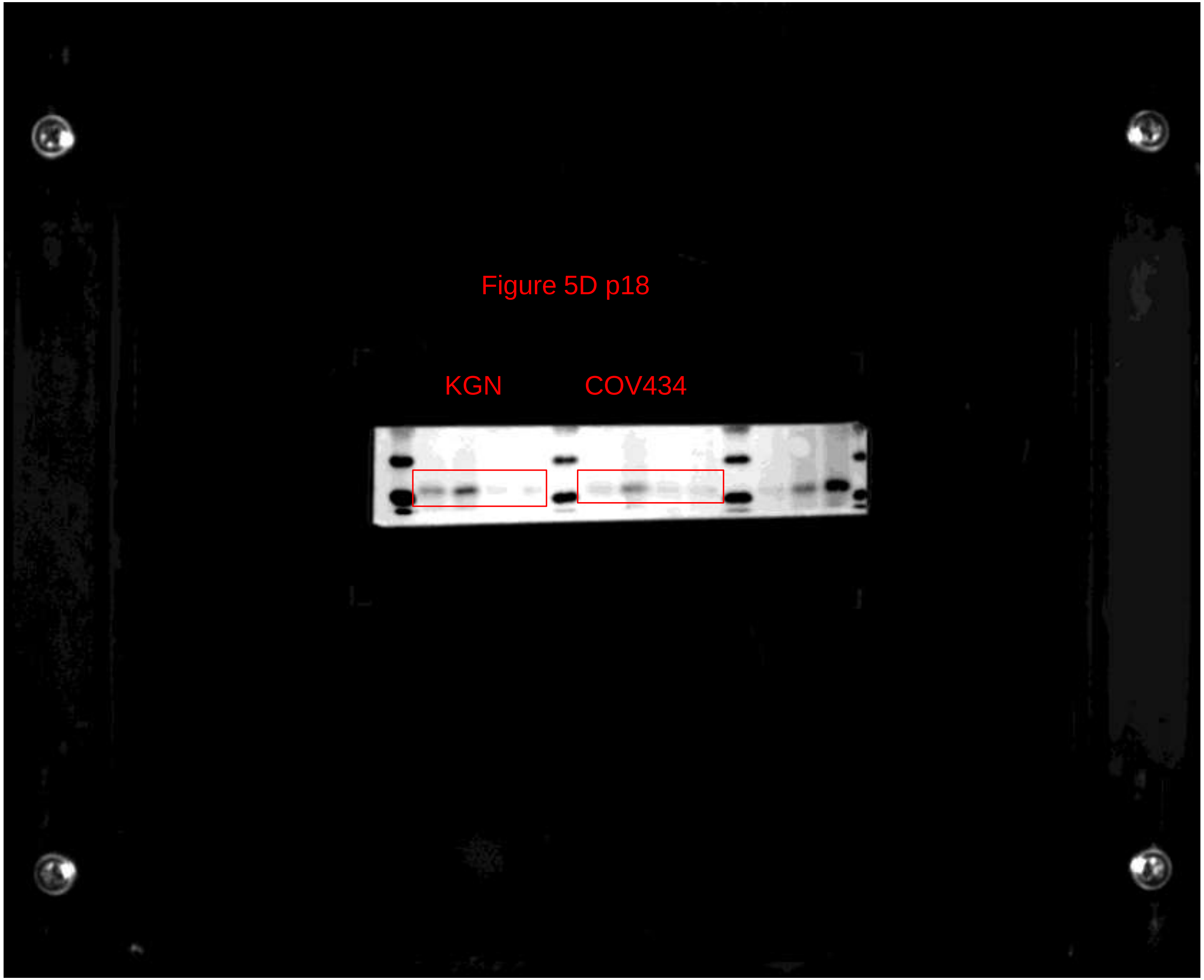
Figure 5D p18
KGN
COV434

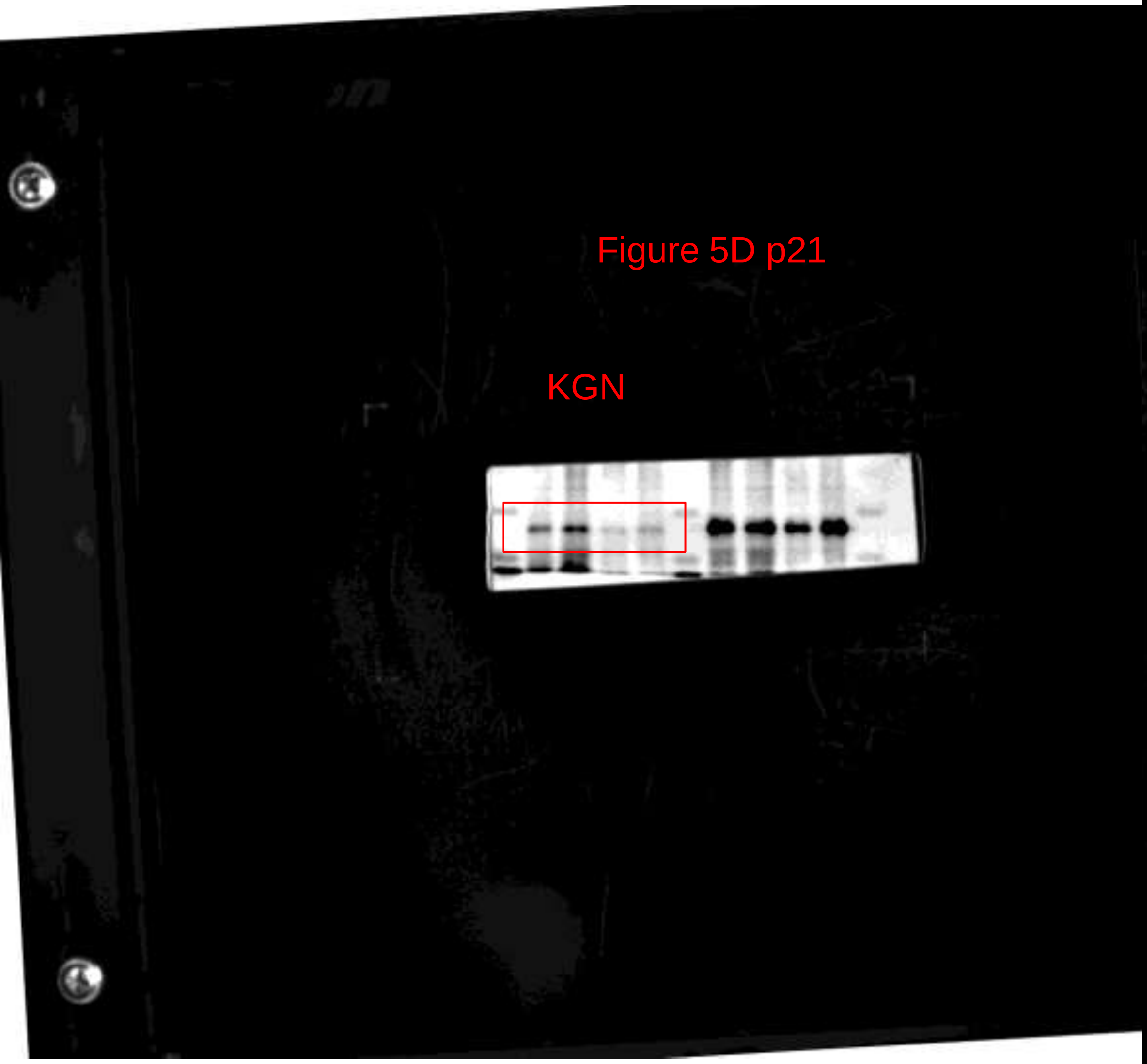
Figure 5D p21
KGN

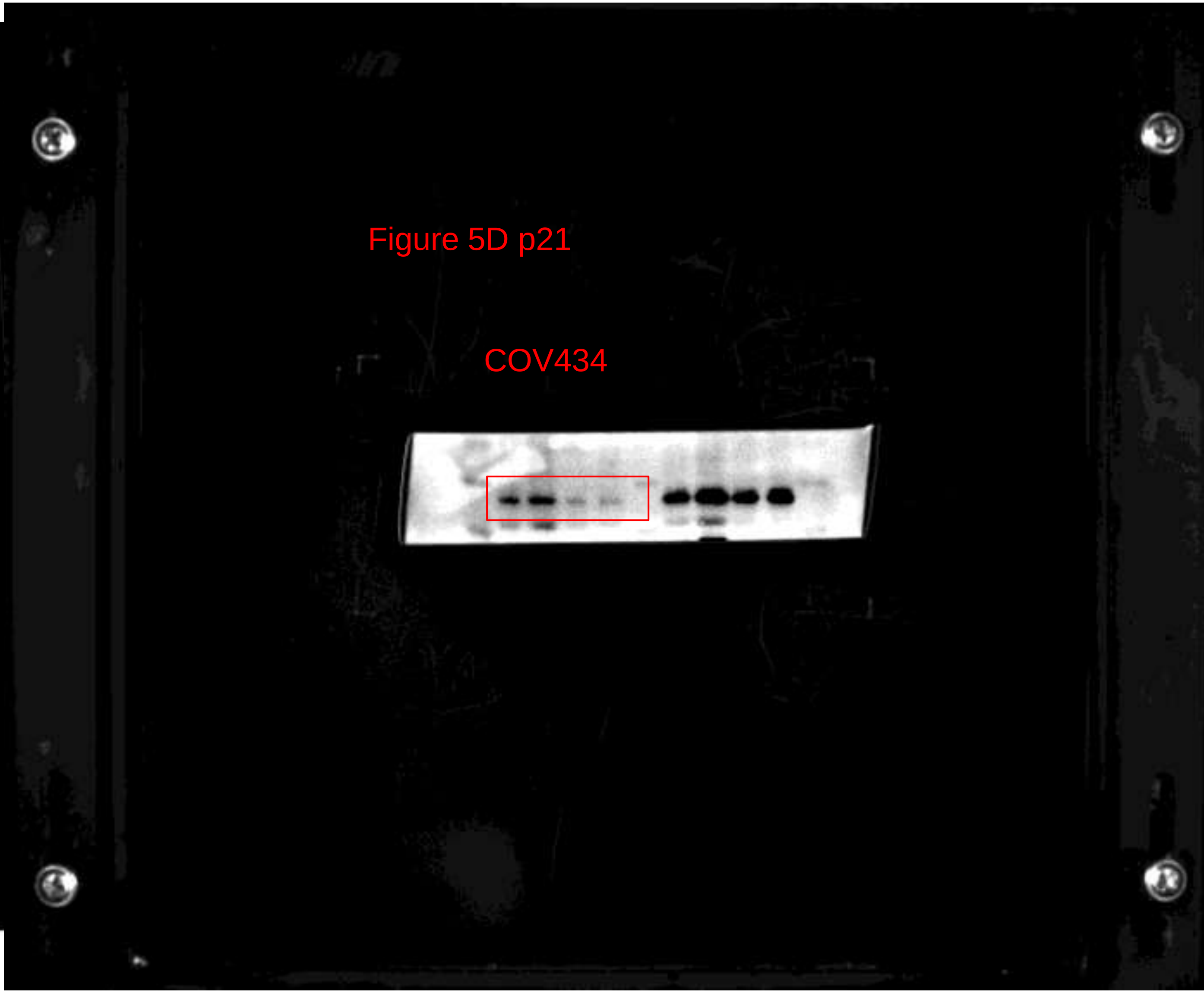
Figure 5D p21
COV434

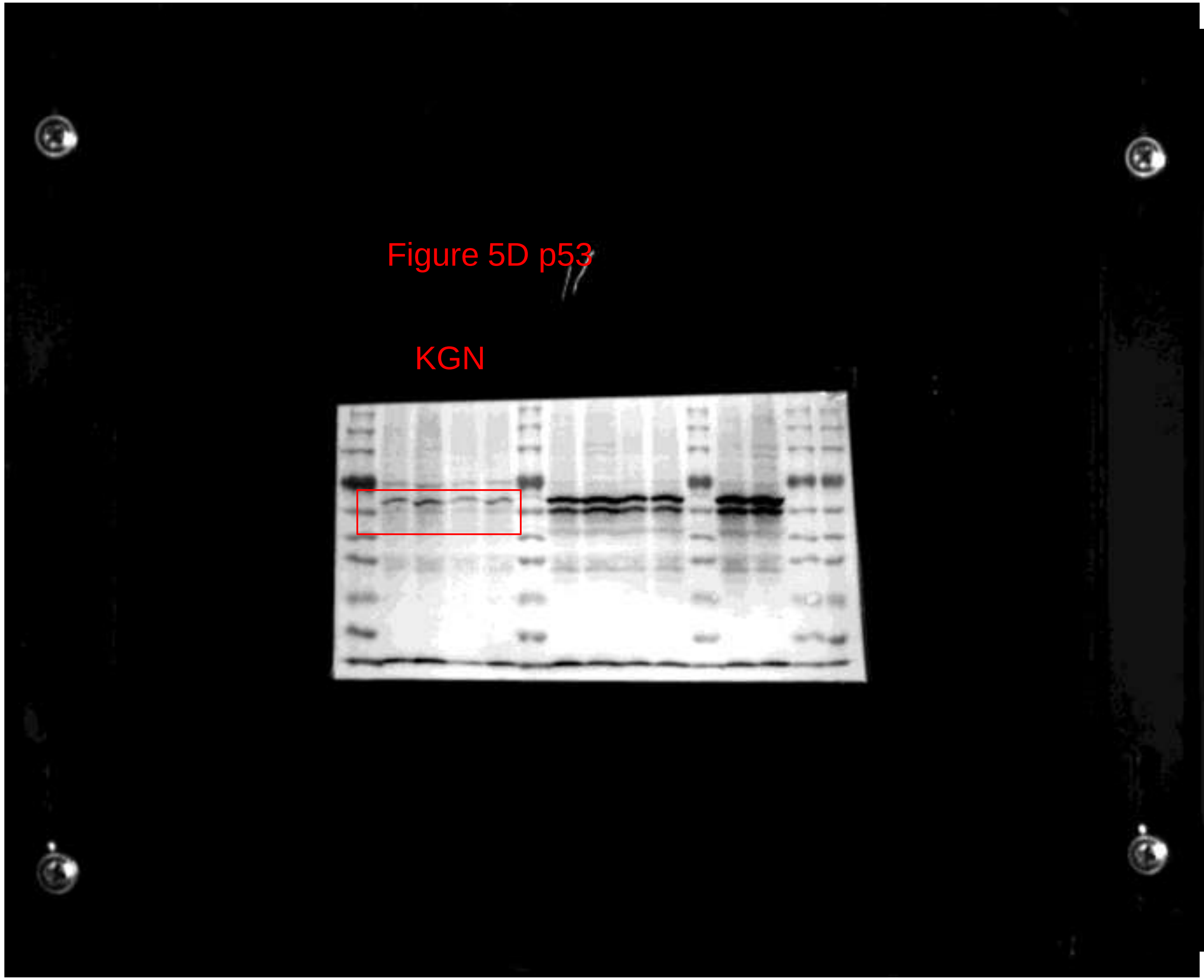
Figure 5D p53
KGN

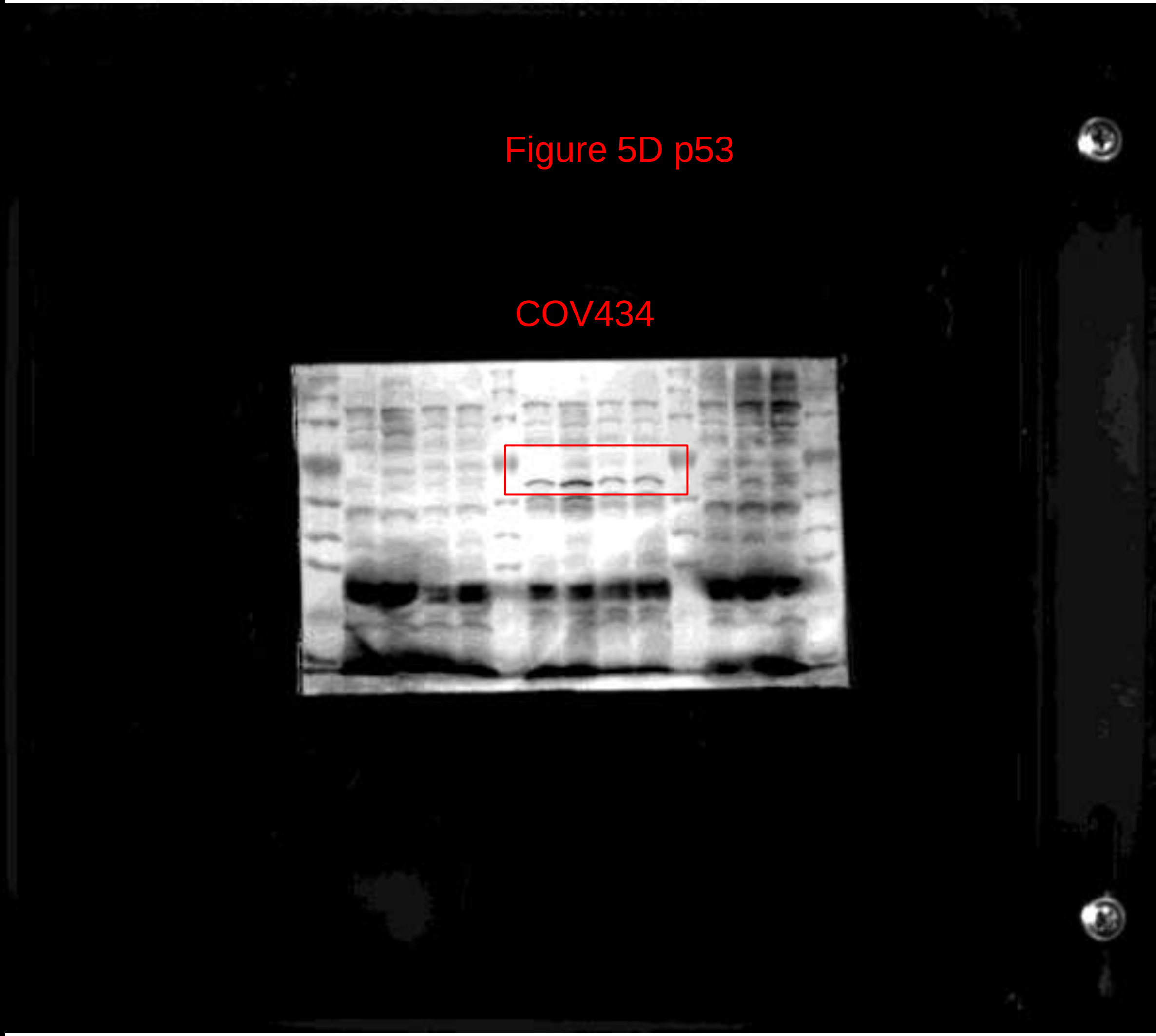
Figure 5D p53
COV434

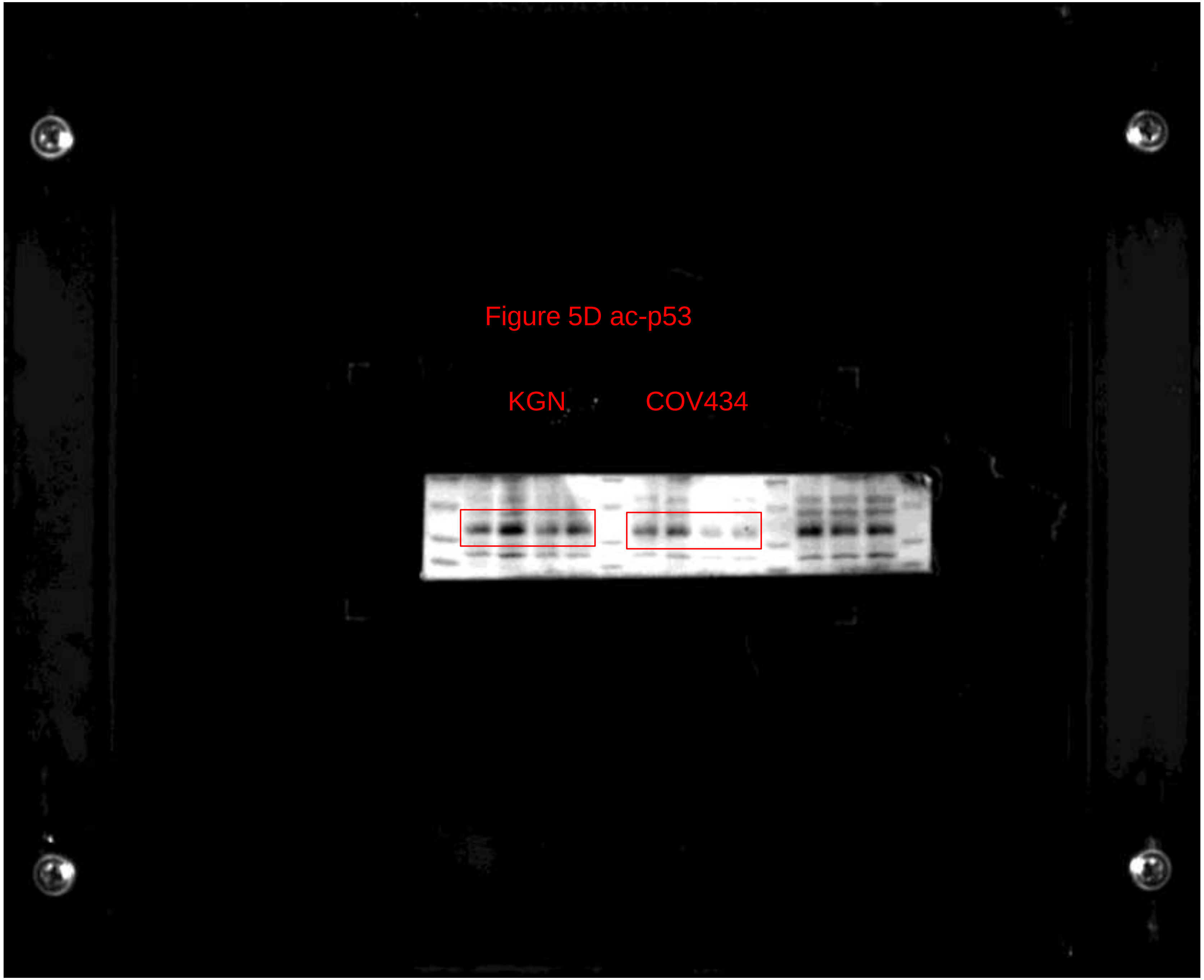
Figure 5D ac-p53
KGN
COV434

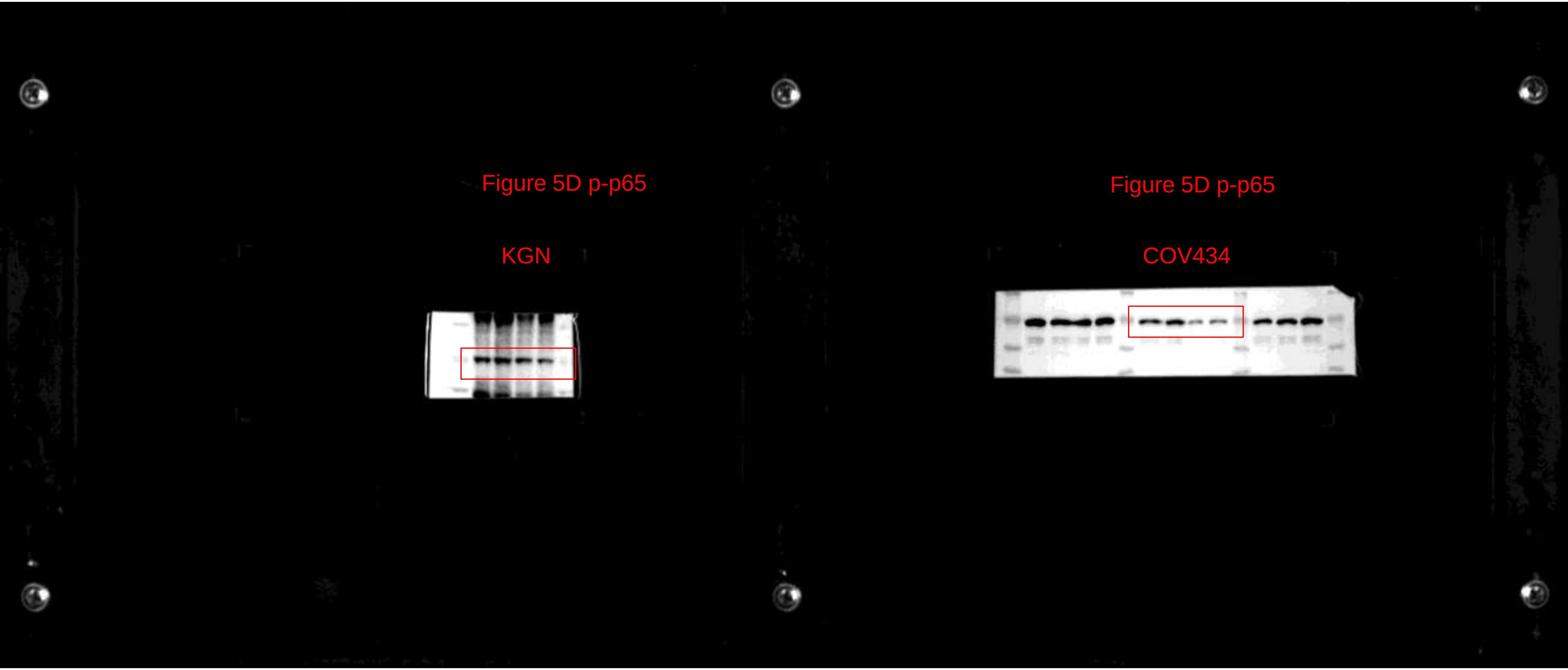
Figure 5D p-p65
KGN
Figure 5D p-p65
COV434

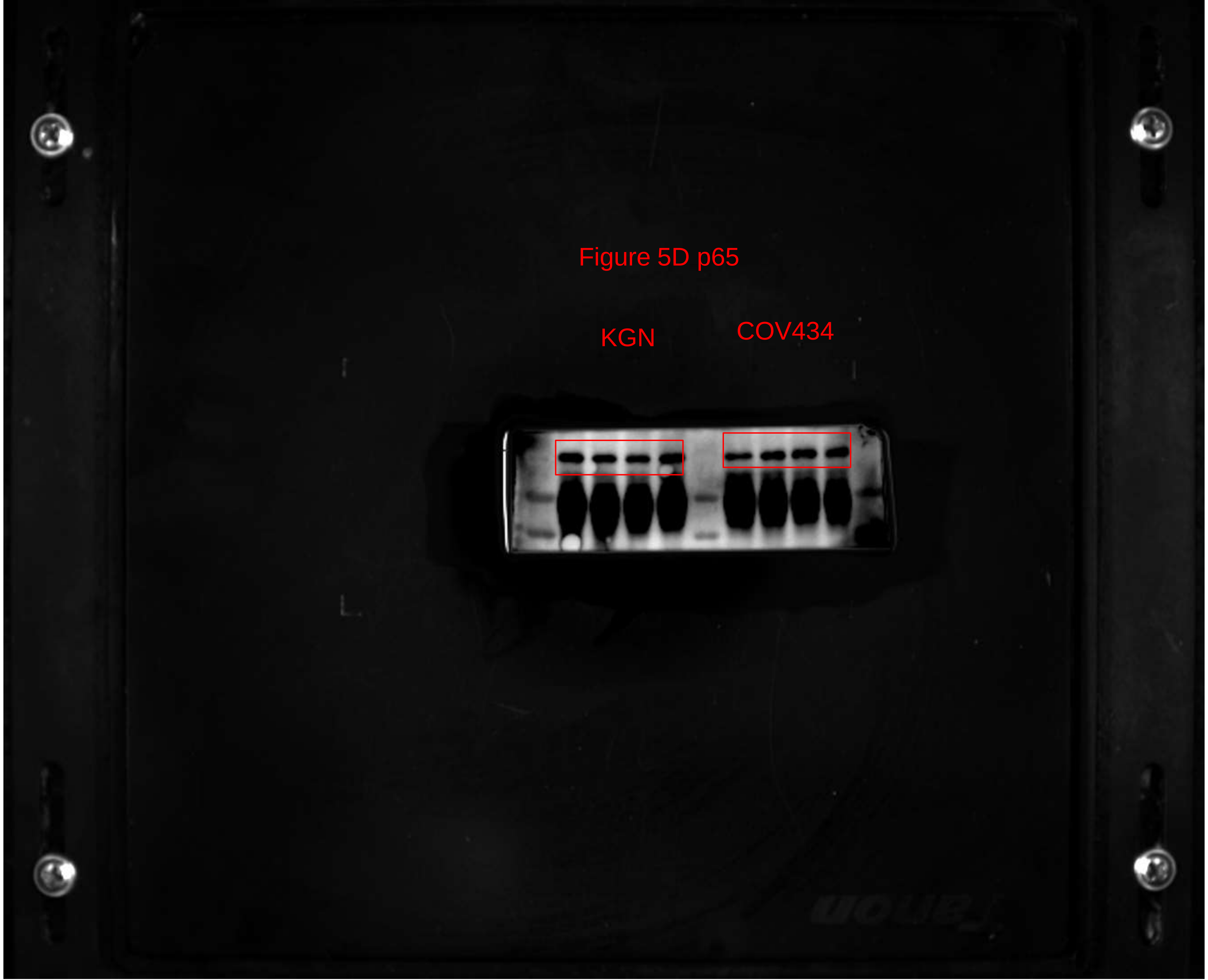
Figure 5D p65
KGN
COV434

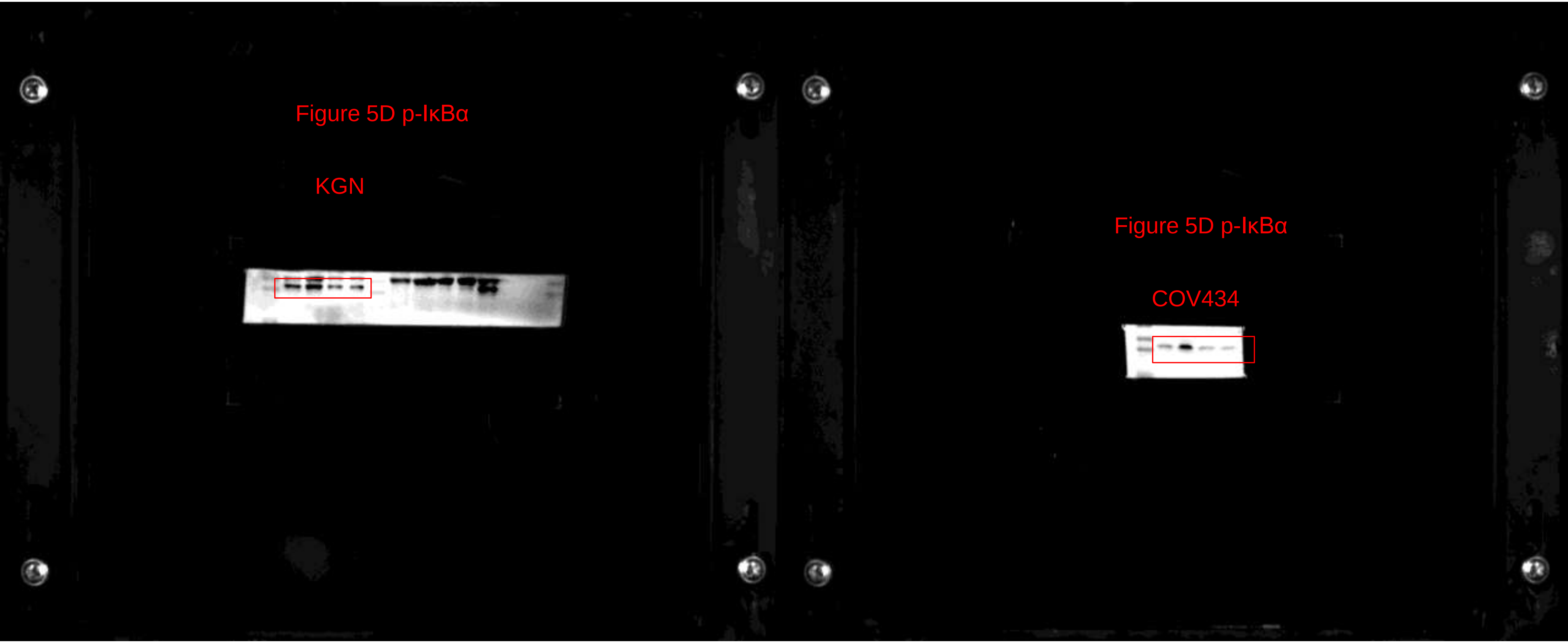
Figure 5D p-IκBα
KGN
Figure 5D p-IκBα
COV434

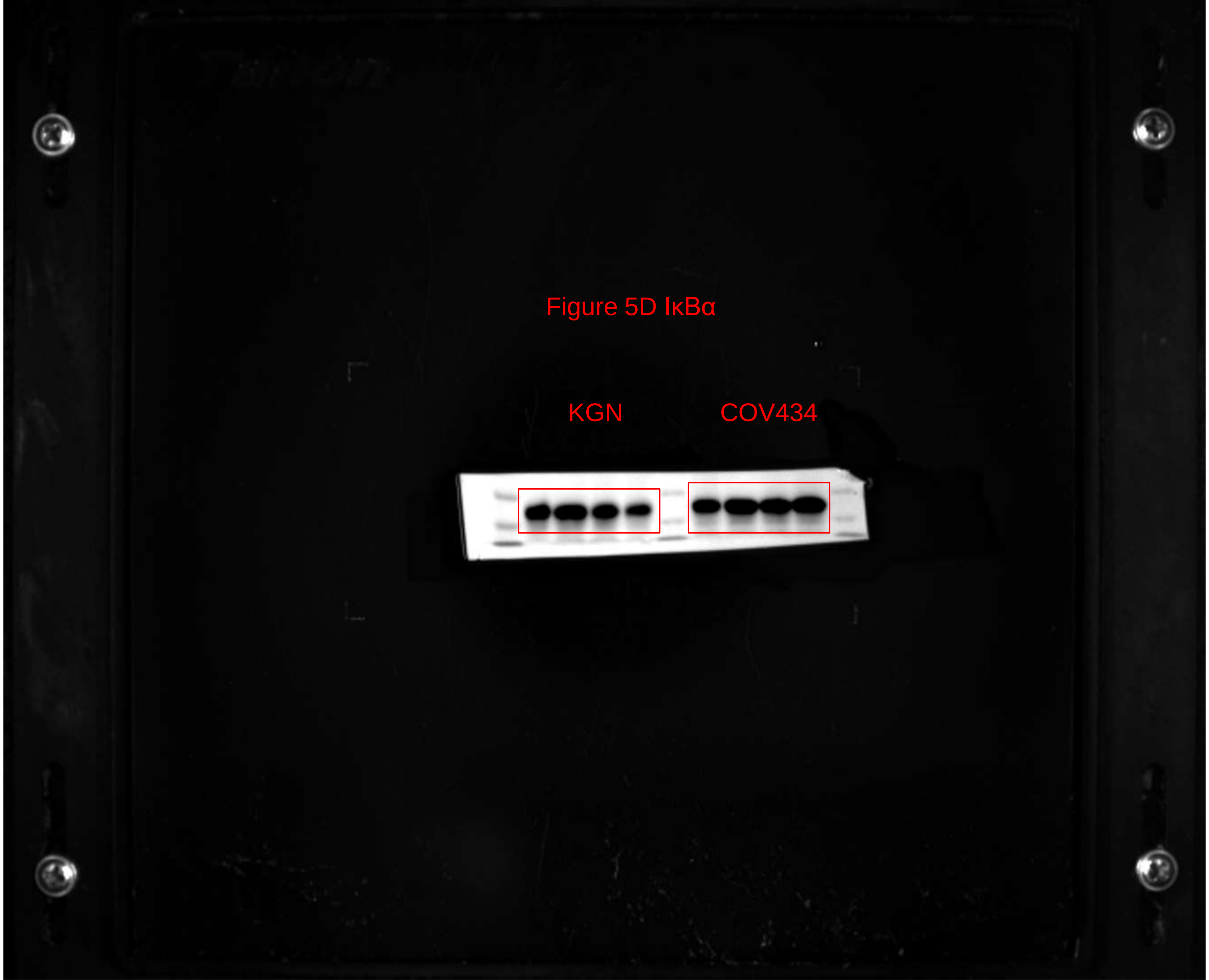
Figure 5D IκBα
KGN
COV434

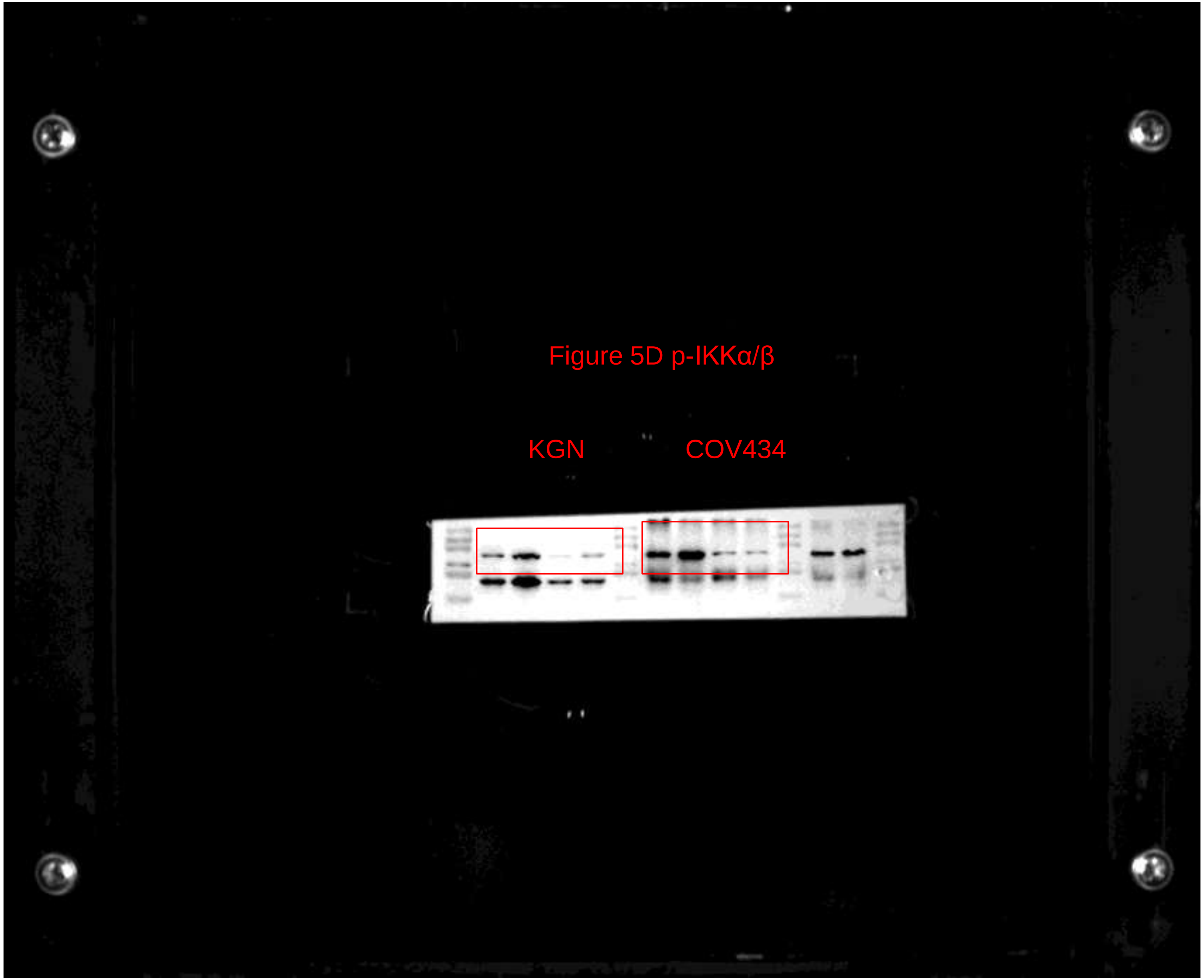
Figure 5D p-IKKα/β
KGN
COV434

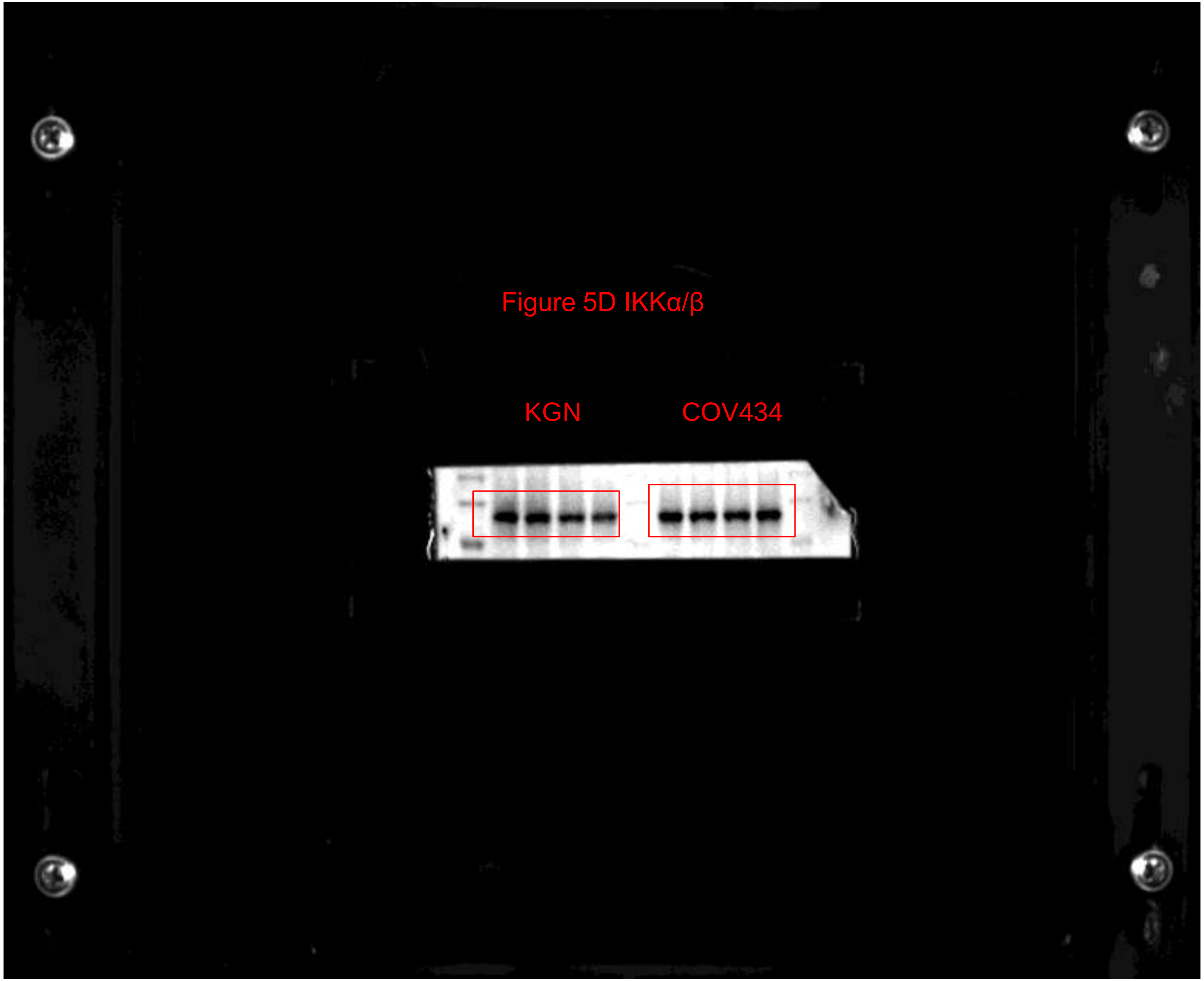
Figure 5D IKKα/β
KGN
COV434

Figure 5D β-actin

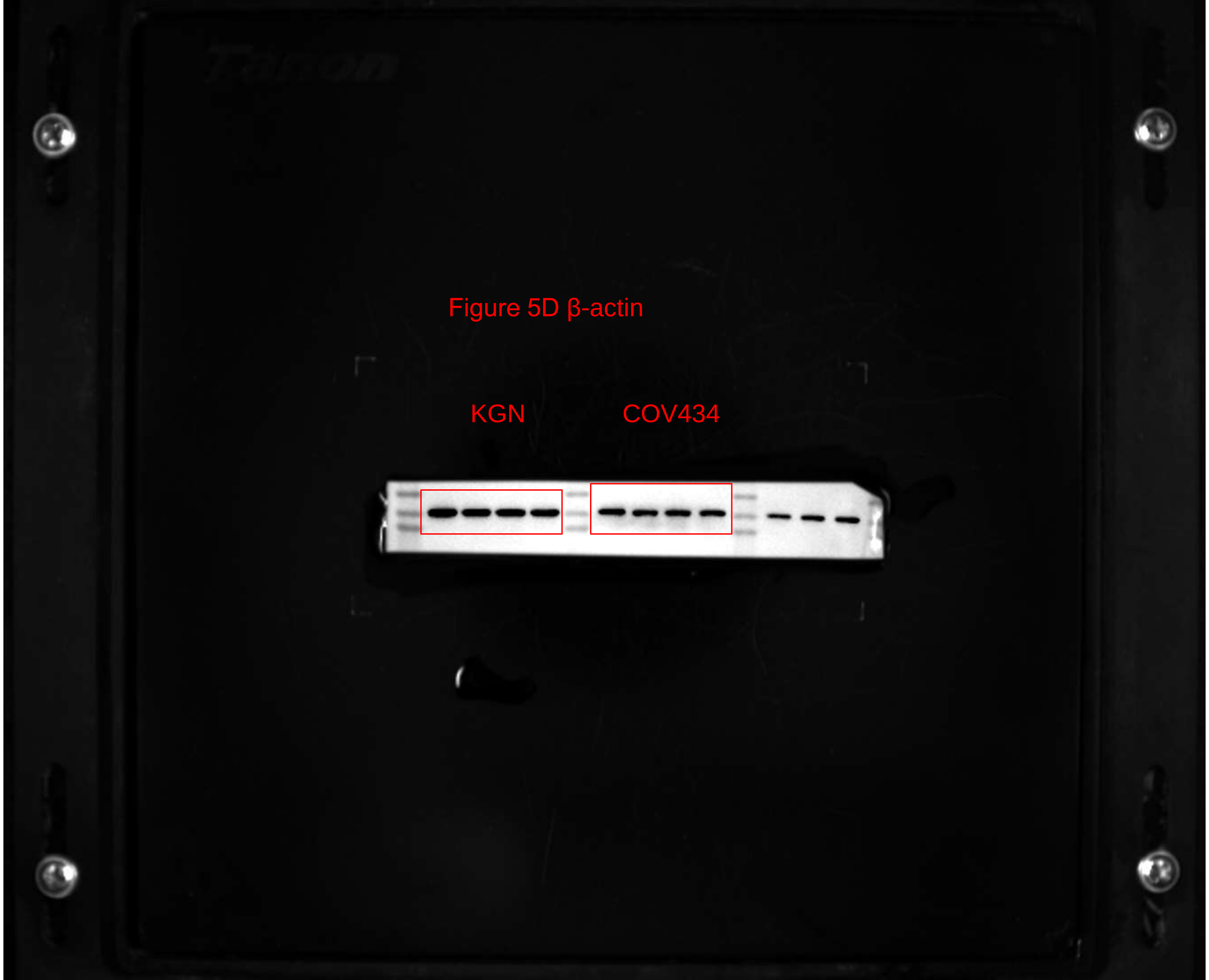

Figure 6A

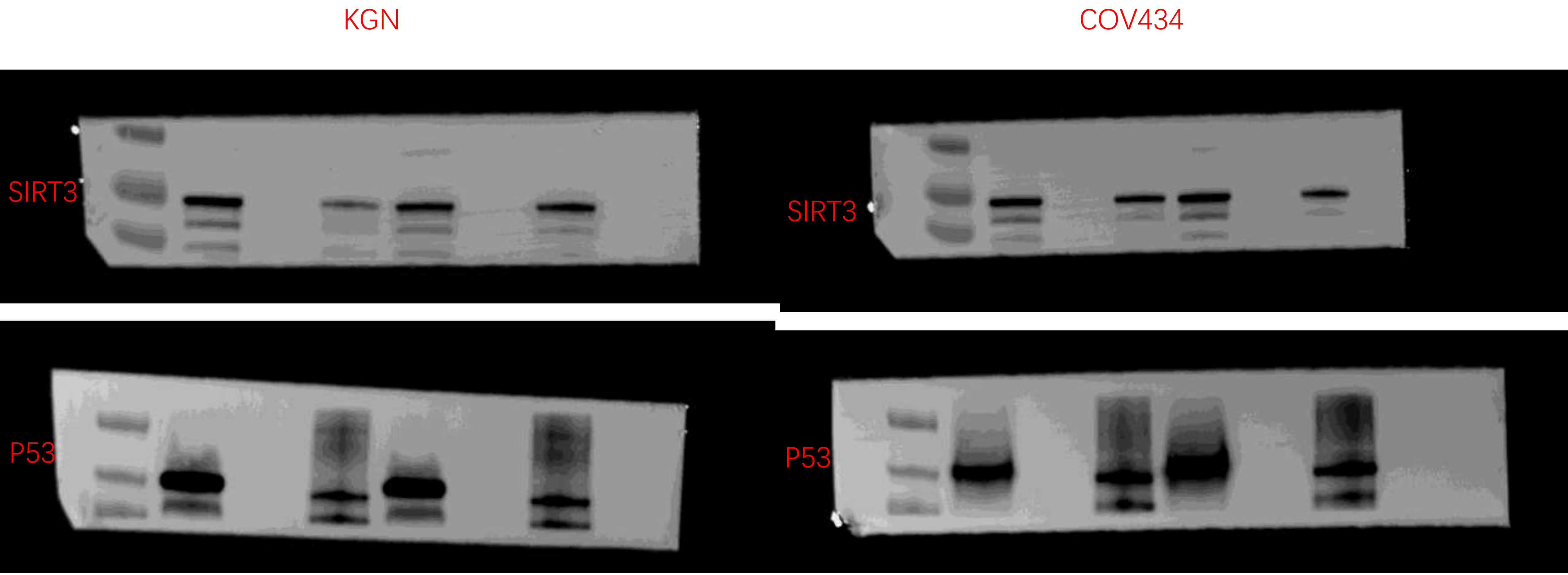

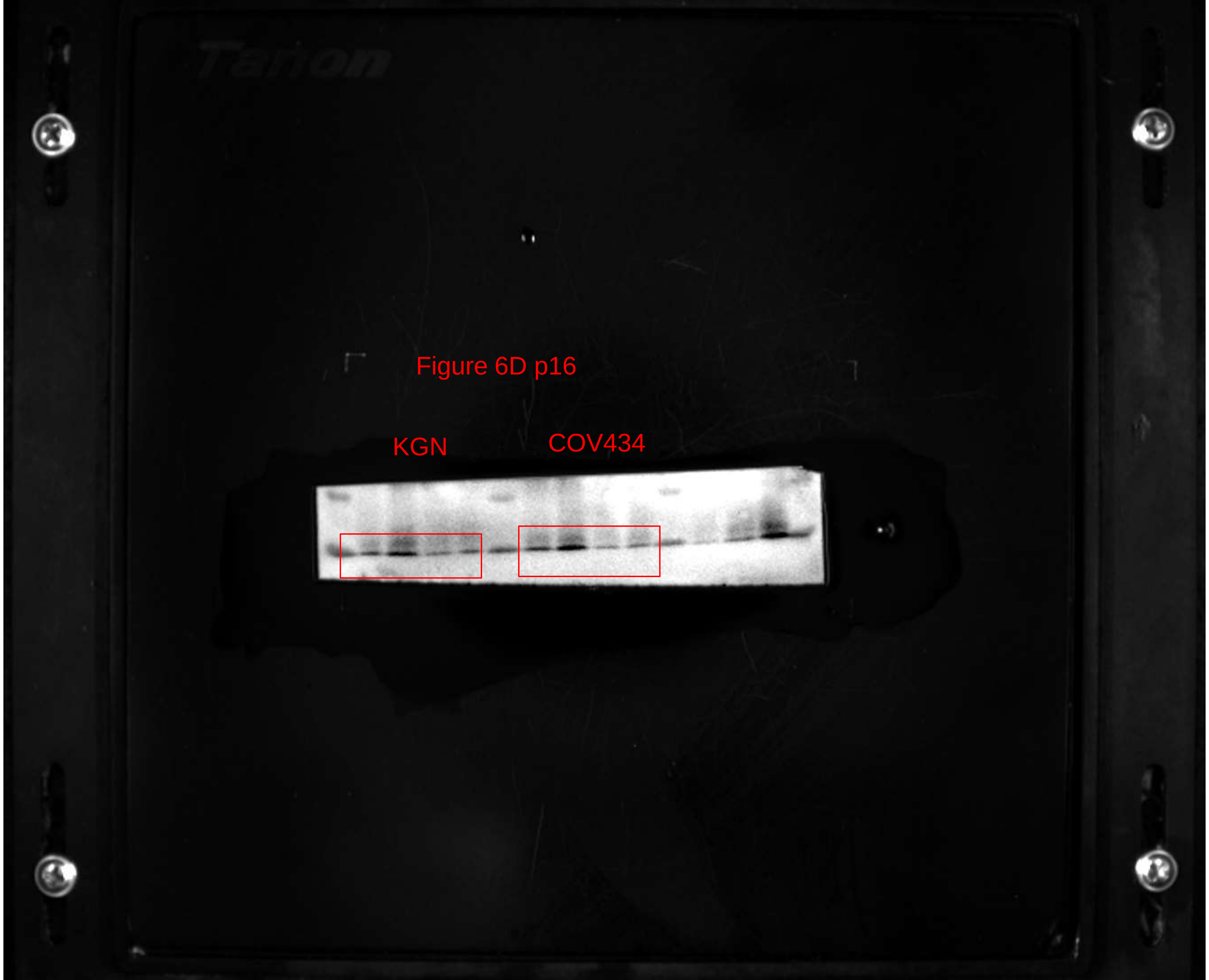
Figure 6D p16
KGN
COV434

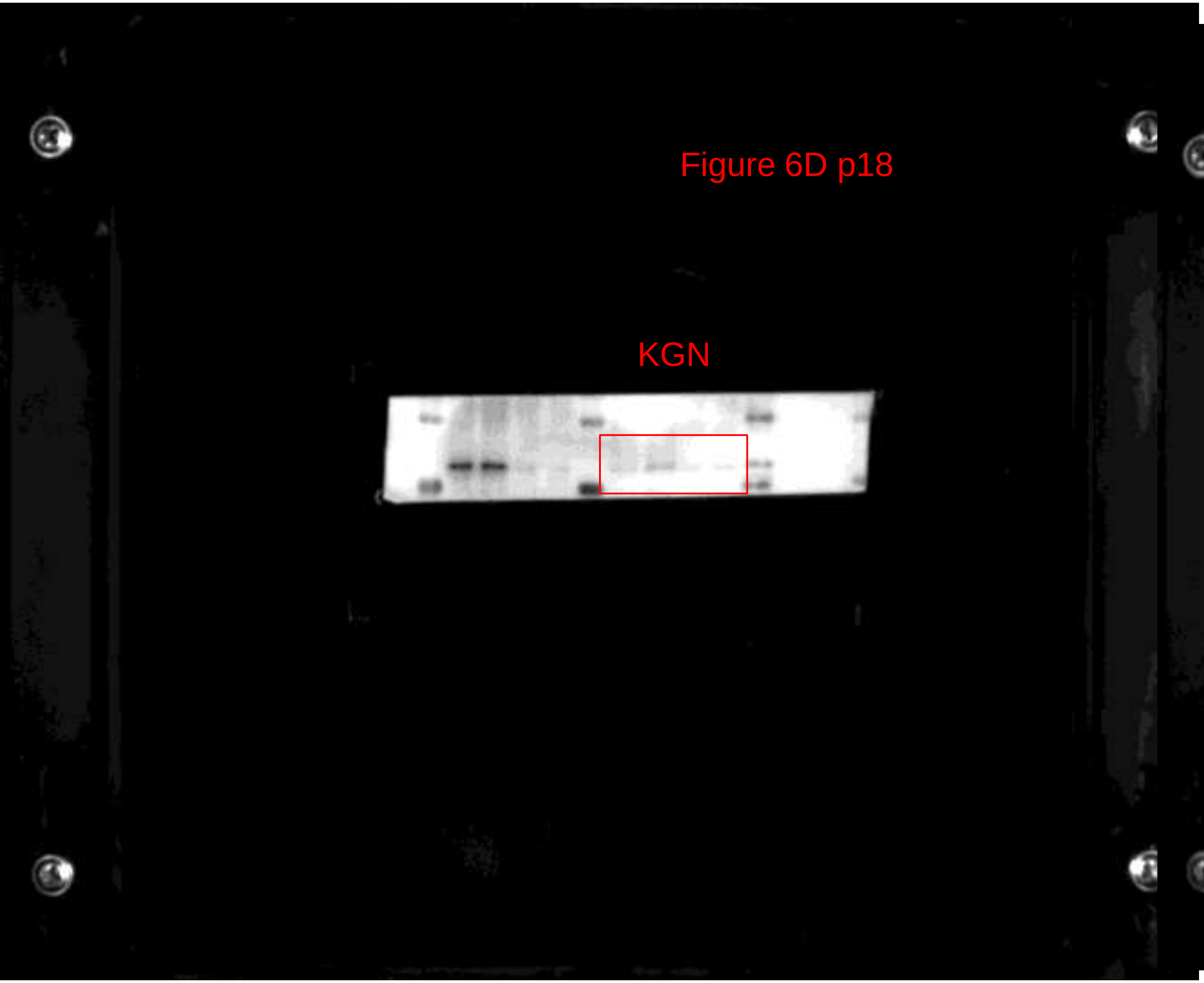
Figure 6D p18
KGN

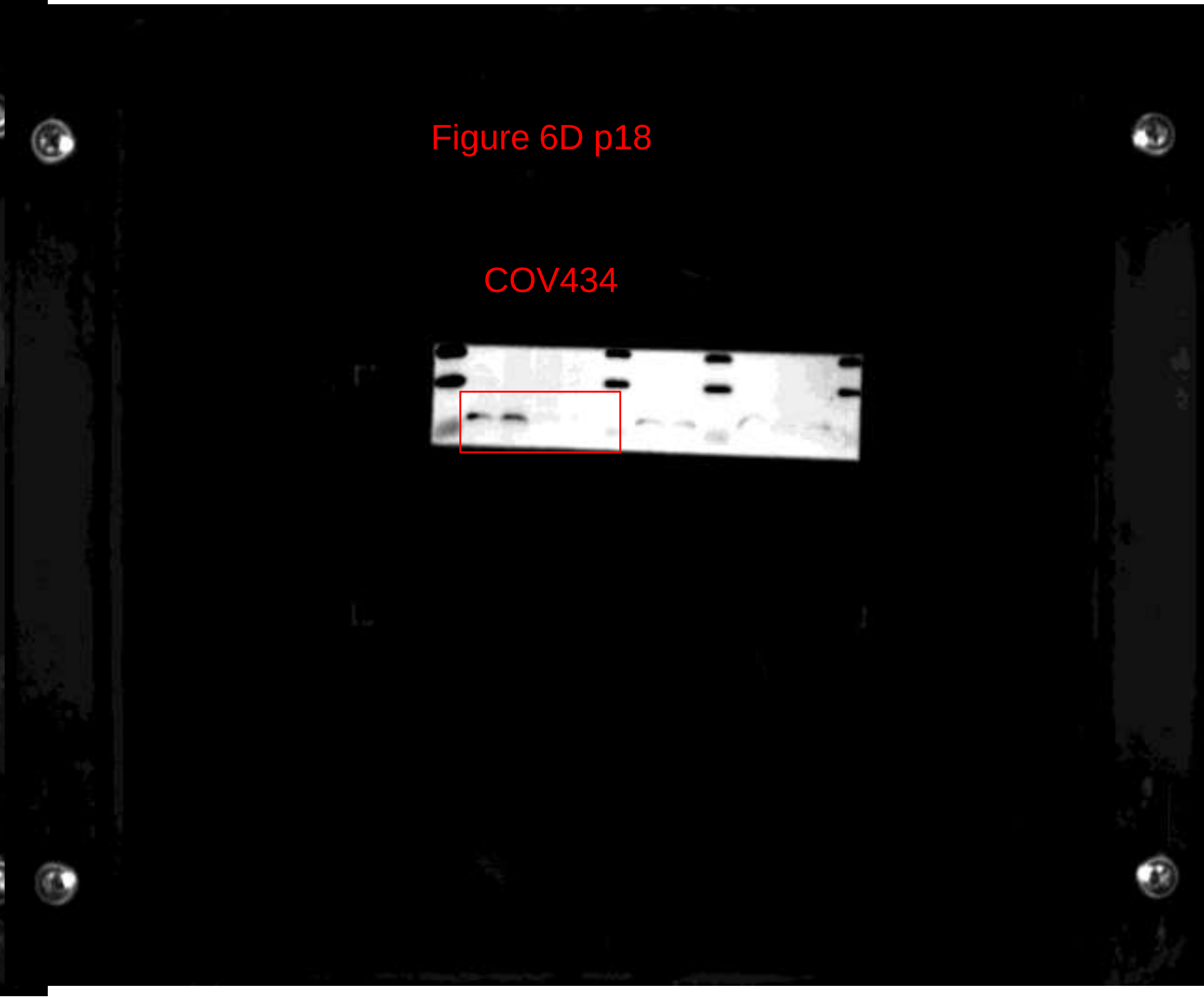
Figure 6D p18
COV434

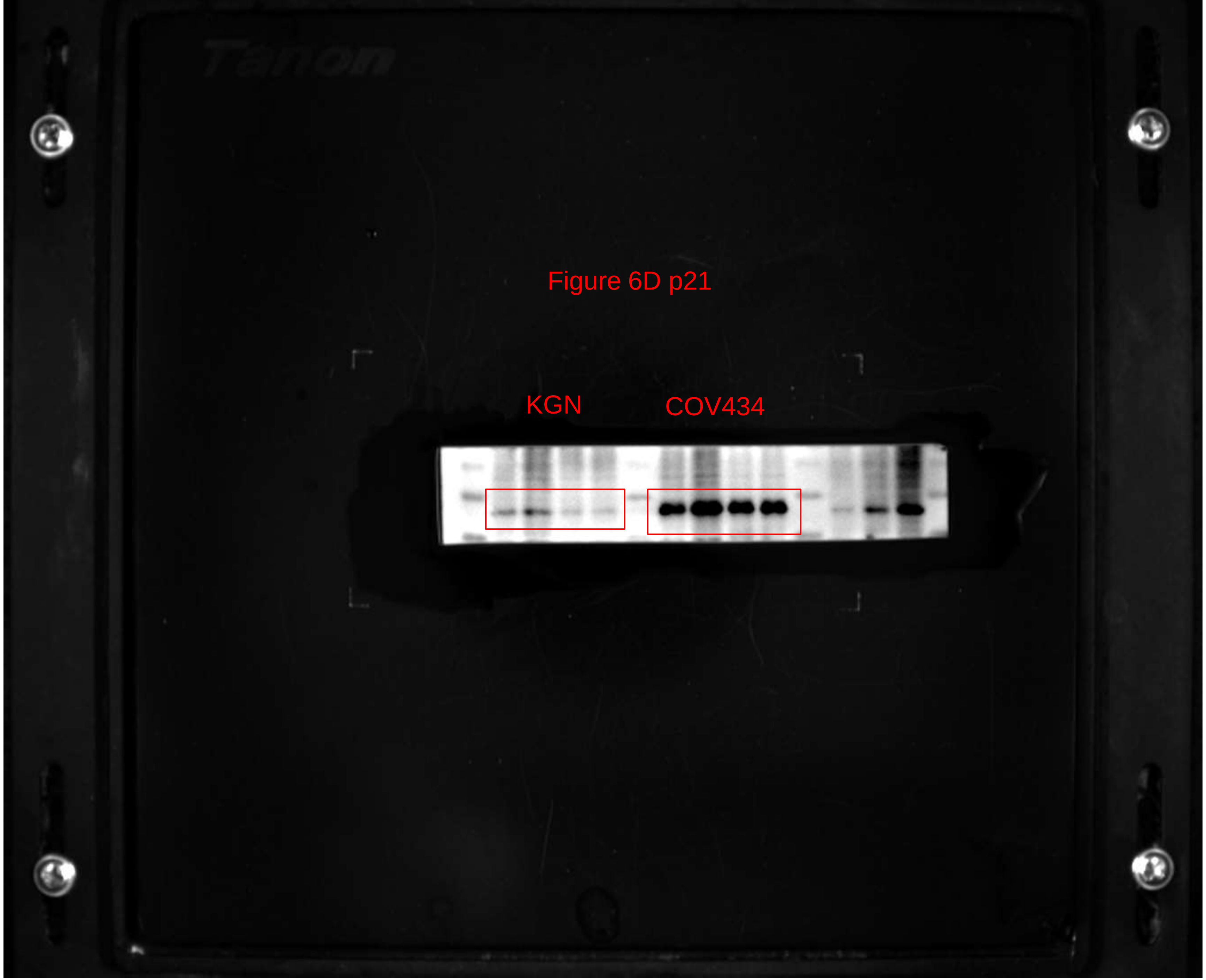
Figure 6D p21
KGN
COV434

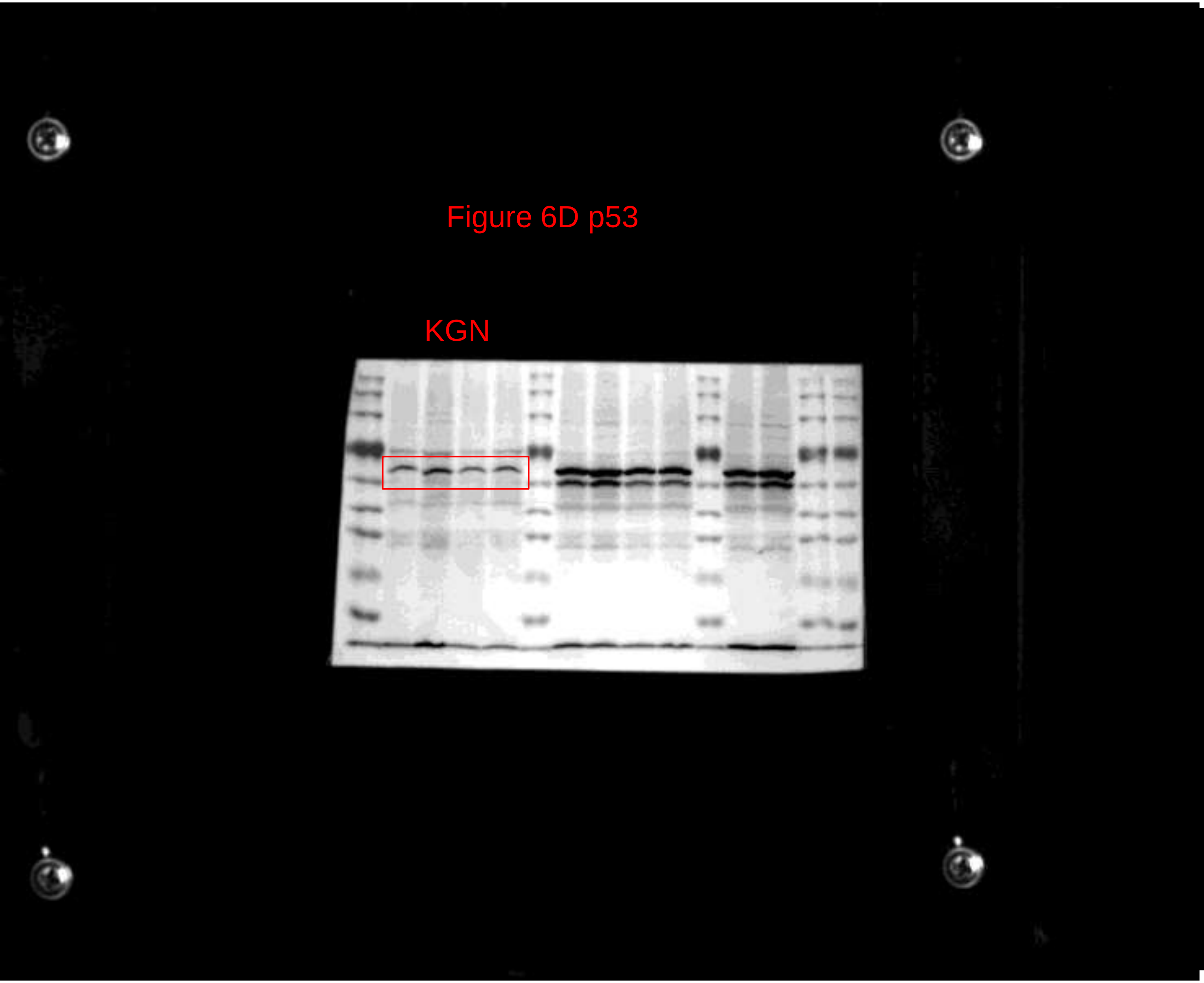
Figure 6D p53
KGN

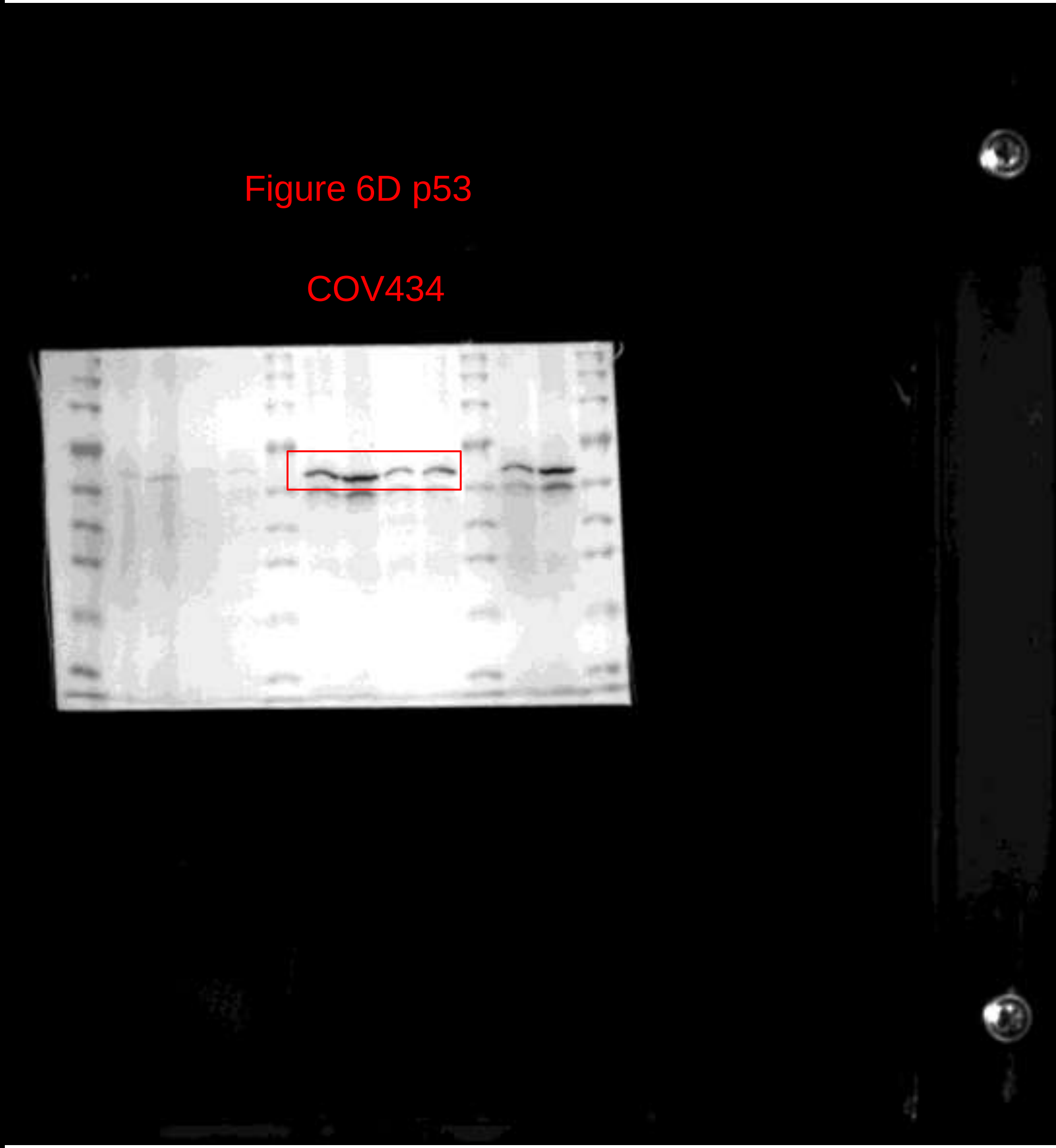
Figure 6D p53
COV434

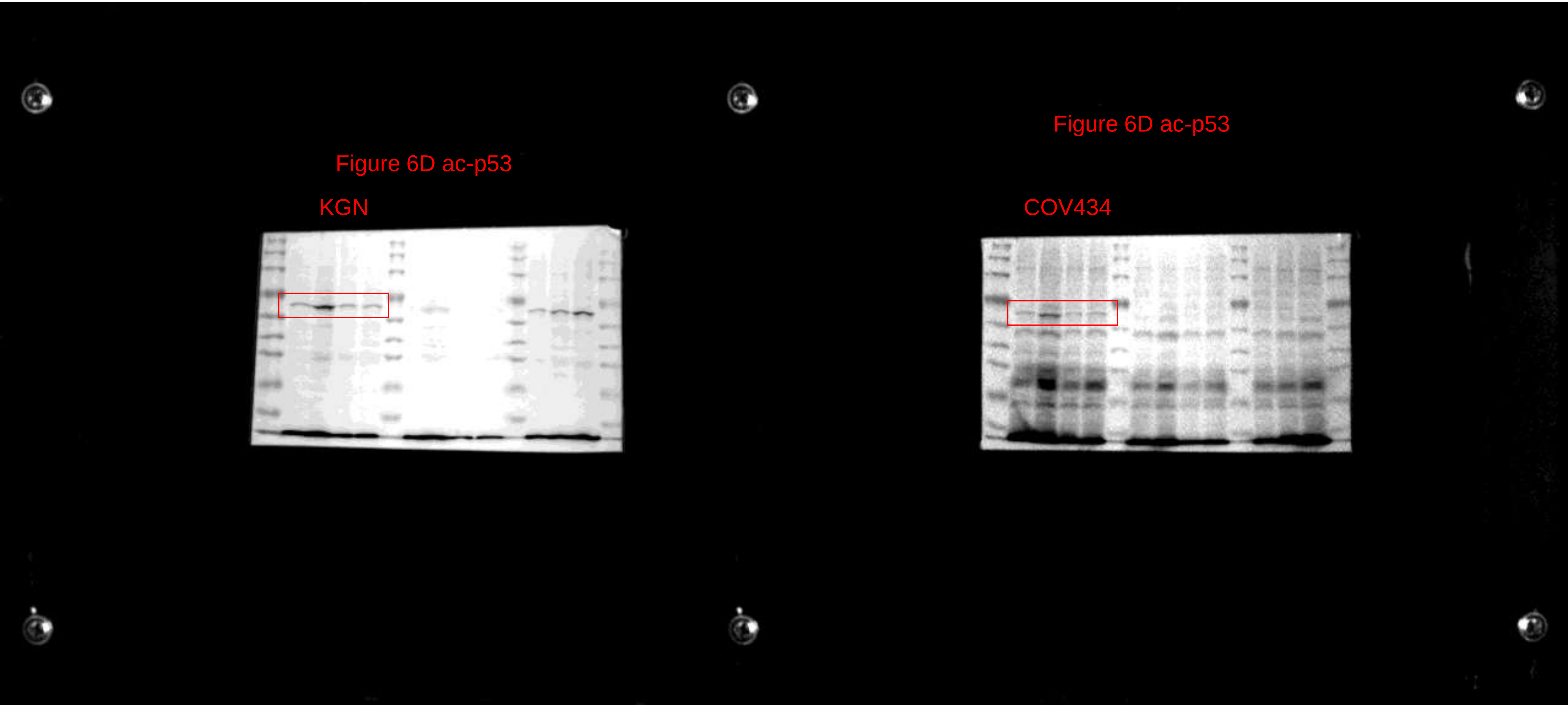
Figure 6D ac-p53
KGN
Figure 6D ac-p53
COV434

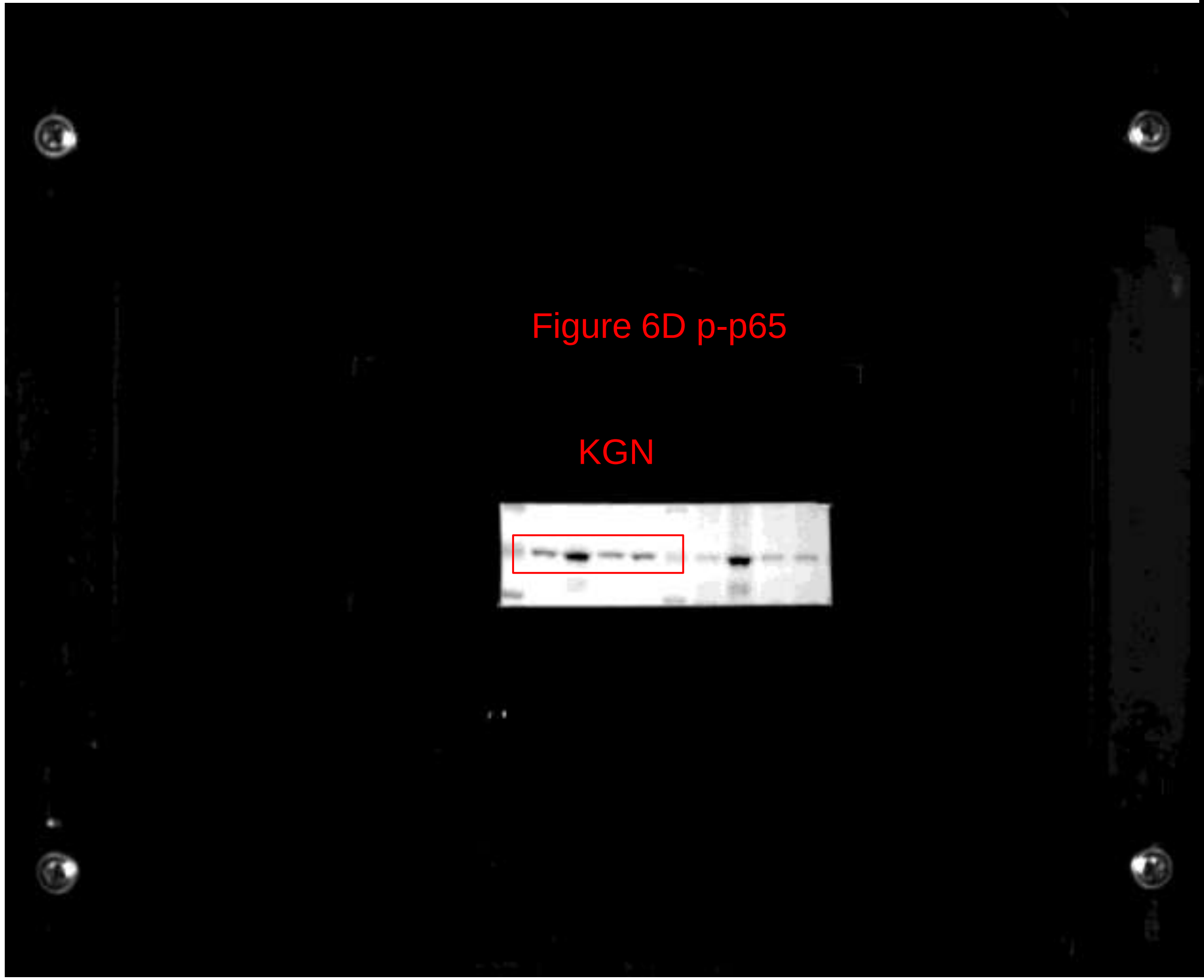
Figure 6D p-p65
KGN

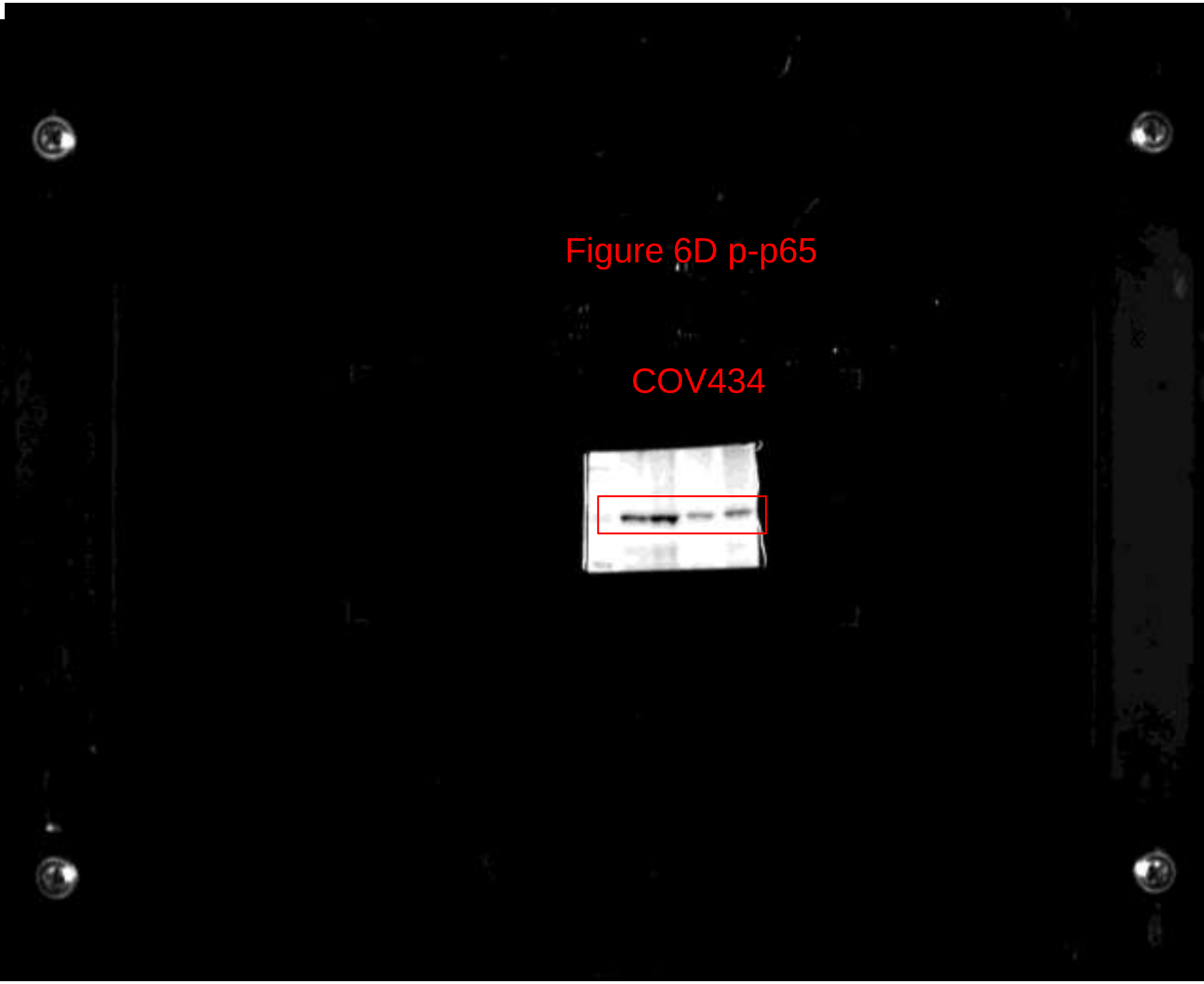
Figure 6D p-p65
COV434

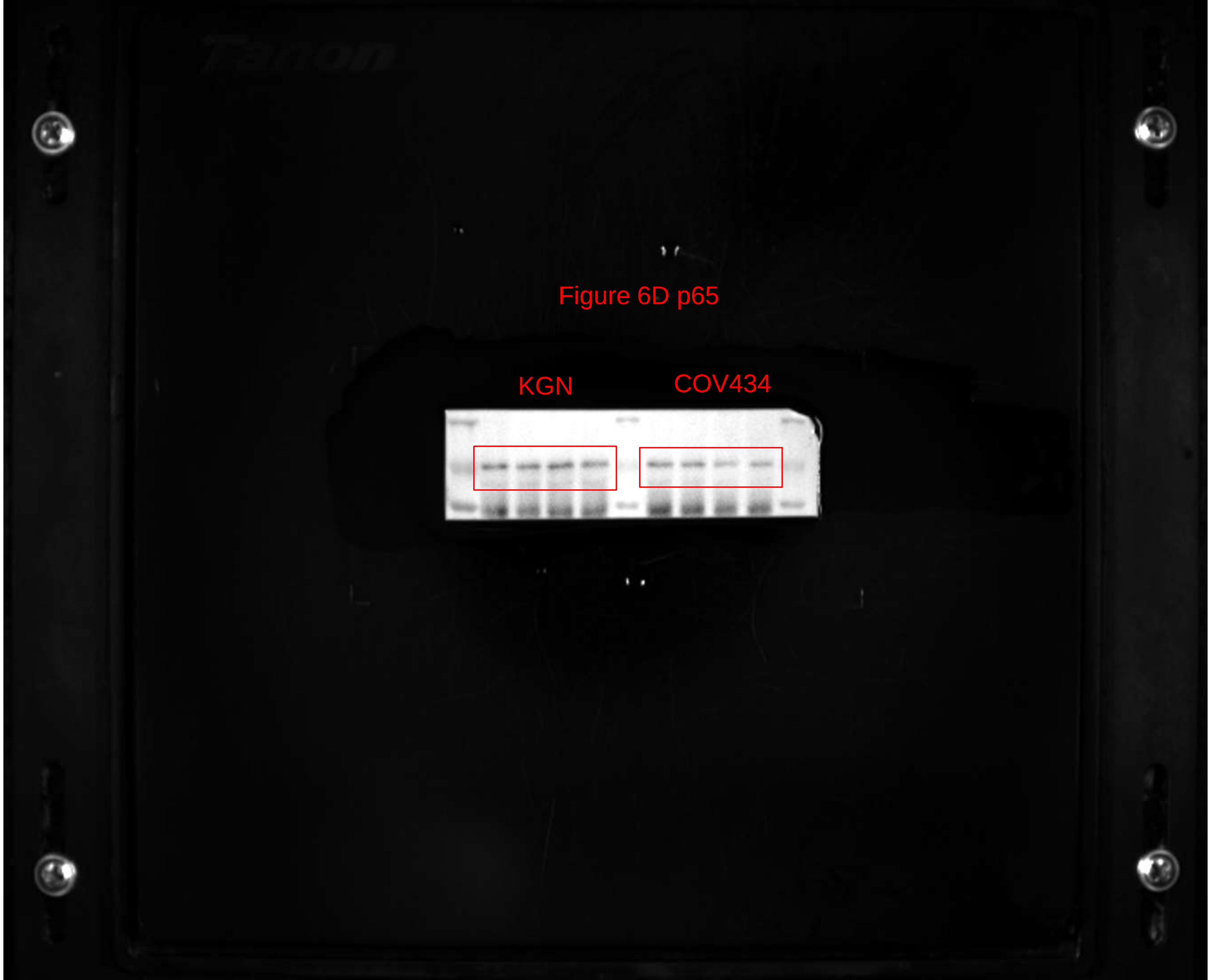
Figure 6D p65
KGN
COV434

Figure 6D p-IκBα

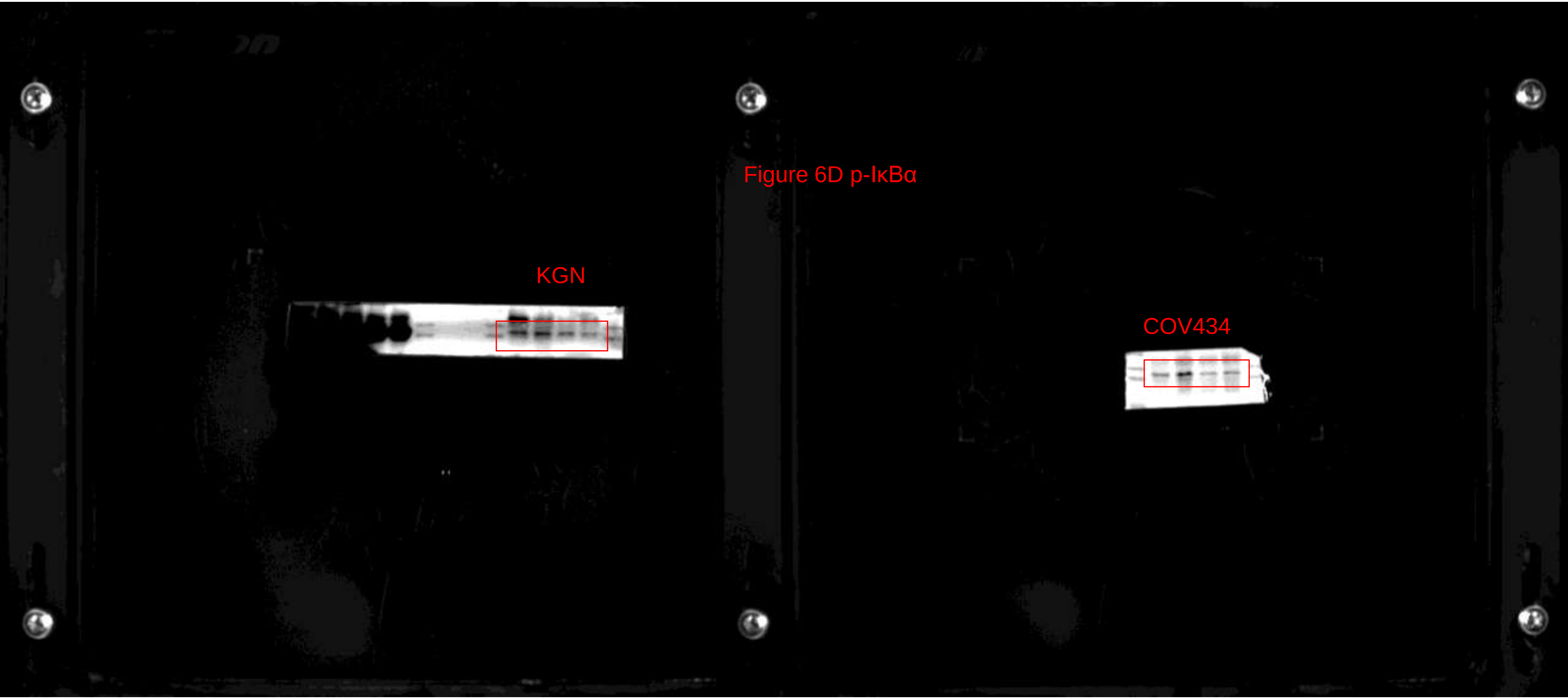

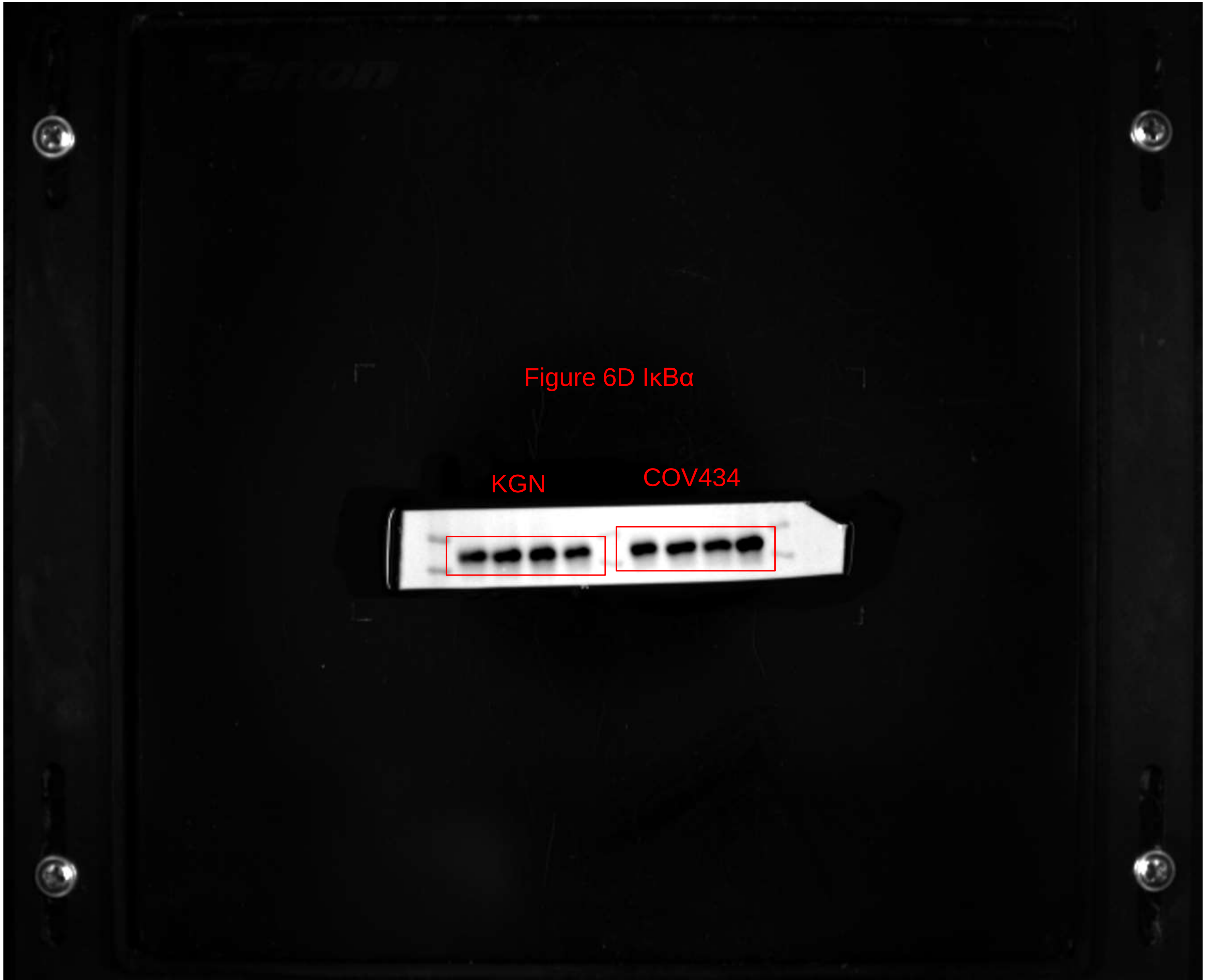
Figure 6D IκBα
KGN
COV434

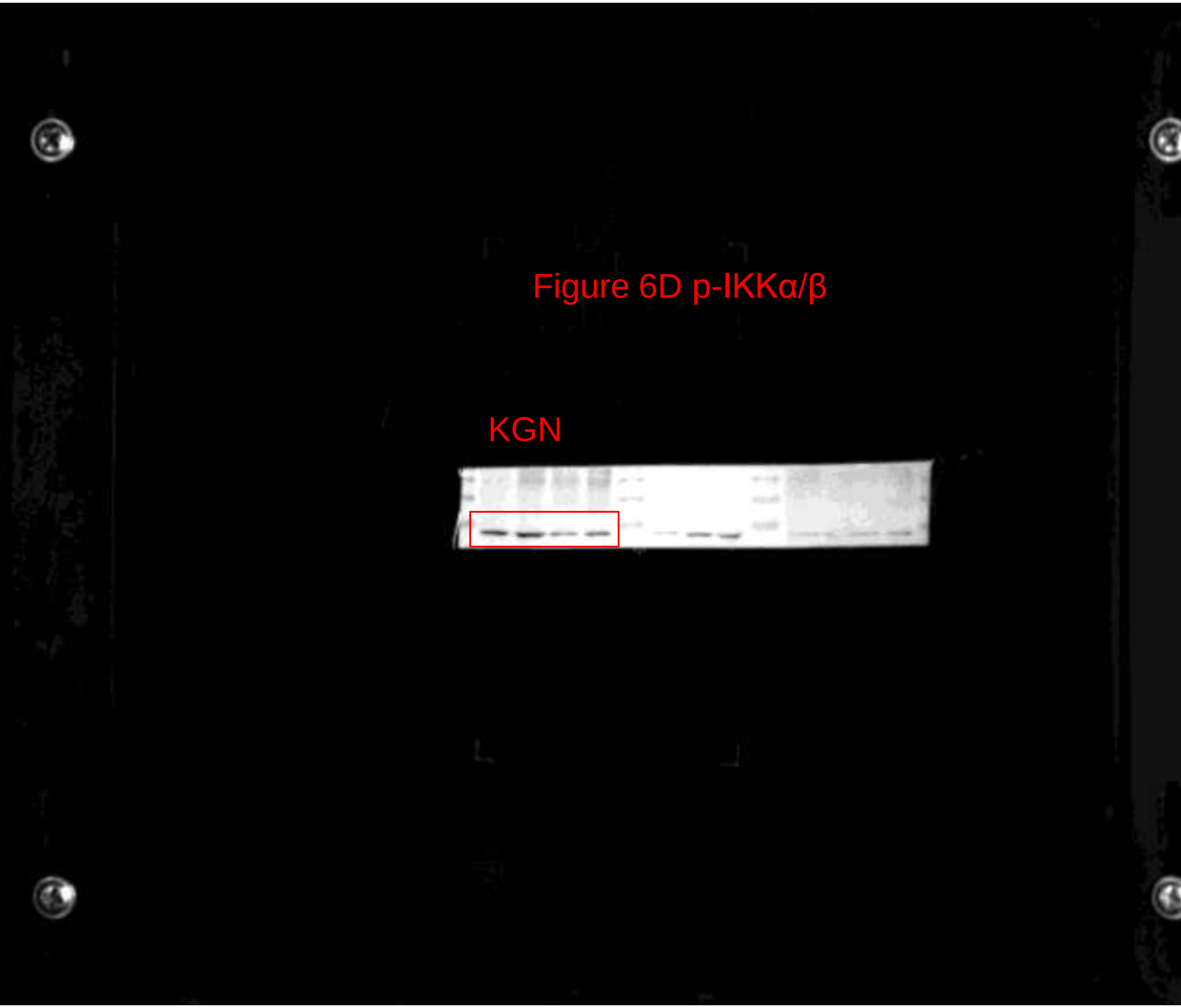
Figure 6D p-IKKα/β
KGN

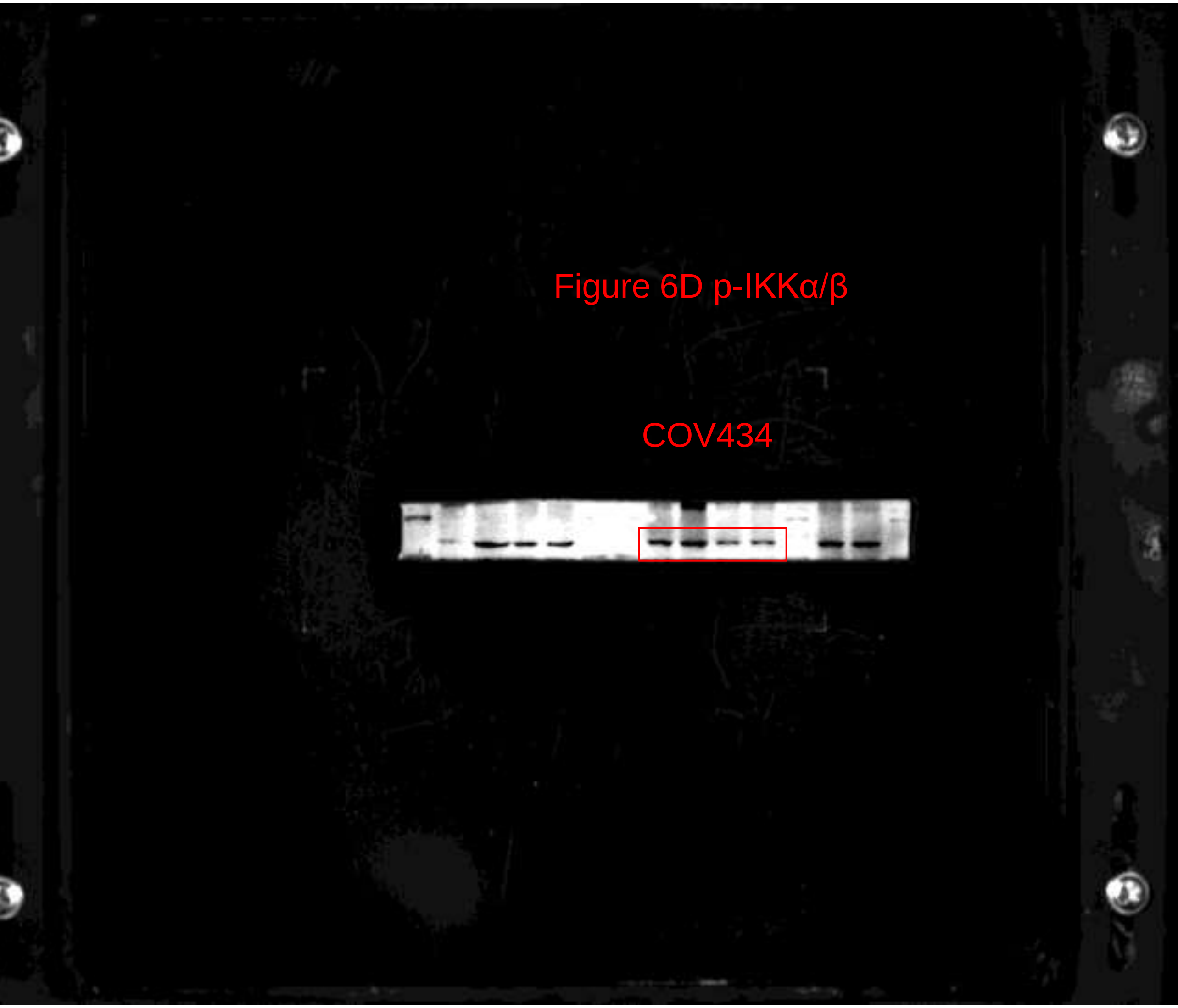
Figure 6D p-IKKα/β
COV434

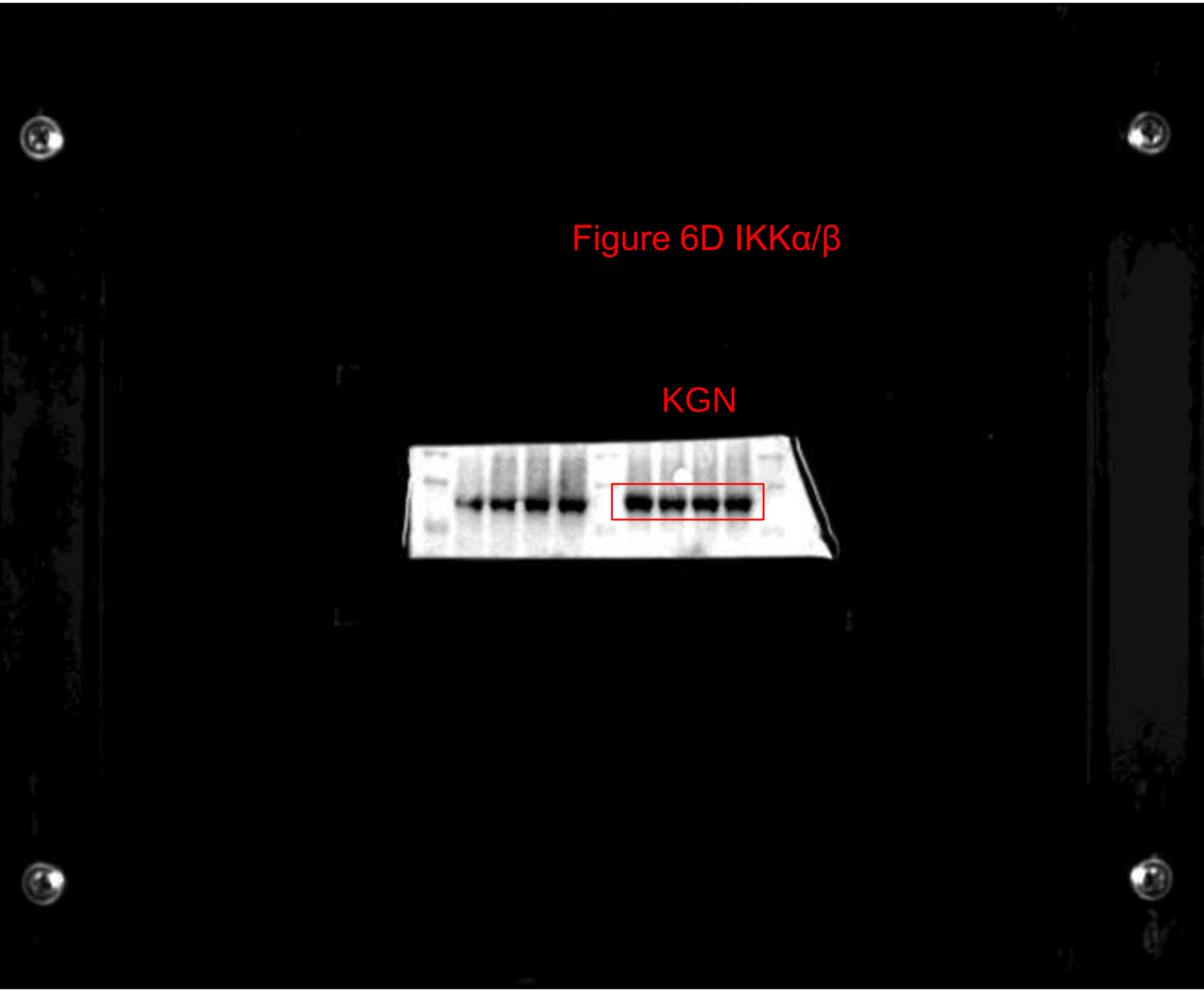
Figure 6D IKKα/β
KGN
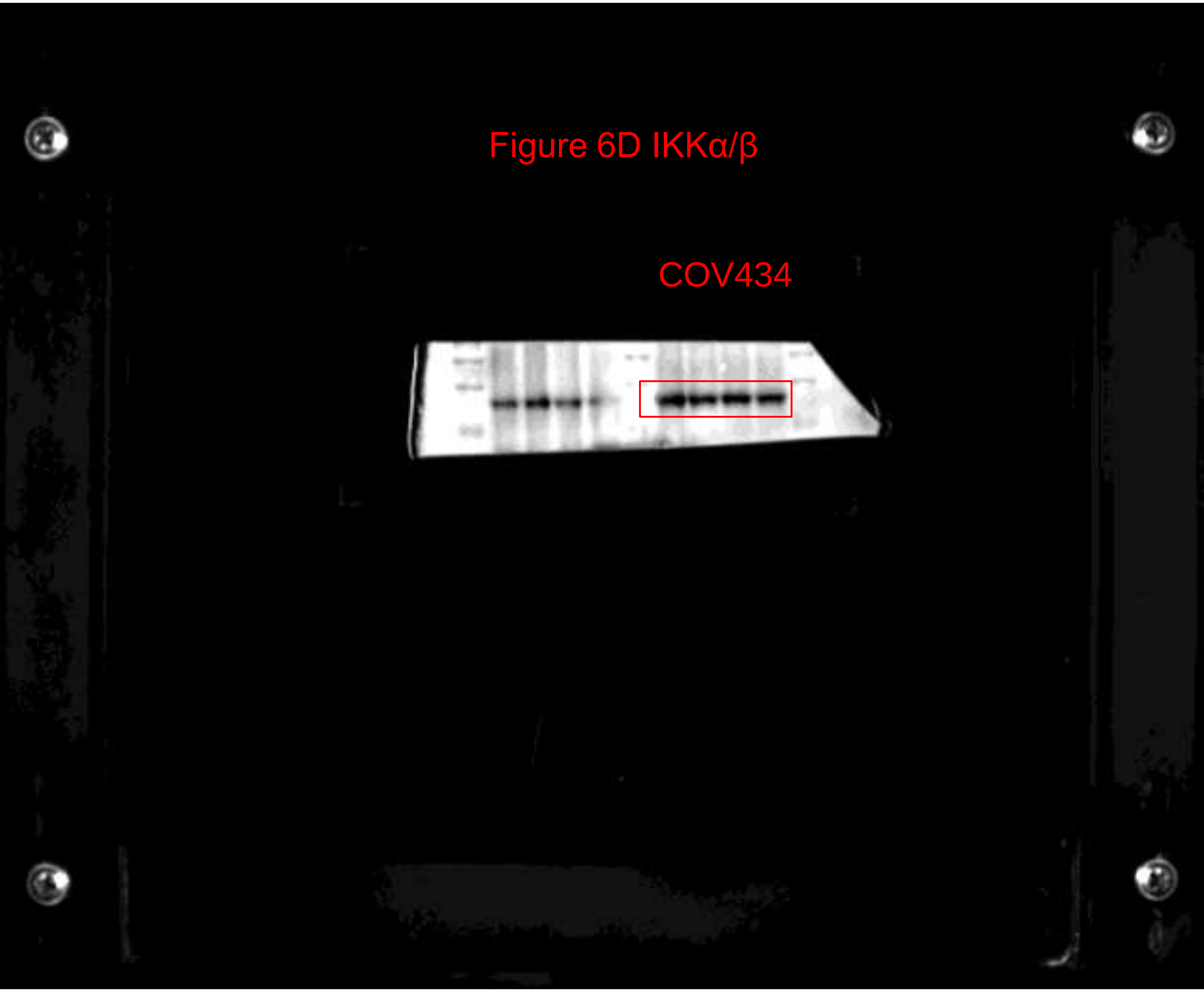
Figure 6D IKKα/β
COV434

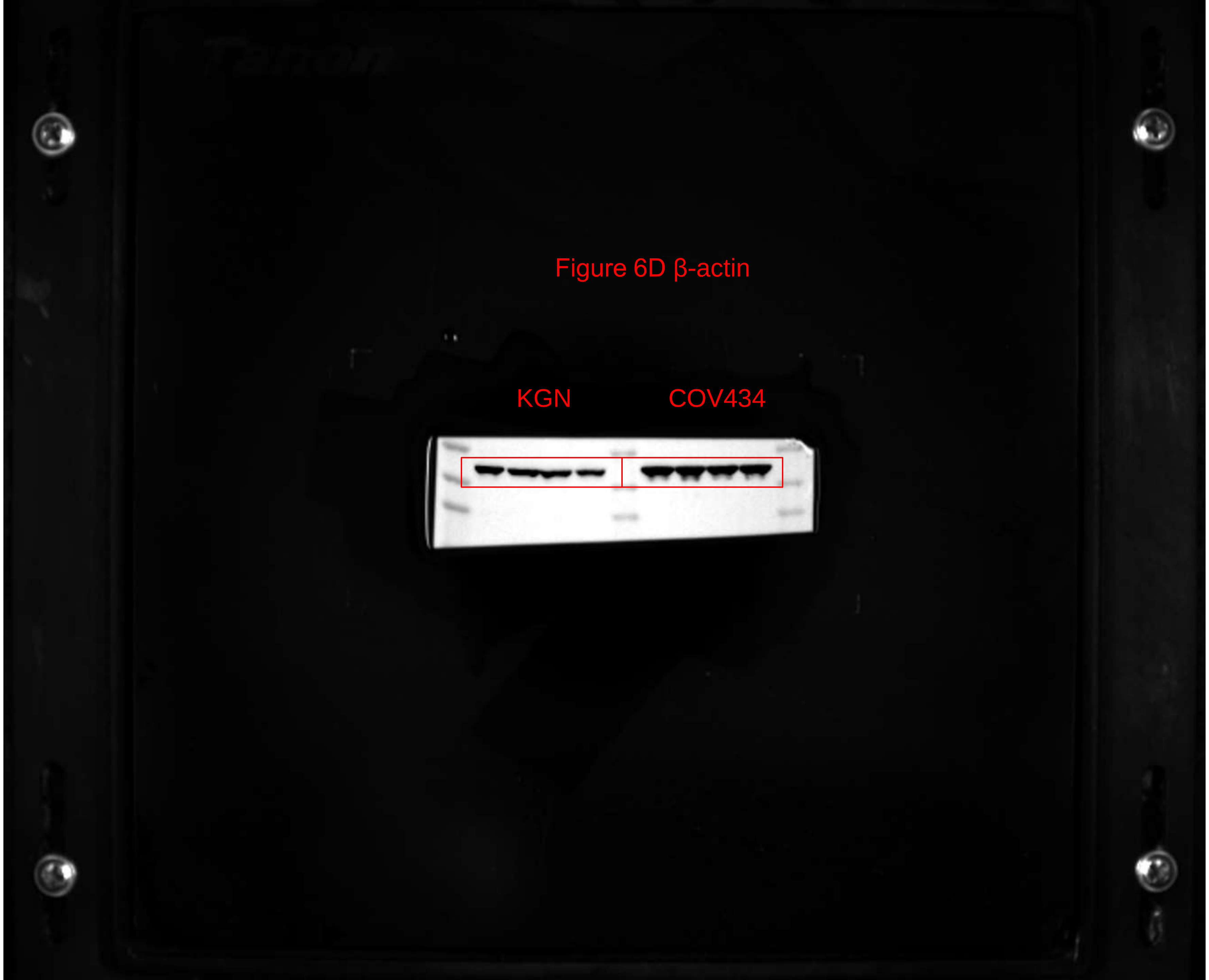
Figure 6D β-actin
KGN
COV434

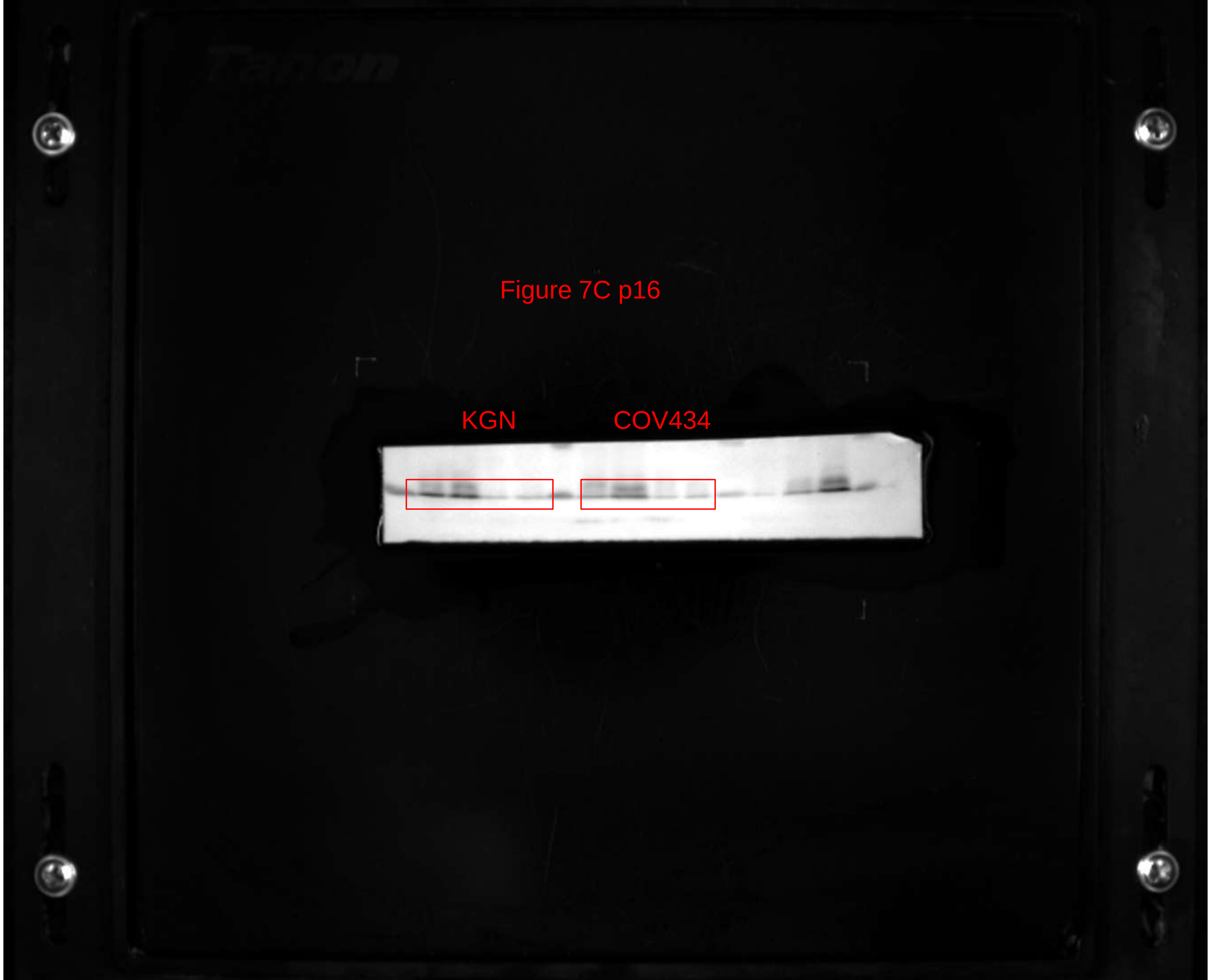
Figure 7C p16
KGN
COV434

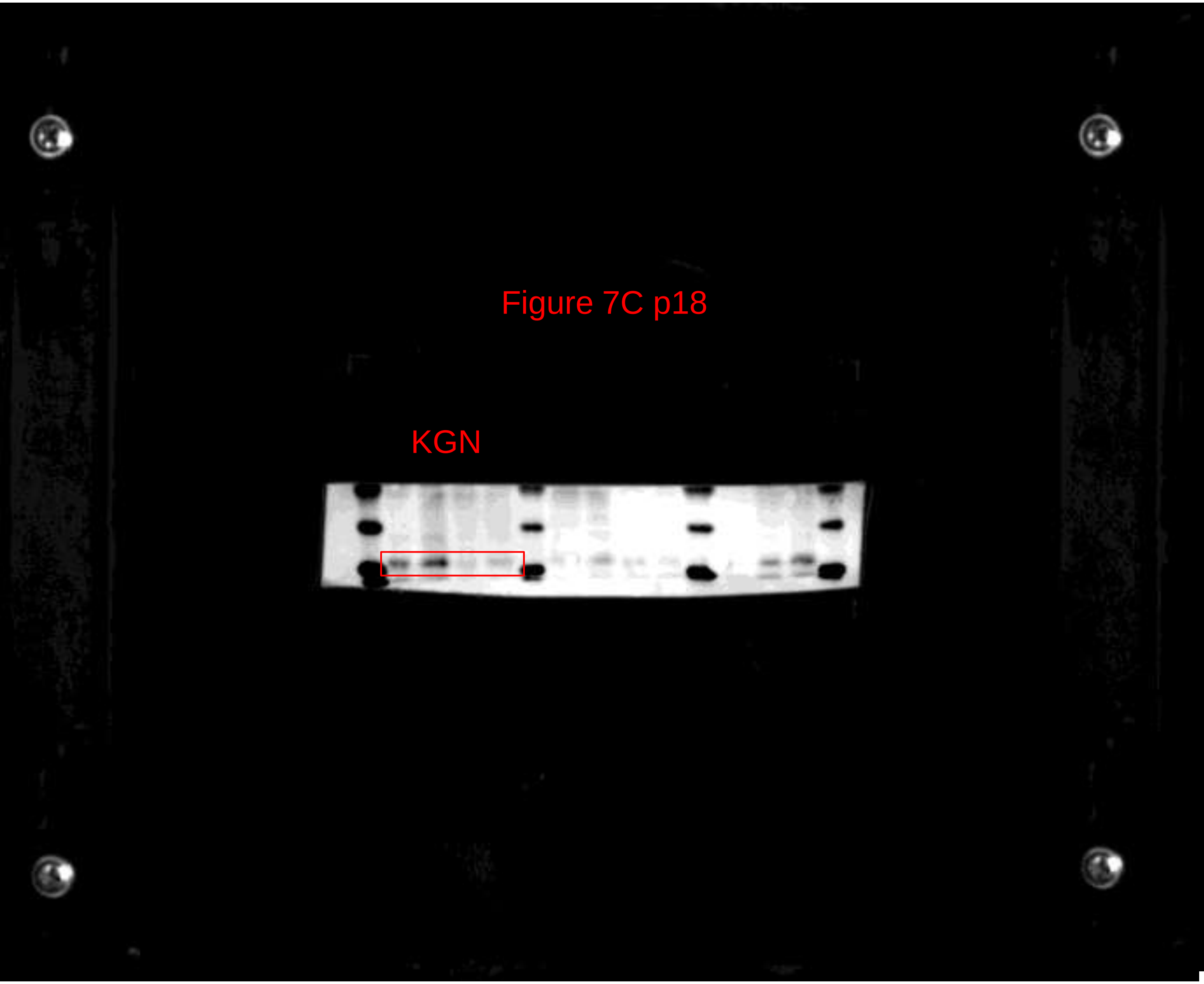
Figure 7C p18
KGN

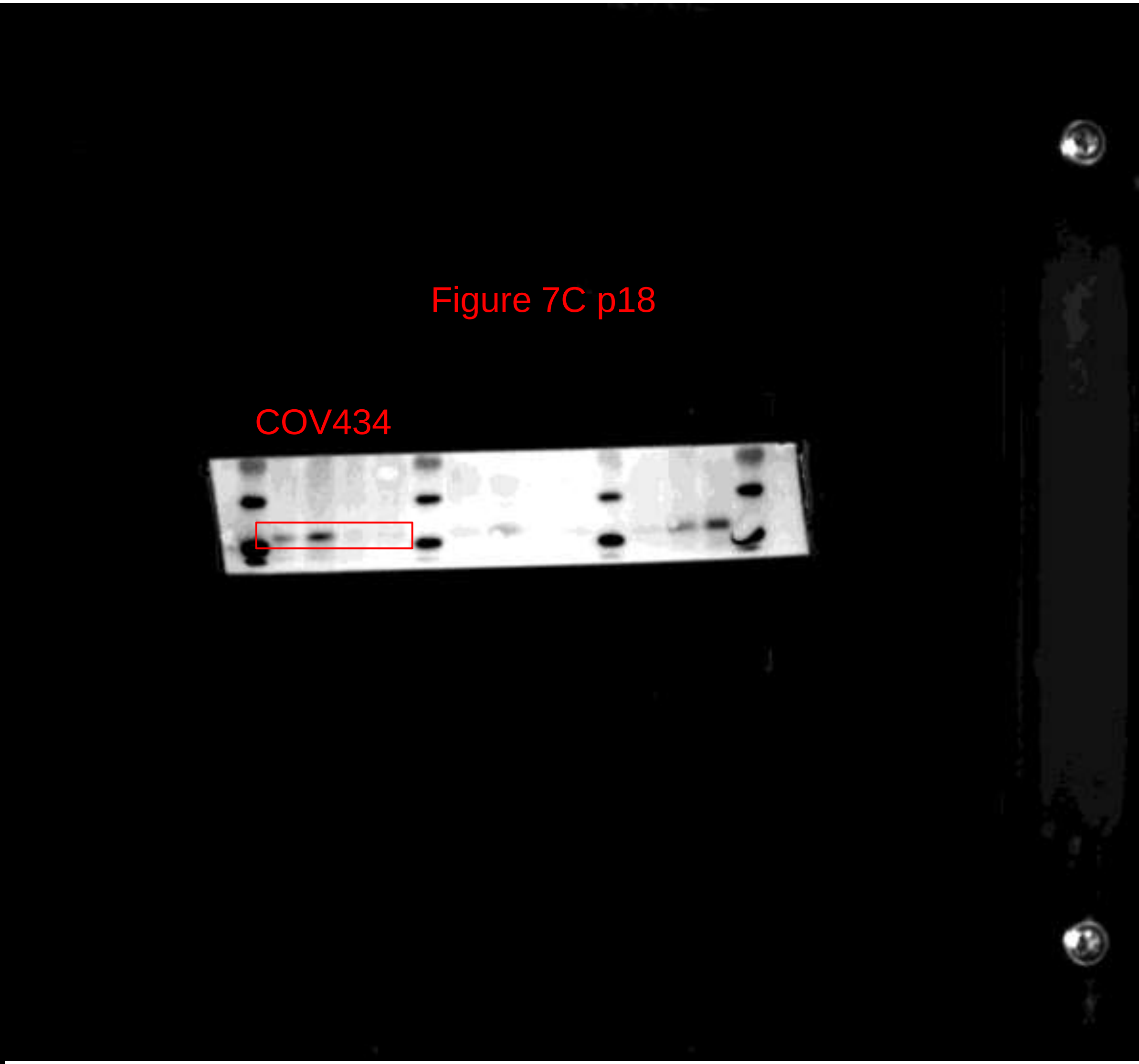
Figure 7C p18
COV434

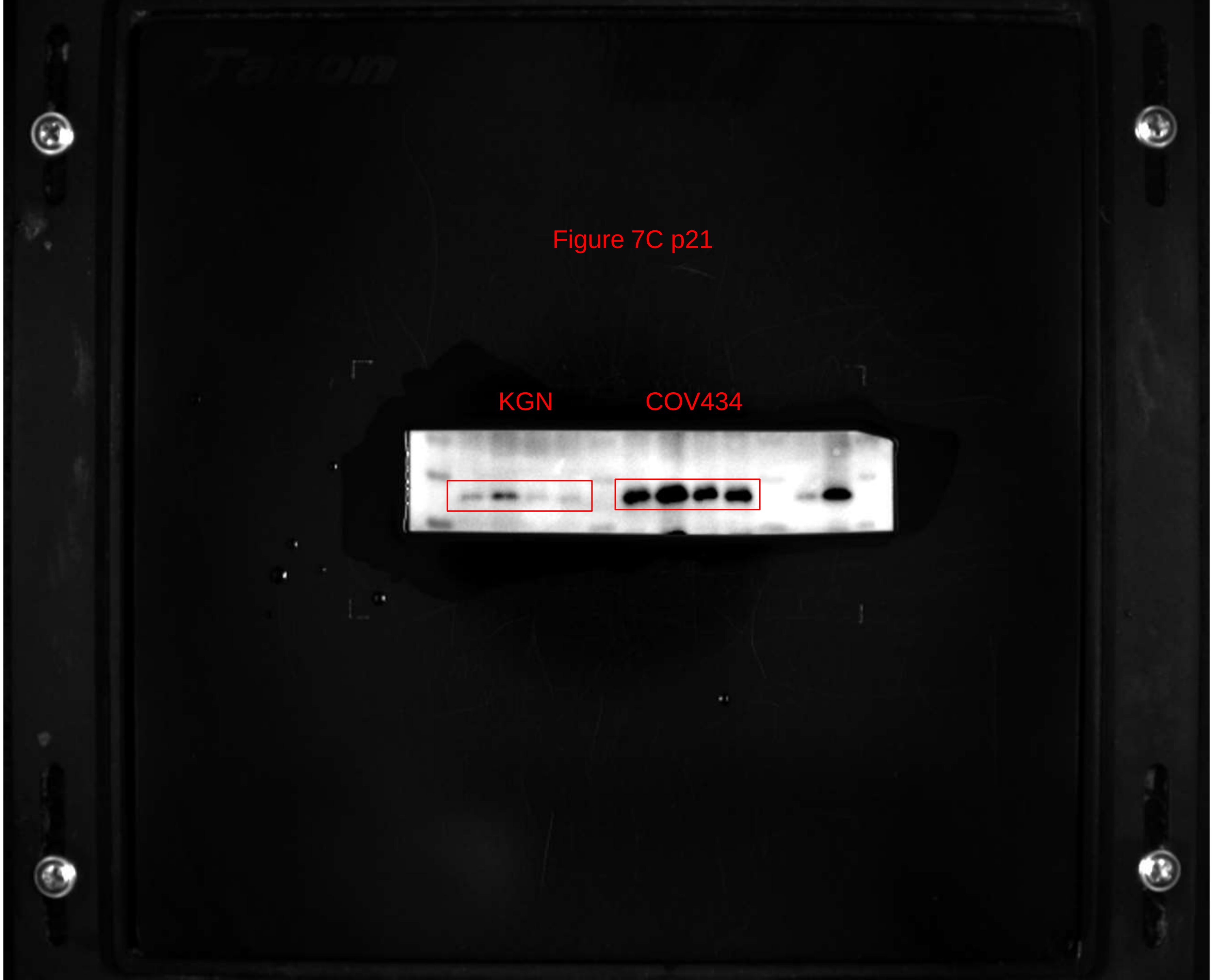
Figure 7C p21
KGN
COV434

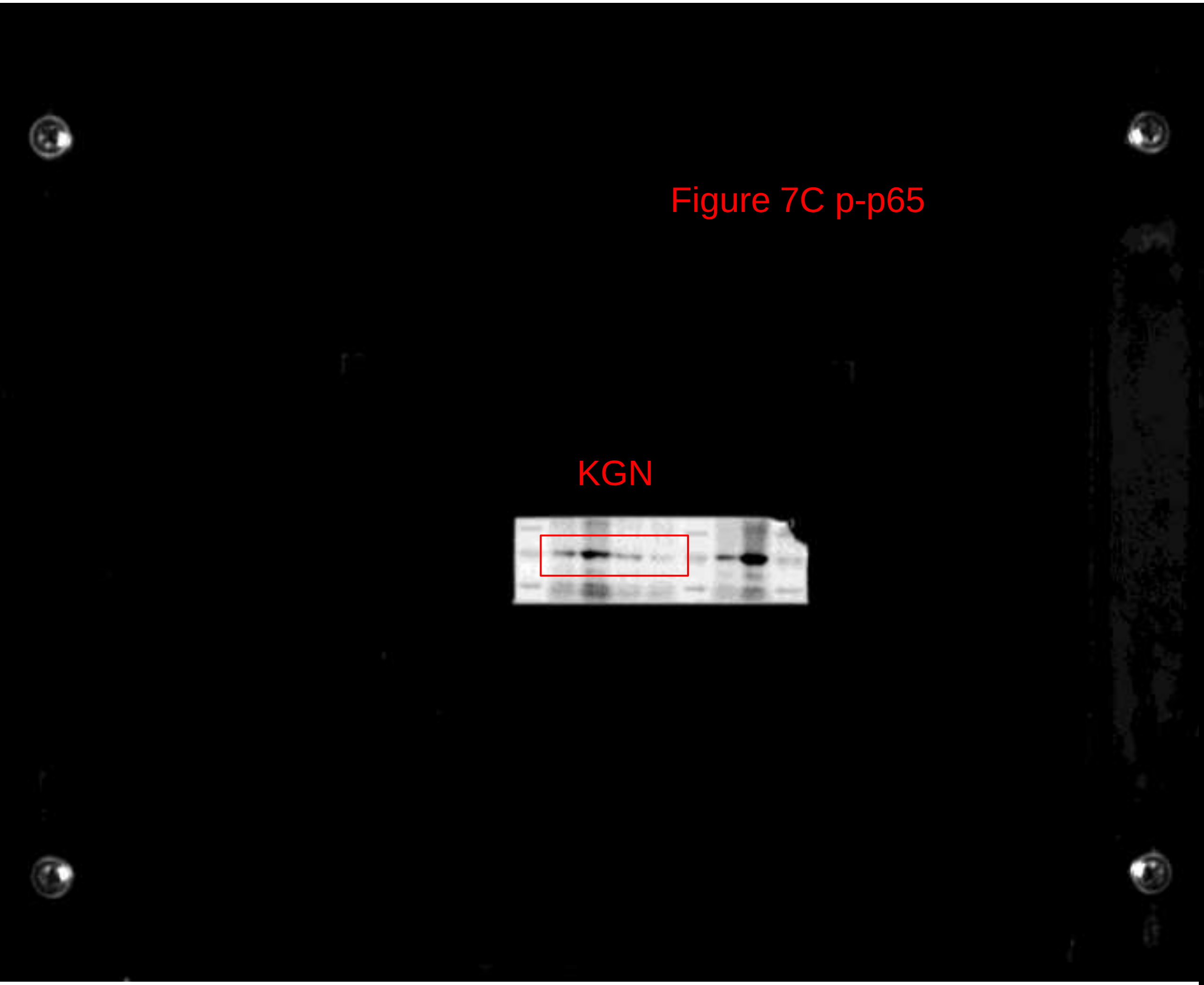
Figure 7C p-p65
KGN

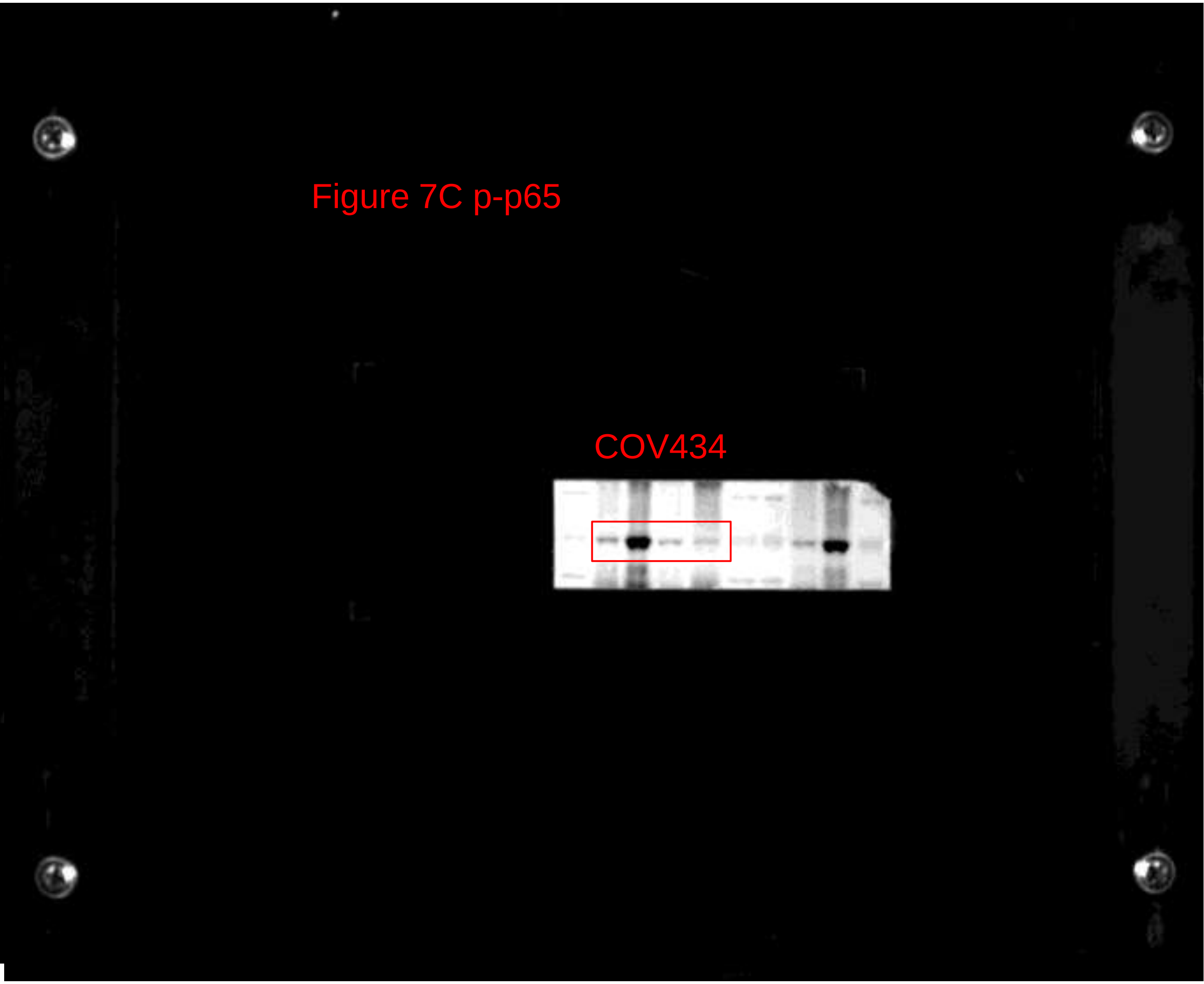
Figure 7C p-p65
COV434

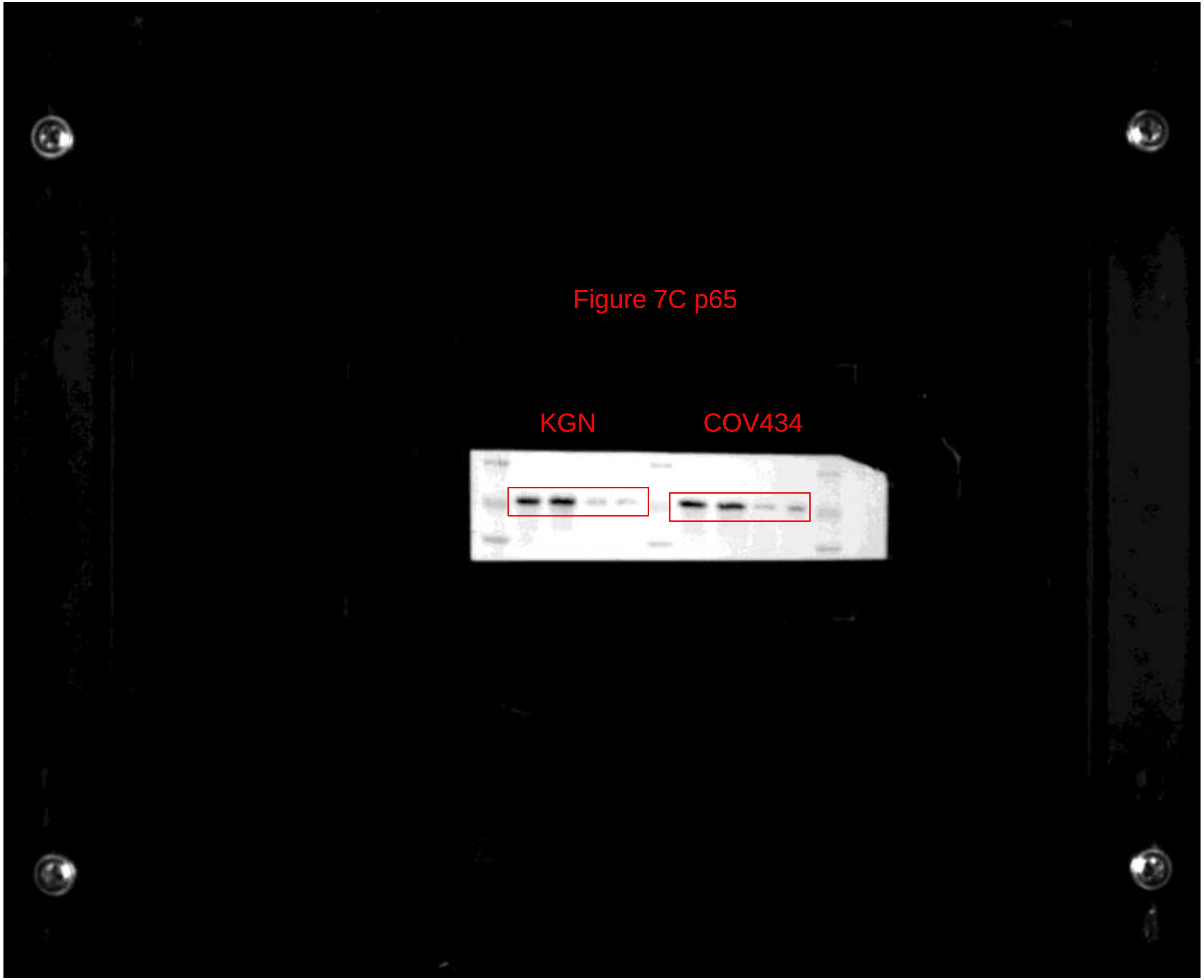
Figure 7C p65
KGN
COV434

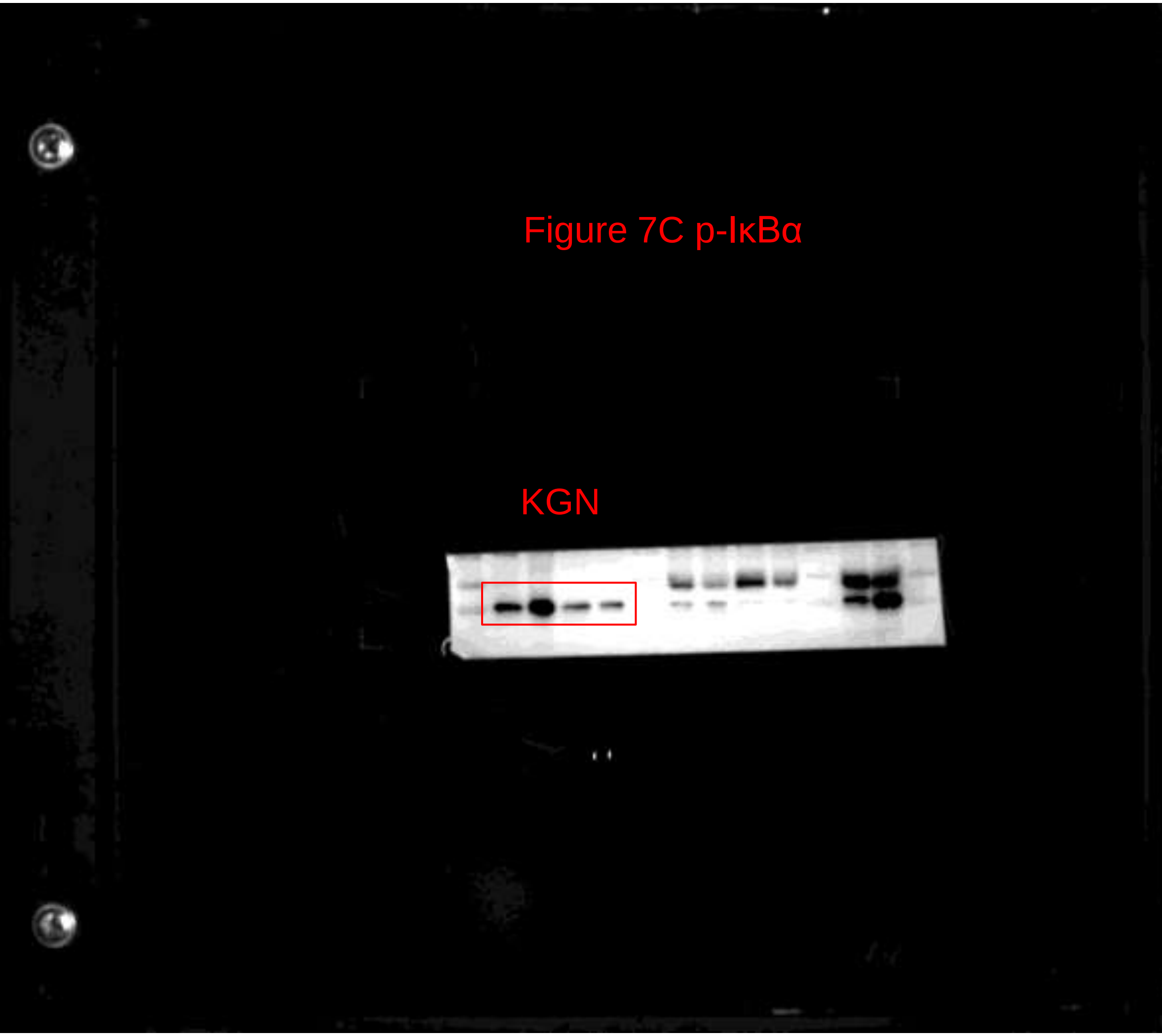
Figure 7C p-IκBα
KGN

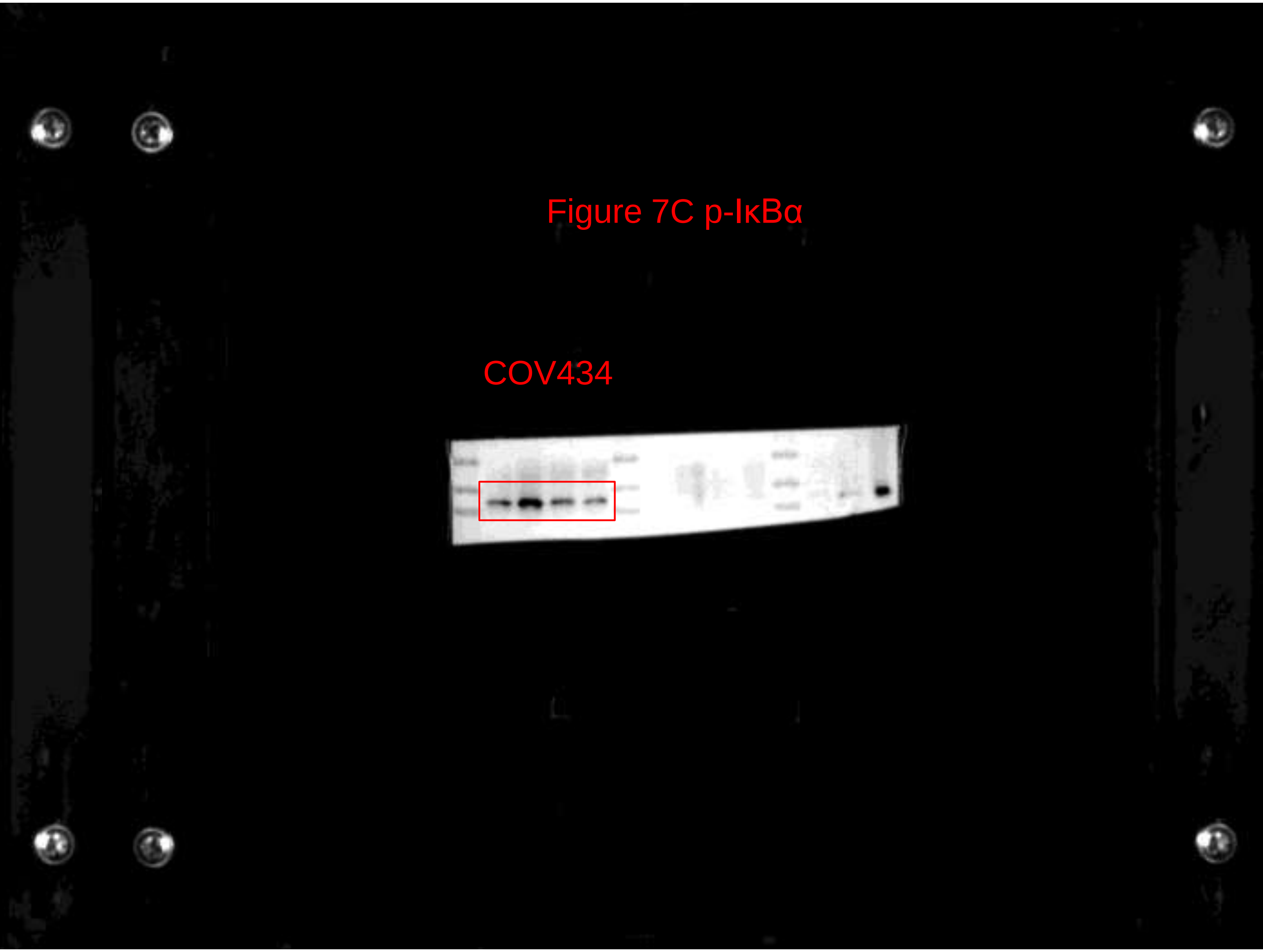
Figure 7C p-IκBα
COV434

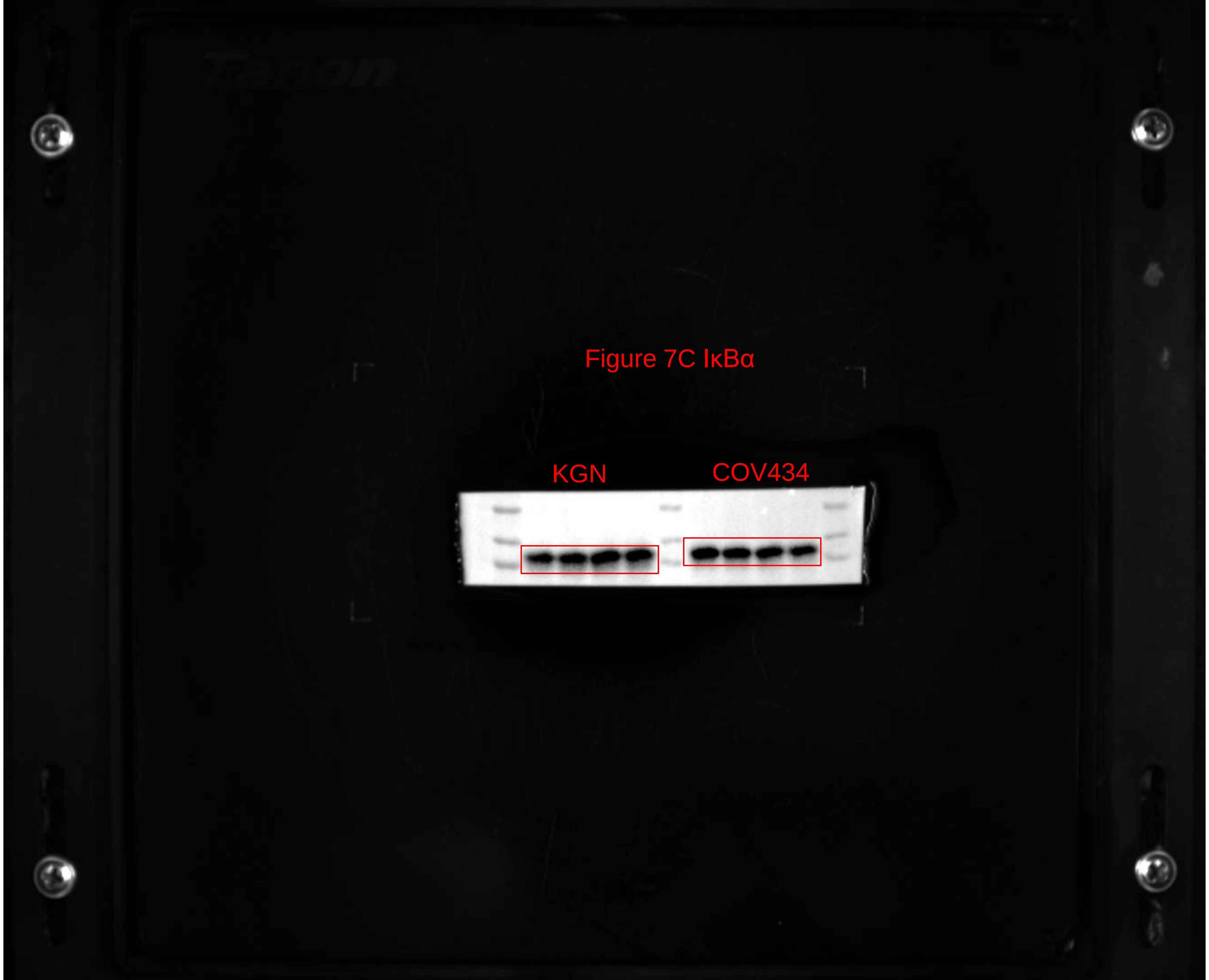
Figure 7C IκBα
KGN
COV434

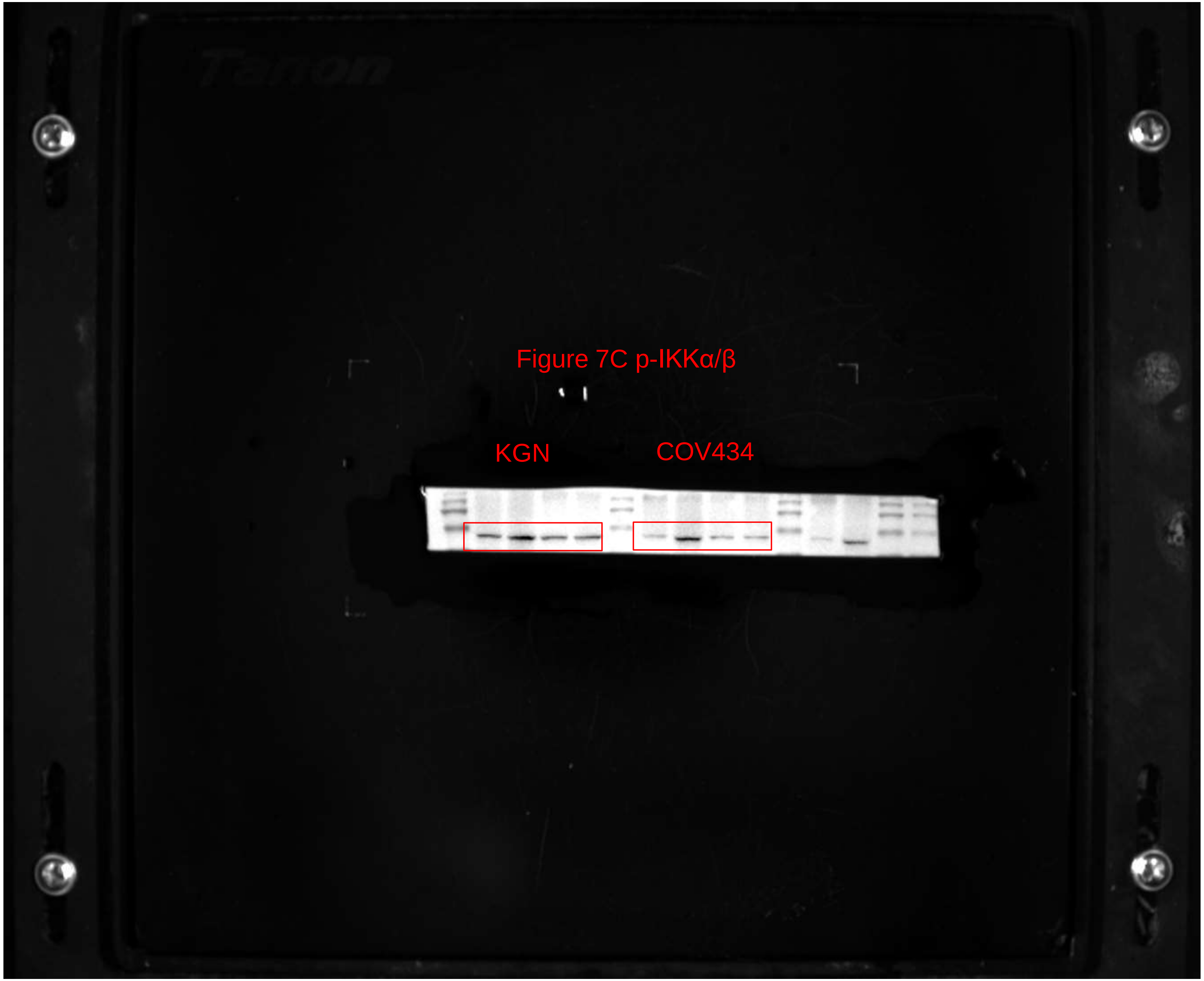
Figure 7C p-IKKα/β
KGN
COV434

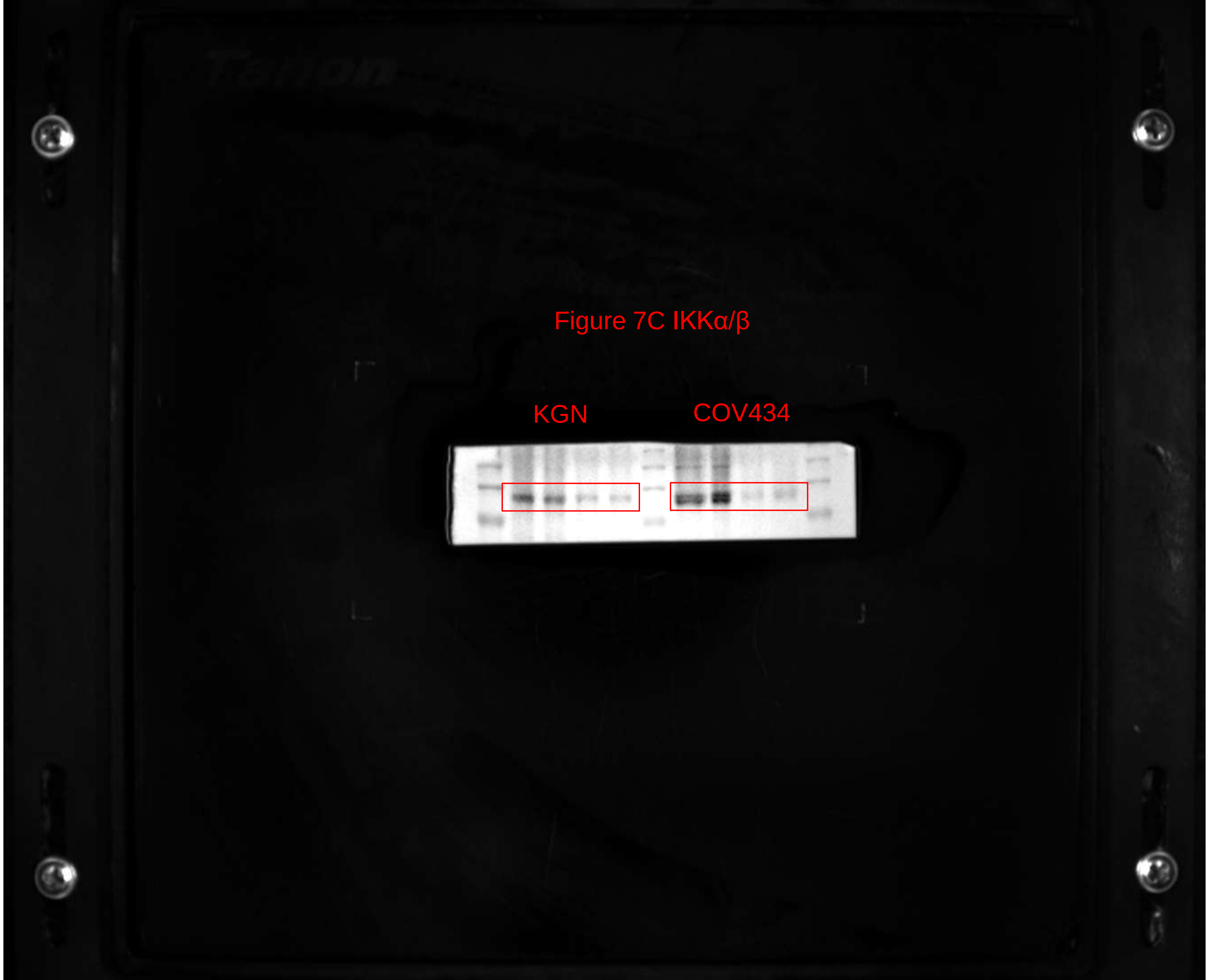
Figure 7C IKKα/β
KGN
COV434

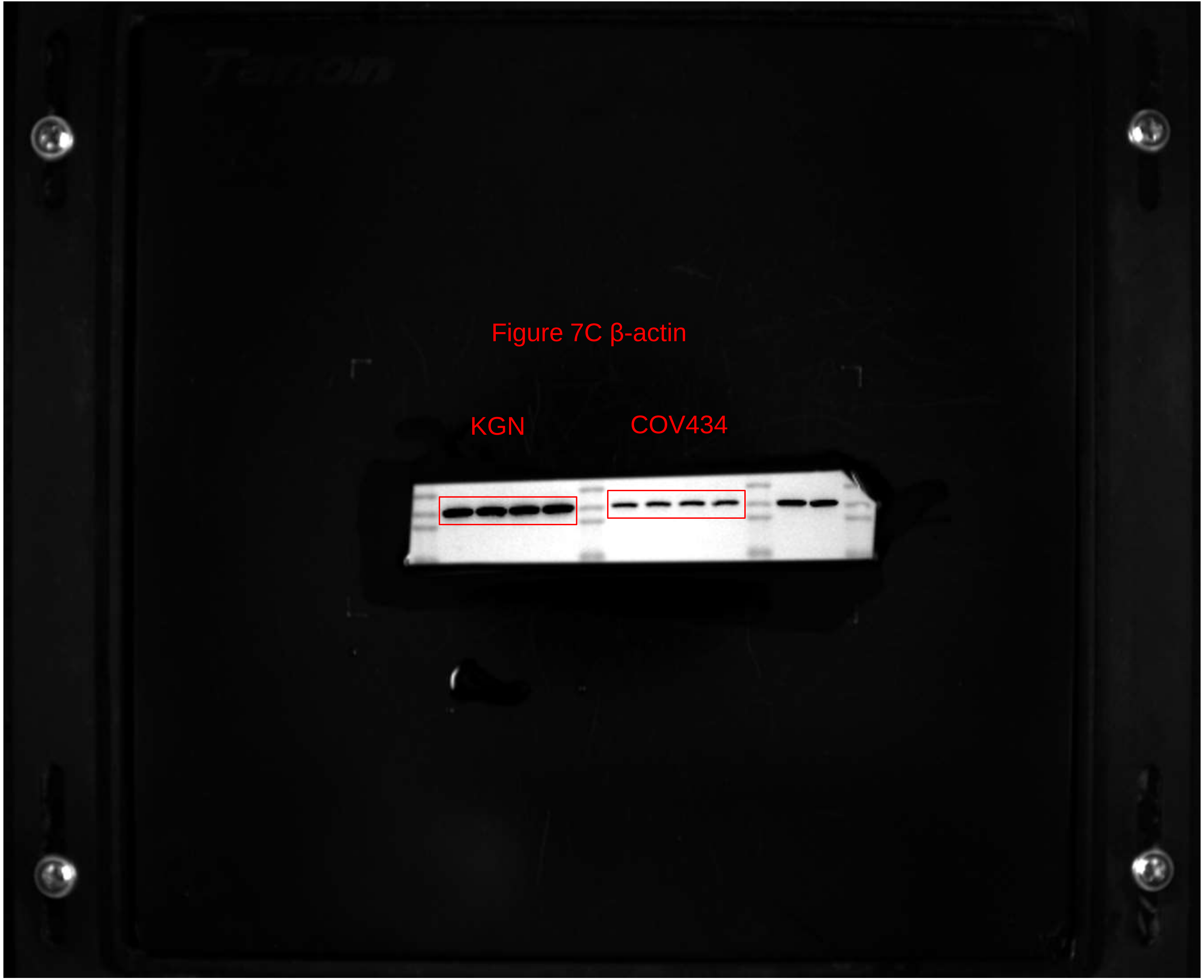
Figure 7C β-actin
KGN
COV434
